# Transcriptomic profiling of the salt-stress response in the halophyte *Halogeton glomeratus*

**DOI:** 10.1186/s12864-015-1373-z

**Published:** 2015-03-11

**Authors:** Juncheng Wang, Baochun Li, Yaxiong Meng, Xiaole Ma, Yong Lai, Erjing Si, Ke Yang, Panrong Ren, Xunwu Shang, Huajun Wang

**Affiliations:** Gansu Provincial Key Lab of Aridland Crop Science/Gansu Key Lab of Crop Improvement & Germplasm Enhancement, Lanzhou, China; College of Agronomy, Gansu Agriculture University, Lanzhou, China; College of Life Sciences and Technology, Gansu Agricultural University, Lanzhou, China

**Keywords:** Halophyte, *Halogeton glomeratus*, Transcriptome, Illumina sequencing, Salt-stress

## Abstract

**Background:**

*Halogeton glomeratus* (*H. glomeratus*) is an extreme halophyte that is widely distributed in arid regions, including foothills, the Gobi desert of northwest China, and the marginal loess of Central Asia. However, research on the salt-tolerant mechanisms and genes of this species are limited because of a lack of genomic sequences. In the present study, the transcriptome of *H. glomeratus* was analyzed using next-generation sequencing technology to identify genes involved in salt tolerance and better understand mechanisms of salt response in the halophyte *H. glomeratus*.

**Results:**

Illumina RNA-sequencing was performed in five sequencing libraries that were prepared from samples treated with 200 mM NaCl for 6, 12, 24, and 72 h and a control sample to investigate changes in the *H. glomeratus* transcriptome in response to salt stress. The *de novo* assembly of five transcriptomes identified 50,267 transcripts. Among these transcripts, 31,496 (62.66%) were annotated, including 44 Gene Ontology (GO) terms and 128 Kyoto Encyclopedia of Genes and Genomes (KEGG) pathways. Compared with transcriptomes from the control and NaCl-treated samples, there were 2,223, 5,643, 7,510 and 10,908 genes that were differentially expressed after exposure to NaCl for 6, 12, 24, and 72 h, respectively. One hundred and eighteen salt-induced genes were common to at least two stages of salt stress, and 291 up-regulated genes were common to various stages of salt stress. Numerous genes that are related to ion transport, reactive oxygen species scavenging, energy metabolism, hormone-response pathways, and responses to biotic and abiotic stress appear to play a significant role in adaptation to salinity conditions in this species. The detection of expression patterns of 18 salt-induced genes by quantitative real-time polymerase chain reaction were basically consistent with their changes in transcript abundance determined by RNA sequencing.

**Conclusions:**

Our findings provide a genomic sequence resource for functional genetic assignments of an extreme halophyte, *H. glomeratus*. We believe that the transcriptome datasets will help elucidate the genetic basis of this species’ response to a salt environment and develop stress-tolerant crops based on favorable wild genetic resources.

**Electronic supplementary material:**

The online version of this article (doi:10.1186/s12864-015-1373-z) contains supplementary material, which is available to authorized users.

## Background

Salinity stress is one of the most serious factors that severely affect crop growth, development, and yield [1,2]. A better understanding of plant salt tolerance mechanisms is crucial for the sustainable development of agriculture worldwide [3]. Halophytes constitute approximately 1% of the world’s flora. They are adapted to highly saline soil conditions [2] and thus represent ideal materials to understand complex physiological and genetic mechanisms of salt tolerance [4]. Several halophytes have been investigated in physiological and molecular biological studies of the mechanisms of salt tolerance, such as *Thellungiella halophila* [3,5,6], *Crithmummaritimum* [7], *Suaeda fruticosa* [8], *Cakile maritime* [9], *Centaurea tuzgoluensis* [10], and *Spartina alterniflora Loisel* [11]. Especially *Thellungiella halophila*, it is a close relative of *Arabidopsis* with strong ability to adapt to saline environments, which make it suitable halophytic model for studying mechanisms of salt tolerance [5,6]. Genes may contribute to its salt-tolerance, such as related to cation transport, abscisic acid signaling, and wax production have been identified [3]. Adaption of salt tolerance in halophyte may involve a global network adjustment of multiple regulatory mechanisms [6,9,11]. However, unclear are whether all halophytes are similarly salt-tolerant and whether halophytes have specific salt-tolerant mechanisms [2]. Our understanding of the molecular basis of non-genomic halophytes is limited. The identification and isolation of novel genes using genomic approaches will advance our understanding of the salt-tolerance mechanisms of halophytes [12].

The halophyte *Halogeton glomeratus* (*H. glomeratus*) is a *chenopodiaceae* succulent annual herbaceous plant that is naturally distributed in arid and desert regions of northwest China, from Mongolia to Central Asia, and plays an important role in sustaining local ecosystems [13,14]. During the process of adaptation to drought, salinization, and desertification environments, *H. glomeratus* has evolved many strategies, including high tolerance to salt at the germination stage [15], stems that form multiple branches, increased leaf and stem succulents, the storage of large amounts of water, the compartmentalization of salinity in mesophyll cells, and specialized stomatal regulation to cope with environmental stress [14]. However, little effort has been made to elucidate the salt tolerance mechanisms of H. *glomeratus*. Previous studies have focused only on its morphological and physiological characteristics [13,15]. We recently reported that this species can grow normally under conditions of a NaCl concentration up to 500 mM, and a detailed analysis of the molecular mechanisms that underlie salt tolerance in *H. glomeratus* is required.

RNA transcript profiling is an important strategy for studying the expression of a large number of genes in a given tissue at a given time point [16]. RNA sequencing (RNA-Seq) technology is a major quantitative platform for transcriptome analysis that efficiently and economically enables investigations of transcriptomes in various gene expression studies, even for species that lack a reference genome [17,18]. This sequencing method has been widely applied to transcript profiling in numerous non-model species, and related candidate genes, single-nucleotide polymorphisms (SNPs), and simple-sequence repeats (SSRs) have been identified [19-21].

In the present study, paired-end sequencing technology was used to examine the leaf transcriptomes of *H. glomeratus* under salt stress conditions. We constructed five libraries that were sequenced using Illumina HiSeq 2000 and 2-month-old seedlings that were treated with 200 mM NaCl for 0, 6, 12, 24, and 72 h. We then analyzed the expression profiles of 50,267 identified unigenes during different stages of salt stress compared with the expression of unigenes in the control library (0 h). A total of 409 candidate genes were identified to be potentially related to the salt response and tolerance (118 salt-induced and 291 upregulated with salt stress at all stages). We validated salt-induced unigenes based on different stress stages using quantitative real-time polymerase chain reaction (qRT-PCR). This research will facilitate elucidation of the salt tolerance mechanisms of this species and provide an important wild gene resource to improve salt tolerance in crops through genetic manipulation.

## Results

### Na^+^ accumulation response to NaCl stress

**Figure 1:**
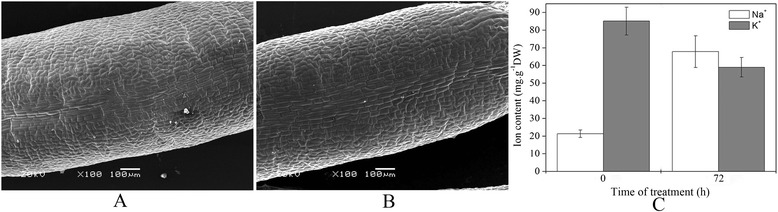
**Na** ^**+**^ **accumulation analyses of** ***H. glomeratus*** **seedlings after treated with NaCl.** Micrograph of leaf surfaces of *H. glomeratus* seedlings were treated with 200 mM NaCl for 0 h **(A)** and 72 h **(B)**. K^+^ and Na^+^ contents of leaves from seedlings were treated with 200 mM NaCl for 0 h and 72 h, respectively (n = 3) **(C)**. Vertical bars indicate standard deviation (SD).

After seedlings were treated with 200 mM NaCl for 72 h, the SEM study of leaf abaxial surfaces result showed that there was no salinity secreted to the leaf surface (Figure 1A and B). The K^+^ and Na^+^ contents significantly decreased and increased after 200 mM NaCl treated for 72 h, respectively (Figure 1C). Moreover, the Na^+^ concentrations in leaves treated with NaCl for 72 h was 3.18-fold higher than 0 h. Thus, we can affirm that a large amount of Na^+^ accumulated in leaf tissues in *H. glomeratus* under salt stress.

### *De novo* assembly and quantitative assessment of IIIumina sequence

To investigate the salt-induced global gene expression profile of the *H.glomeratus* transcriptome at different stages of salt stress, we constructed five cDNA libraries from leaves from seedlings treated with 200 mM NaCl for 0, 6, 12, 24, and 72 h. These libraries were sequenced on the Illumina Hiseq 2000 platform. A 23 gigabase (Gb) dataset was generated, and 297,955,888 raw reads were obtained. After removing low-quality regions, adapters, and all possible contamination, we obtained a total of 265,300,480 clean reads with Q20 > 97.66% and a GC percentage between 43.74% and 44.20% (Table 1). Each stage was represented by over 51 million high-quality reads, with numbers ranging from 51,344,528 to 54,646,950. The sequence output and quality statistics were adequate for use in the further analysis. All of the sequence data in this article have been deposited in the NCBI-SRA database and are accessible in SRS652178, SRS652189, SRS654343, SRS654344 and SRS654345, respectively.

**Table 1:** **Statistical summary of sequencing and assembly results**

	**0 h**	**6 h**	**12 h**	**24 h**	**72 h**
Total Raw Reads	60,099,376	59,434,584	59,981,694	58,988,346	59,451,888
Total Clean Reads	53,035,664	51,344,528	53,343,062	52,930,276	54,646,950
Total Clean Nucleotides (nt)	4,773,209,760	4,621,007,520	4,800,875,580	4,763,724,840	4,918,225,500
Q20 Percentage	97.68%	97.77%	97.70%	97.74%	97.66%
N Percentage	0.00%	0.00%	0.00%	0.00%	0.00%
GC Percentage	43.90%	44.20%	43.79%	43.74%	44.08%
Total Contigs	75,823	76,045	69,207	73,309	67,563
Total Length (nt)	32,435,990	30,098,496	29,746,317	31,507,438	30,173,681
Mean Length (nt)	428	396	430	430	447
N50	913	785	967	918	991
Total Unigenes	58,699	60,883	51,573	57,557	53,264
Total Length (nt)	33,330,719	29,510,487	29,170,625	31,846,285	29,670,894
Mean Length (nt)	568	485	566	553	557
N50	850	672	826	820	800
All Unigenes	Total Number: 50,267; Total Length (nt): 43,714,924; Mean Length (nt): 870; N50: 1441				

Note: The Q20 percentage is the proportion of nucleotides with a quality value > 20. The N percentage is the proportion of unknown nucleotides in clean reads. The GC percentage is the proportion of guanidine and cytosine nucleotides among total nucleotides. N50 is 50% of the assembled bases that were incorporated into sequences with a length of N50 or longer.

**Figure 2:**
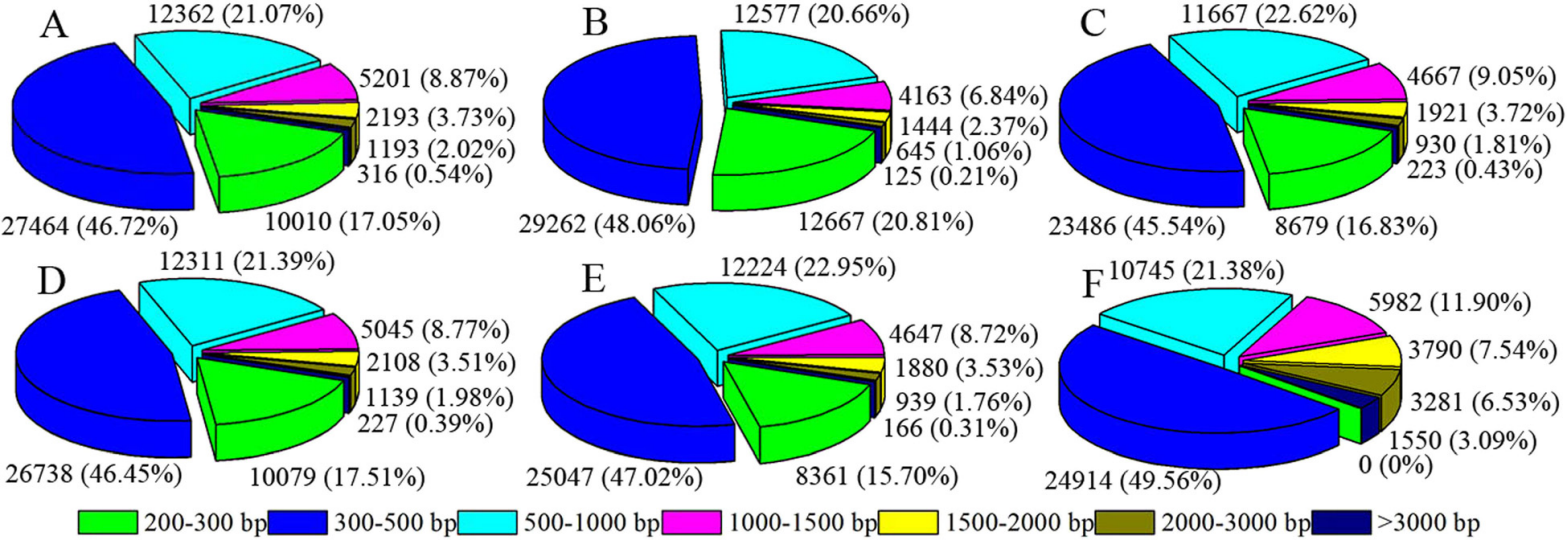
**Length distribution at 0, 6, 12, 24, and 72 h of salt stress and all unigenes. A-F** represent 0, 6, 12, 24, and 72 h and all unigenes, respectively. Values in colored square brackets indicate the range of unigene length. Unigene numbers and percentages (total number of unigenes in a certain length range) are shown next to the pie charts.

The transcriptome sequences were assembled using the Trinity program. A total of 50,267 sequences of the five libraries were assembled, with an average of 870 bp and N50 of 1441 bp. We generated 75,832 contigs for the control sample, with an average length of 428 nt. We generated 76,045, 69,207, 73,309, and 67,563 contigs for salt-stressed samples at 6, 12, 24, and 72 h with an average length of 396, 430, 430, and 447 nt, respectively. The average N50 length was longer than 900 nt in four libraries, with the exception of the library for salt-treated samples at 6 h (785 nt). After further clustering and assembly, we obtained a total of 58,699, 60,883, 51,573, 57,557, and 53,264 unigenes with an average length of 568, 485, 566, 553, and 577 nt and N50 length of 850, 672, 826, 820, and 800 nt, respectively. The quality of these assemblies and unigenes length distribution are shown in Table 1 and Figure 2.

### Sequence annotation

Several complementary approaches were utilized to annotate functional information about these assembled unigenes, including protein sequence similarities, clusters of orthologous groups (COG), gene ontology (GO), and Kyoto Encyclopedia of Genes and Genomes database (KEGG) pathway information. All of the unigenes were first compared with the NCBI Nr database, non-redundant nucleotide sequence (Nt) database, and the Swiss-Prot protein database by BLASTing with E values < 1e-5. Using the best hits found by BLAST, an inferred putative function was assigned to the sequences. The 29,588 (58.8%) unigenes were significantly matched to the known genes in Nr, a total of 22,810 (45.4%) unigenes were identified in Nt, and 19,117 (38.0%) sequences had best hits in the Swiss-Prot database.

**Figure 3:**
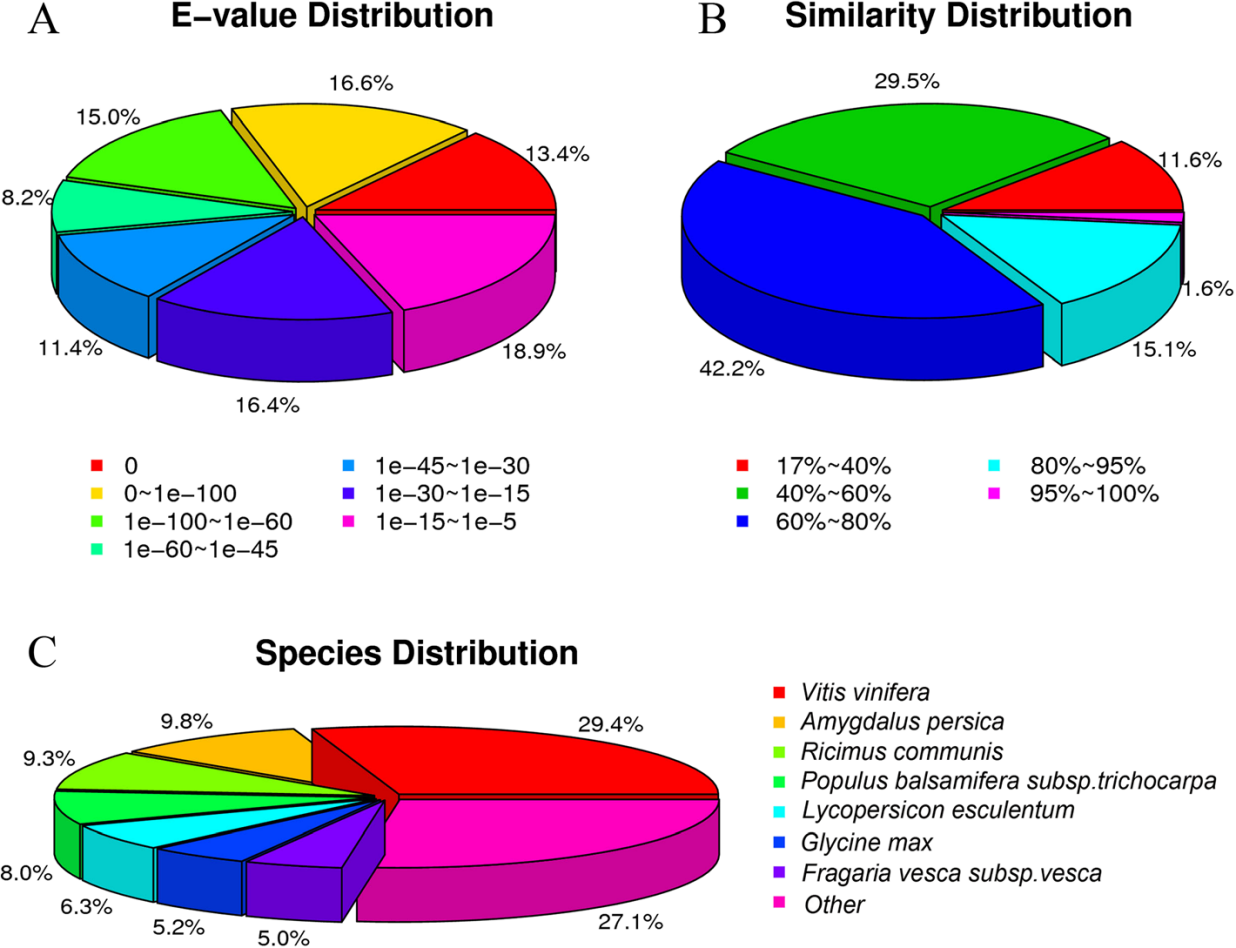
**Characteristics of similarity search of unigenes against Nr databases.** E-value distribution of Nr annotation results **(A)**. Similarity distribution of Nr annotation results **(B)**. Species distribution of Nr annotation results **(C)**.

The E-value distribution of the top hits in the Nr database showed that 43.21% of the sequences were mapped to the known genes in plants with best hits (E value < 1e-45, mean identity = 53.21%), and approximately 17.11% of the unigenes hit deposited sequences with similarity > 80% (Figure 3A and B). Approximately 72.86% of the annotated unigenes could be assigned with a best score to the sequences from the top seven species: *Vitis vinifera* (29.39%), *Amygdalus persica* (9.80%), *Ricimus communis* (9.30%), *Populus balsamifera subsp. trichocarpa* (7.95%), *Lycopersicon esculentum* (6.27%), *Glycine max* (5.16%), and *Fragaria vesca subsp. vesca* (4.99%; Figure 3C).

**Figure 4:**
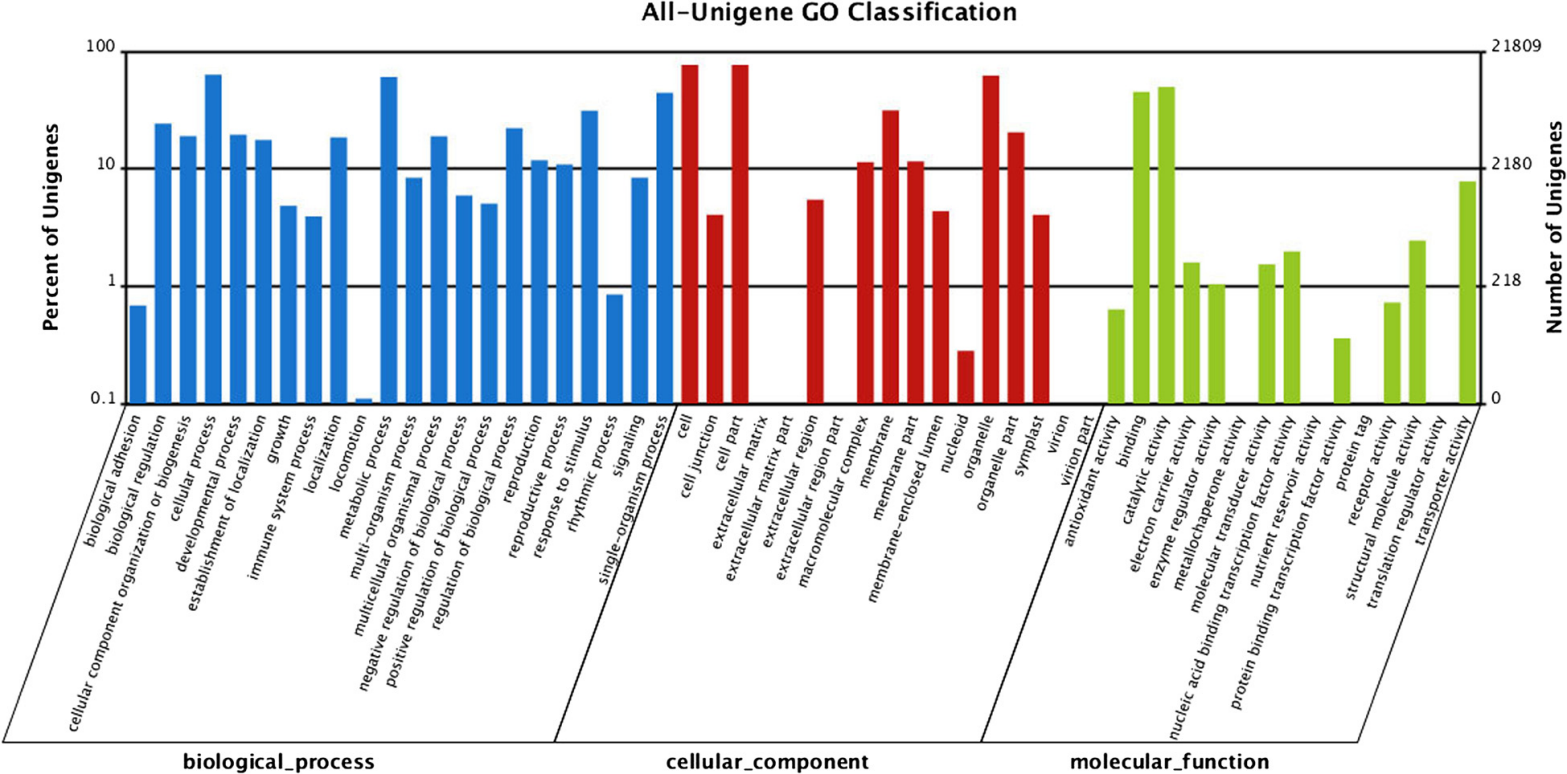
**Histogram presentation of all unigenes’ Gene Ontology classification.** The results are summarized in three main categories (biological process, cellular component, and molecular function) and 44 subcategories. The left Y-axis represents the percentage of a specific category of genes in each main category, and the right Y-axis represents the number of genes in a category.

For functional categories of 50,267 unigenes, a total of 21,809 unigenes were assigned at least one GO term to describe biological processes, molecular functions, and cellular components. Interestingly, cellular processes, metabolic processes, single organisms, and response to stimulus were significantly overrepresented in the 22 biological process GO groups (Figure 4, Additional file 1: Table S2).

To further predict the genes with different expression levels under salt-stressed conditions, the GO functional analysis was performed on the differentially expressed genes. The differentially expressed genes (DEGs) were defined as those with an FDR ≤ 0.001 and log2 ratio ≥ 1 (RPKMs > 2).

**Figure 5:**
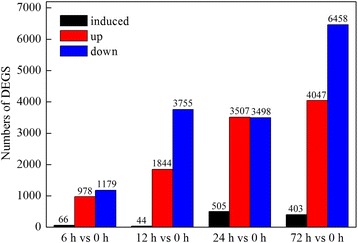
**Statistical chart of DEGs of transcriptomes in response to salt stress.** Compared with the transcriptional level at 0 h, 66, 44, 505, and 403 genes induced expression, 978, 1,844, 3,507, and 4,047 genes were upregulated, and 1,179, 3,755, 3,498, and 6,458 genes were downregulated, respectively.

The DEGs were analyzed by comparing the 6, 12, 24, and 72 h libraries with the control library. The results showed that more DEGs were detected along with extending the NaCl exposure time, and the number of DEGs at the later stage of stress (72 h) was much more than at early stages (6, 12, and 24 h; Figure 5). All of the DEGs was categorized into 49 functional groups (Additional file 2: Figure S1). Although seedlings were treated with NaCl for different times, the overall categorization of the genes in these four stages was very similar. Of these, in biological processes, the dominant terms were “cellular process”, “metabolic process”, “single-organism process”, and “response to stimulus”. In the cellular component, the dominant terms were “cell”, “cell part”, “organelle”, and “membrane”. In molecular function, the dominant terms were “binding”, “catalytic activity”, “structure molecule activity”, and “transporter activity”.

**Figure 6:**
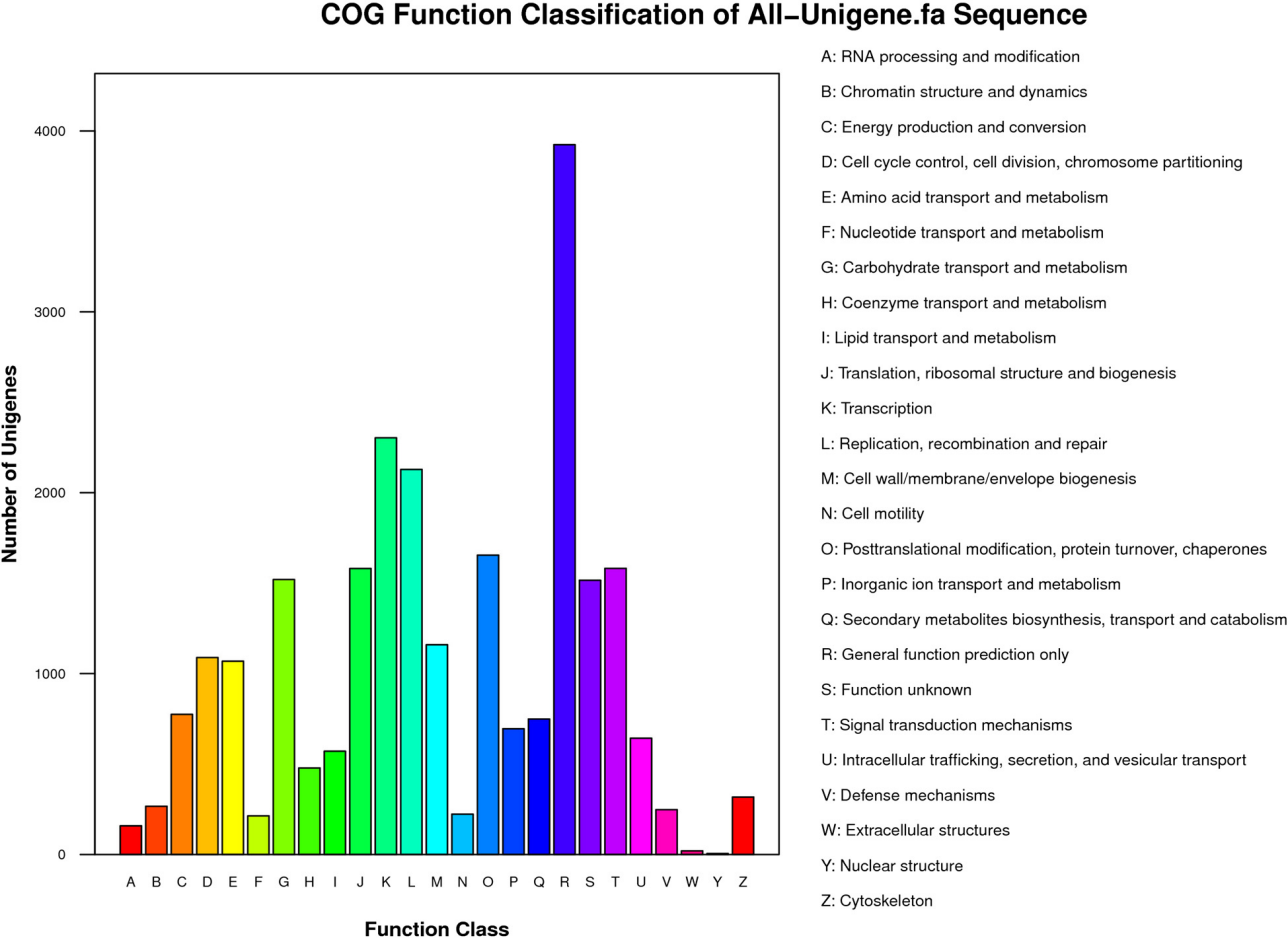
**Histogram presentation of COG classification.** Of 29,558 Nr hits, 11,728 sequences had COG classifications and were functionally classified into 25 categories. The y-axis indicates the number of unigenes in a specific functional category.

Unigenes were also aligned to the COG database to predict and classify possible functions. A total of 11,728 unigenes were distributed into 25 COG categories (Figure 6, Additional file 1: Table S2) among which the COG category “General function prediction only” represented the largest group (3,924; 15.76%), followed by “Transcription” (2,304; 9.25%), “Replication, recombination and repair” (2,129; 8.55%), “Posttranslational modification, protein turnover, chaperones” (1,656; 6.65%), “Signal transduction mechanisms” (1,583; 6.36%), and “Translation, ribosomal structure and biogenesis” (1,582; 6.35%). The smallest groups were “RNA processing and modification” (158; 0.64%), “Extracellular structures” (20; 0.08%), and “Nuclear structure” (5; 0.02%).

We obtained KEGG pathway annotation for 29,558 unigenes. A total of 17,410 sequences were assigned to 128 pathways. “Metabolic pathways (ko01100)” represented the largest group (4,422; 25.50%), followed by “Biosynthesis of secondary metabolites (ko01110)” (2,160; 12.41%), “Plant-pathogen interaction (ko04626)” (915; 5.26%), “Plant hormone signal transduction (ko04075)” (781; 4.48%), and “RNA transport (ko03013)” (732; 4.20%; Additional file 3: Table S3). Compared with the control, a total of 17,410 differentially expressed unigenes were assigned to 115, 126, 124, and 126 KEGG pathways, respectively (Additional file 4: Table S4, Additional file 5: Table S5, Additional file 6: Table S6 and Additional file 7: Table S7). After multiple-testing corrections, we chose pathways with Q values ≤ 0.05 as significantly enriched among the DEGs. A total of 27, 39, 31, and 44 significant enrichment pathways were detected in the salt-treated samples at 6, 12, 24, and 72 h, respectively. The enrichment pathways with more genes than the other pathways were “Metabolic pathways (ko01100)”, “Biosynthesis of secondary metabolites (ko01110)”, “Endocytosis (ko04144)”, “Ether lipid metabolism (ko00565)”, “Glycerophospholipid metabolism (ko00564)”, “Plant hormone signal transduction (ko04075)”, “Plant-pathogen interaction (ko04626)”, and “Starch and sucrose metabolism (ko00500)”. These annotations are a valuable resource for comparisons of processes, functions, and pathways in salt-tolerant *H. glomeratus* research. Additionally, 29,245 CDS mapped to the protein database. The number of predicted CDS was 2,209, and the total number of CDS was 31,454 (Additional file 8: Figure S2).

### Detection of salt-induced genes related to salt tolerance

**Figure 7:**
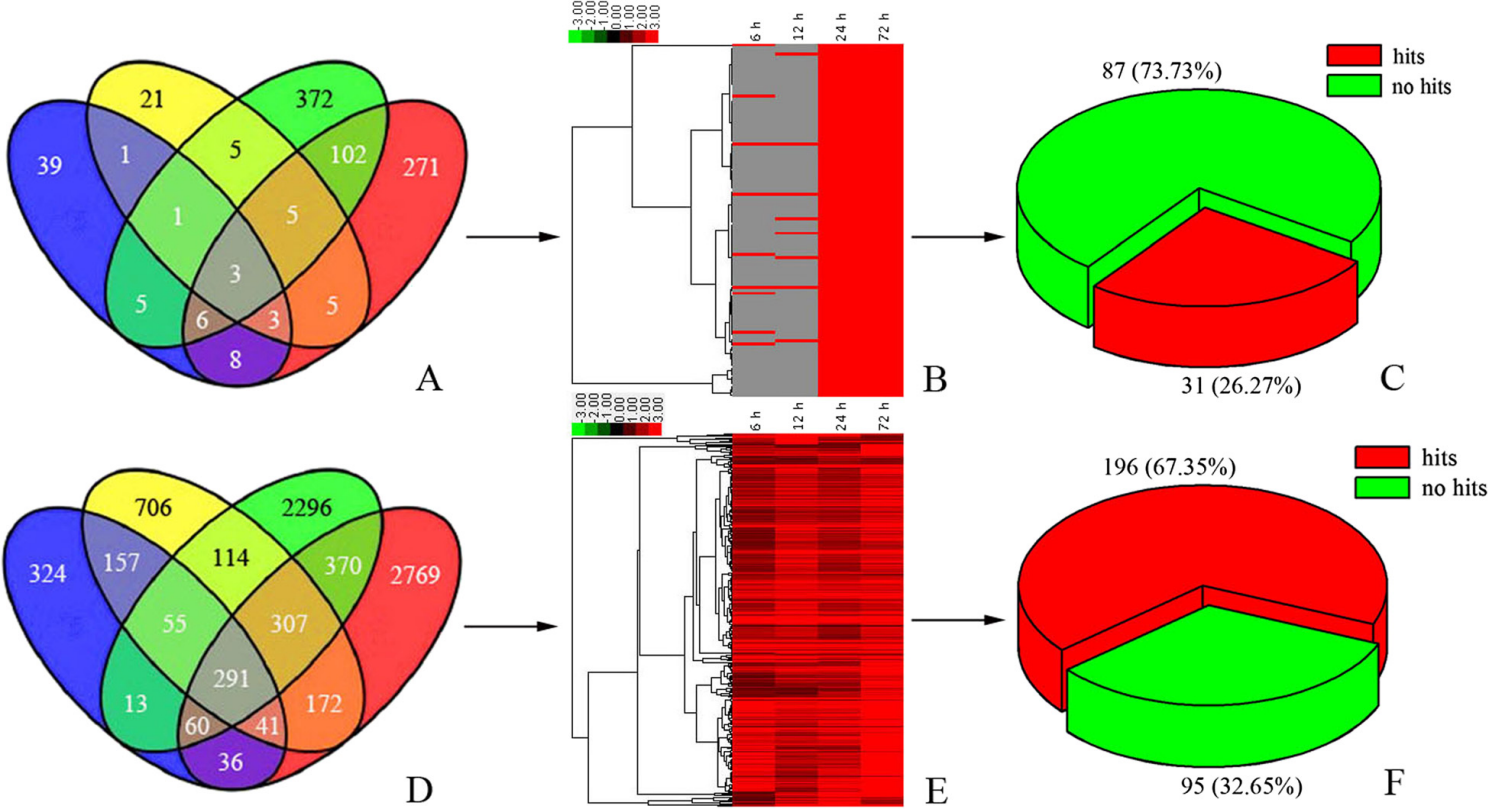
**Comparison of four transcriptomes for classification of DEGs and statistics of sequence annotation of DEGs.** Venn diagram analysis of salt-induced **(A)** and upregulated **(D)** unigenes. Unigenes identified by control and salt-stressed samples at 6 h (blue), 12 h (yellow), 24 h (green), and 72 h (red). Hierarchical cluster analysis of salt-induced **(B)** and upregulated **(E)** unigenes under salinity stress compared with the control. The rows represent individual unigene. The not detected, up-regulated unigenes are indicated in gray and red, respectively. Statistical chart of sequence annotation of salt-induced **(C)** and upregulated **(F)** unigenes. Sequence showed homology with known sequences were classified as “ hits”, and sequence showed no homology with known sequences were classified as “no hits”.

A rigorous algorithm (FDR ≤ 0.001, log2 ratio ≥ 1) of the RPKM derived read counts was performed to identify DEGs between control and salt-treated samples. At 6 h, 978 genes were upregulated, 66 were uniquely expressed under salt treatment, and 1,179 were downregulated. At 12 h, 1,844 were upregulated, 44 were uniquely expressed under salt treatment, and 3,755 were downregulated. At 24 h, 3,507 were upregulated, 505 were uniquely expressed under salt treatment, and 3,498 were downregulated. At 72 h, 4,047 were upregulated, 403 were uniquely expressed under salt treatment, and 6,458 were downregulated (Figure 5). A total of 118 salt-induced genes were common to at least two stages of salt stress (Figure 7A and Additional file 9: Table S8, Additional file 10: Table S9), and 291 up-regulated genes were common to various stages of salt stress (Figure 7D and Additional file 11: Table S10). During all stages of NaCl stress, the number of upregulated genes exceeded the number of genes induced by salt. Furthermore, to profile changes in the gene expression patterns of 118 salt-induced and 291 up-regulated genes in response to salinity stress, the hierarchical cluster was used to analysis their expression patterns during each stage. Here, the salt-induced and upregulated genes were divided into three main clusters, respectively (Figure 7B, E).The sequence analysis showed that of the 118 salt-induced unigenes, 87 (73.72%) were found to have known functions (Figure 7C). Of the 291 upregulated unigenes, 96 (32.65 %) showed no homology with known sequences and were classified as “no hits” (Figure 7F). We identified a number of unigenes that were induced by salt treatment and expressed in enrichment pathways, such as the adenosine triphosphate (ATP)-citrate synthase α chain protein 3 (Unigene5334), vegetative cell wall protein (Unigene7663), *Solanum lycopersicum* serine hydroxymethyltransferase (Unigene2603), disease resistance protein (CL5609.Contig1), peroxidase (CL5820.Contig1), and vacuolar proton ATPase (Unigene8306; Additional file 9: Table S8).

### Verification of differential gene expression by qRT-PCR

**Figure 8:**
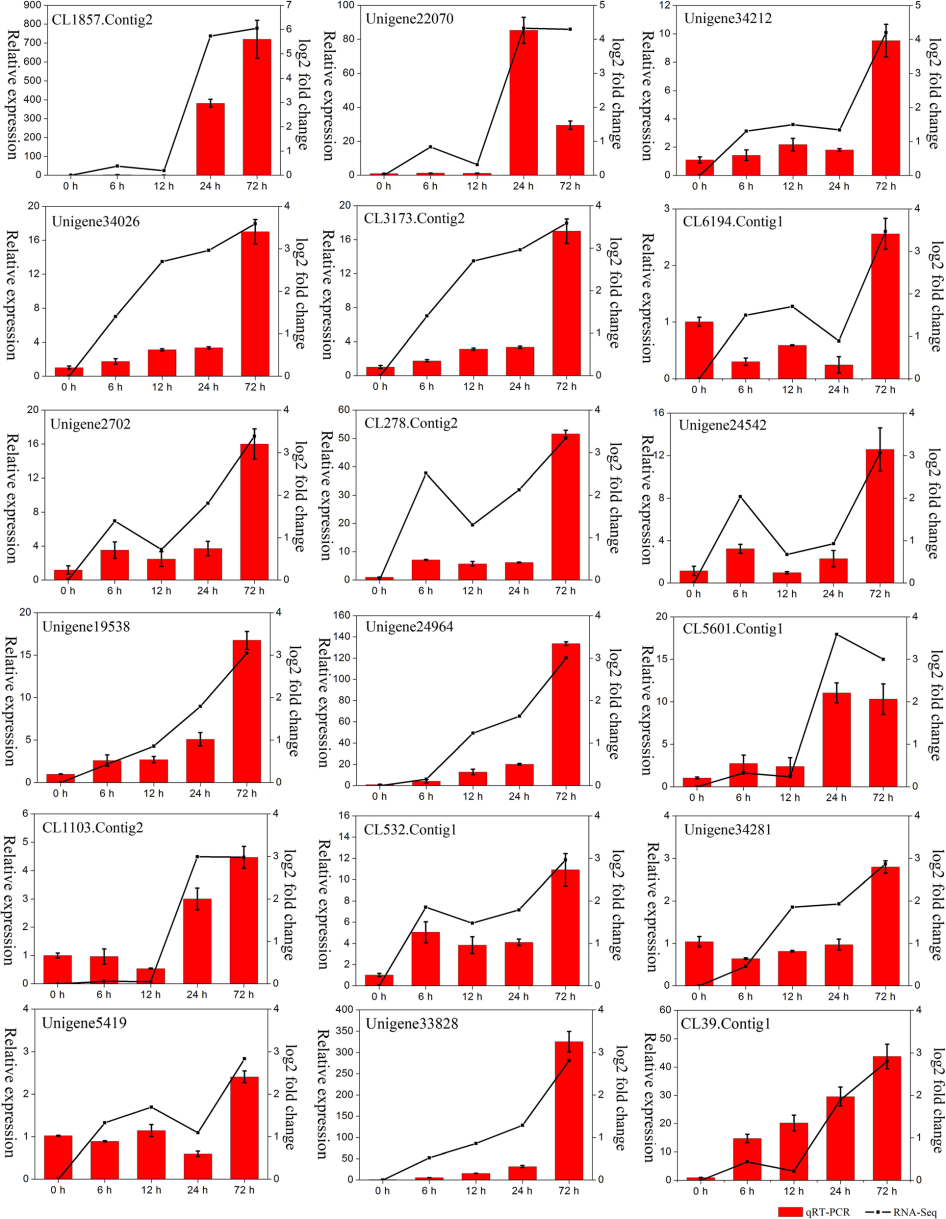
**qRT-PCR analyses of 18 salt-induced unigenes during various stages of salt stress.** Expression pattern of selected genes was analyzed at 0, 6, 12, 24, and 72 h. Red bars with standard errors represent the relative expression level determined by qPCR using the 2^-ΔΔCT^ method from three independent biological replicates (left Y-axis). Broken lines indicate transcript abundance changes (log2 fold) based on RPKM values according to RNA-Seq (right Y-axis).

To further validate the results from the Illumina sequencing data, 18 salt-induced unigenes were selected for qRT-PCR analysis of samples that were treated with 200 mM NaCl for 0, 6, 12, 24, and 72 h. In the five stages of stress, the expression trends of the unigenes from the qRT-PCR and RNA sequencing analyses were largely consistent (Figure 8). These results demonstrate that the transcriptomic profiling data accurately reflected the response of *H. glomeratus* to salt stress.

### SSR and SNP identification

For further application of *H. glomeratus*, SSRs and SNPs were discovered using the assembled transcriptomes. A total of 23,876 SSRs was detected in transcriptomes, and the major types of the identified SSRs were mono-nucleotide (3,378), di-nucleotide (1,962), tri-nucleotide (5,489), quad-nucleotide (397), penta-nucleotide (418), and hexa-nucleotide (511). The most frequent SSR motif was AT/TA (350), followed by TA/AT (328), AG (282), GAA (292), TC (272), and TGA (272). We identified a total of 172,064 SNPs between transcriptomes, among which 61,785 were transitions, and 110,279 were transversions (Additional file 12: Figure S3). The SSRs and SNPs identified in this study provided a valuable resource for future studies on genetic linkage mapping and the analysis of interesting traits in *H. glomeratus*.

## Discussion

Numerous physiological [22,23], functional genomics [3,24], and proteomics [25] studies have identified physiological and molecular mechanisms of plant salt tolerance. However, our current understanding of the complex mechanisms of salt tolerance in plants remains limited. Recently, next-generation sequencing technology has provided a powerful tool for transcriptome analysis, and the *de novo* assembly of transcript sequences offers a rapid approach to obtain expressed gene catalogs for non-model organisms [26]. In the present study, we performed deep sequencing analysis of *H. glomeratus* in response to salt stress using the Illumina HiSeq 2000 platform and *de novo* transcriptome assembly. We identified 50,265 unigenes with an average length of 870 bp and N50 length of 1,441 bp using blast against the Nr, Nt, Swiss-Prot, GO, COG, and KEGG databases. We identified 31,486 (62.64%) unigene hits with known plant species, and the remaining 18,779 (37.36%) failed to hit any homologs, which might be considered novel genes. Gene function and expression studies are needed to further investigate the genes that are involved in the process of salt tolerance in *H. glomeratus*.

In the present study, the top seven species (Figure 3C) were the most highly related species with known genomes to *H. glomeratus*, and 21,534 (72.86%) annotated unigenes in *H. glomeratus* hit protein sequences in these species. However, none of these species belong to *chenopodiaceae* plants, which is one of the main generas of halophytes or xerophytes [27], and none of these species belong to herbage halophytes. There were 21,809 unigenes with GO annotation and 17,410 unigenes mapped to 128 pathways with KEGG annotation (Additional file 3: Table S3, Additional file 4: Table S4, Additional file 5: Table S5, Additional file 6: Table S6 and Additional file 7: Table S7; Additional files 13, 14, 15, 16 and 17). The deep sequencing and exhaustive annotation results will improve genome annotation and facilitate the exploitation of genetic resources that are responsible for salt tolerance in the halophyte *H. glomeratus*.

The high tolerance of *H. glomeratus* to salinity enabled us to identity a succession of gene expression changes and trace the underlying salt-responsive genes under different salt treatment conditions [14]. The enriched dominant GO terms that were identified during salt stress included “cellular process”, “metabolic process”, “single-organism process”, and “response to stimulus”, which are mostly in agreement with other analyses of DEGs under salt stress conditions [20,28]. However, the enriched term “single-organism process” was firstly reported as one of the enriched dominant GO terms under salt stress conditions, suggesting that *H. glomeratus* may have special genes that regulate the salt-response.

To identify significant gene expression changes with NaCl treatment, differentially expressed unigenes were analyzed by comparing the 6, 12, 24, and 72 h libraries with the control library. We focused on the unigenes that were strongly induced by NaCl or upregulated after salt stress. In the present study, a large number of unigenes were identified, thus clarifying the mechanisms of salt tolerance and identifying candidate genes that are responsible for salt adaptation in *H. glomeratus*. Of the candidate genes, 118 salt-induced genes and 291 up-regulated genes under salt-treatment conditions were analyzed to elucidate salt adaptation strategies. However, there were only a few genes know its function of these genes, a total of 31 (26.27%) and 196 (67.35%) were successfully identified in the salt-induced and up-regulated genes, respectively (Additional file 9: Table S8, Additional file 10: Table S9 and Additional file 11: Table S10). It is necessary to verify whether these genes of unknown function play important roles in response to salt stress in the further study.

The mechanisms of salinity tolerance involve three strategies, including tolerance to osmotic stress, Na^+^ exclusion from leaf blades, and tissues tolerance [29]. The tolerance of tissue mainly occurs through the compartmentalization of Na^+^ and Cl^−^ in vacuoles in mesophyll cells in the leaf to avoid toxic concentrations within the cytoplasm, and this process is implemented by transporting ions both through plasma and across tonoplasts [30]. *H. glomeratus* is a leafy, highly succulent halophyte. It does not have salt glands or salt bladders in the leaves and stores Na^+^ in leaves under salinity conditions (Figure 1). In the present study, V-ATPase (V-type H^+^-transporting ATPase; Unigene8306 and CL4328.Contig1) transcripts were present at high levels in the salt-stress libraries. V-ATPase is essential for establishing an electrochemical H^+^-gradient across tonoplasts to energize tonoplasts for efficient ion uptake into vacuoles via the tonoplast Na^+^/H^+^ antiporter (*NHX*) [31]. Transcript profiling of the salt-tolerant *Festuca rubra ssp. litoralis* revealed that V-ATPase mediated ion transport under salinity stress conditions, and enhanced V-ATPase activity can contribute to improvements in salt tolerance in transgenic crops [32,33]. Our results showed that salt-induced transcript of the V-ATPase was enhanced by prolonging the salt treatment time. However, vacuolar Na^+^/H^+^ antiporters in leaves are mainly involved in compartmentalizing Na^+^ in vacuoles. In contrast to our expectation, this gene was not shown significantly up-regulated under typical salinity condition. Similarly, we did not find Na^+^/H^+^ antiporters exhibited increased abundance at the levels of protein in response to salt stress [14]. Few reports have indicated that vacuolar Na^+^/H^+^ antiporters and V-ATPase play a coordinated role in sequestering Na^+^ in vacuoles in the process of the salt stress response in plants [33,34]. Apse reported that the overexpression of vacuolar Na^+^/H^+^ antiporters in *Arabidopsis thaliana* enhanced salt tolerance and the vacuolar antiporter activity of transgenic plants but did not detect an increase in *AtNHX1* transcript levels in response to NaCl stress at 50-250 mM [35]. This is consistent with our results. Perhaps the increase in vacuolar Na^+^/H^+^ antiporter activity in response to NaCl stress in *H. glomeratus* requires a higher concentration of NaCl. Our transcriptome sequencing revealed that the higher transcript levels of V-ATPase contributes to providing more energy and forming a stronger proton gradient to drive excessive cytoplasmic Na^+^ into vacuoles. Therefore, a reasonable assumption is that *H. glomeratus* has tighter control over ion compartmentalization by adjusting the driving force of ion transport.

The exposure of plants to abiotic stress, such as high salinity, drought, and low temperatures, causes a dramatic increase in reactive oxygen species (ROS) production [4], including superoxide radicals (O^-2^), hydrogen peroxide (H_2_O_2_), and hydroxyl radicals (^-^OH), which can perturb cellular redox homeostasis and result in oxidative damage to cellular structures and eventually lead to cell death [36]. A large number of unigenes that are associated with in ROS scavenging-related genes were identified, including glutathione *S*-transferases (GSTs; Unigene6753), which have glutathione peroxidase (GPX) activity and can use glutathione (GSH) to prevent the degradation of organic hydroperoxides to cytotoxic aldehyde derivatives. The activity of GSTs is upregulated in salt-stressed plants [37], and the overexpression of GST activity improves abiotic stress tolerance in plants [38]. Peroxidases (PODs; CL5820.Contig1), which are also important in H_2_O_2_ scavenging, exhibit increased levels in salt-stressed seedlings, consistent with the salt-treated halophyte *Aeluropus littoralis* [39]. Serine hydroxymethyltransferase (SHMT; Unigene2603 and Unigene1010) are involved in the photorespiratory pathway and plays an important role in plants to minimize the production of ROS in chloroplasts and mitigate oxidative damage [40]. Therefore, antioxidant enzymes that are involved in the ROS scavenging pathway protect cells from oxidative damage under salt stress conditions in *H. glomeratus*.

The adjustment of energy metabolism under salt stress conditions is an important strategy to cope with salinity stress [36]. We identified three unigenes that were related to energy metabolism. One (Unigene5334) was an homolog to ATP-citrate synthase, the expression of which was induced under salt stress conditions. Two unigenes were homologs to ATP synthase (CL5295.Contig2), adenosine diphosphate (ADP), and ATP carrier protein (Unigene6293) and upregulated under salt stress conditions. One unigene (CL5099.Contig1) was found to be homologous to cytochrome c oxidase, which is an electrostatically coupled energy transducer that contributes to the formation of ATP in aerobic life [41]. Efficient ATP generation is crucial for salt tolerance during ion exclusion and tissue tolerance in plants.

Plants integrate multiple hormone-response pathways for adaptation to environmental stress, including the abscisic acid (ABA), cytokinin, auxin, and strigolactone pathways [42]. Abscisic acid signaling regulation enables plants to survive under environmental stress conditions [43]. In the present study, we found that numerous upregulated unigenes were related to the hormone-response pathway, such as abscisic stress-ripening protein (ASR; Unigene3863) in the ABA signaling regulation pathway. ASR is transiently upregulated when plants are exposed to salt or water stress [44]. Cytokinins (CKs) regulate several plant growth and developmental processes, such as cell division, leaf senescence, and nutritional signaling. We identified a CK hydroxylase (Unigene9103) that is involved in the regulation of CK metabolism and signaling of *Arabidopsis* [45]. Calreticulin (CR; Unigene6139) was upregulated under salt stress conditions and is essential for integrin-mediated calcium signaling and cell adhesion [46]. Additionally, seven unigenes (Unigene1081, Unigene3770, Unigene4073, Unigene4177, Unigene6237, Unigene6742, and Unigene8963) were strongly upregulated in response to salt stress and found to be homologs of the proline-rich receptor-like protein kinase (PERK) family in *Arabidopsis*, which plays an important role in hormone signal transduction for plant growth and development, stress responses, and disease resistance [47].

We also found numerous unigenes that are involved in the response to biotic and abiotic stress. The expression of cysteine proteinase (Unigene6468) is strongly induced in leaves in response to salinity and drought stress, and it is also involved in signaling pathways and the response to biotic and abiotic stress [48,49]. Aspartate aminotransferase (AST) catalyzes the bidirectional conversion of aspartate into glutamate, and its activity significantly increased under salt shock [50]. Recent reports showed that this gene was involved in amino acid metabolism, which might interact with plant defense responses [51]. The transcript (Unigene4102) was identified as betaine aldehyde dehydrogenase (BADH), a key enzyme that catalyzes the final reaction in the glycine betaine synthetic pathway of the plant response to drought and salt stress [52], thus helping maintain osmotic homeostasis in response to water deficit, osmotic stress, and salt stress.

Aquaporins play an important role in regulating transmembrane water transport during plant growth and development and under osmotic stress and salinity stress conditions [53]. The overexpression of aquaporins was recently reported to increase the salt stress tolerance of transgenic tobacco [54].

A number of unigenes were identified as vegetative cell wall proteins (Unigene7663, Unigene3984, Unigene4128, Unigene6186, Unigene6746). These proteins have many functions, including structural functions, and participate in plant morphogenesis [55]. Notably, the transcripts of vegetative cell wall proteins that changed under salinity conditions are involved in cell adaptations to salt, but our understanding of the nature of these changes is still limited.

Finally, we also focused on some genes that are involved in the photosynthetic pathway. In the present study, two genes that encode key enzymes of carbon assimilation in the C_4_ photosynthetic pathway were present in the transcriptomic dataset from the KEGG annotation (ko00170), including phosphoenolpyruvate carboxylase (PEPC; Unigene338) and NADP-malate dehydrogenase (NADP-MDH; Unigene4014). Two genes that encode major C_4_ decarboxylation enzymes were also found in the transcriptomic KEGG dataset (ko00620), including NADP-malic enzyme (NADP-ME; Unigene216, Unigene6517, and CL5690.contig1/2) and NAD-malic enzyme (NAD-ME; CL6152.contig1/2). This is consistent with our previous anatomical research on *H. glomeratus*. The anatomical structure analysis of leaves showed that the palisade tissue, bundle sheath tissue, and vascular bundle form the kranz anatomy as a typical feature the C_4_ photosynthetic pathway [14]. No studies of which we are aware have reported the photosynthetic pathway of *H. glomeratus*. C_4_ plants have many advantages over C_3_ plants in photosynthetic performance, nitrogen and water use efficiency, and the response to environmental stress [56]. The presence of C_4_ enzymes in leaves is the first transcriptomic evidence to support the existence of C_4_ pathways in the *H. glomeratus* response to environmental stress.

## Conclusions

In the present study, we surveyed five transcriptomes of *H. glomeratus* leaves during various stages of salt stress, and 50,267 unigenes were generated with the Illumina HiSeq 2000 platform. We identified 118 salt-induced genes and 291 upregulated unigenes that are involved in ion transport, ROS scavenging, energy metabolism, hormone-response pathways, and response to biotic and abiotic stress in the response of this species to salt stress. Our findings substantially enrich the existing sequence resources of *H. glomeratus* and will greatly promote research on salt-tolerant mechanisms of halophytes. This work also provides numerous salt-tolerant candidate genes for further functional analyses to improve the salt tolerance of crops.

## Methods

### Plant material

Seeds of *H. glomeratus* were collected in a salinized river shoal in Huining County, Gansu Province, in Northwest China and planted in plastic pots that were filled with a mixture of sand and vermiculite (1:1, v/v). The plants were placed under long-day (16 h light/8 h dark cycle) conditions at a temperatures of 25°C (light) and 18°C (dark) with irradiation intensity of approximately 300 μmol m^−2^ s^−1^. The plants were irrigated daily with half-strength Hoagland solution. Two months after germination, seedlings were treated with 200 mM NaCl for 6, 12, 24, and 72 h. Untreated seedlings were used to construct a reference library for *de novo* sequencing. Leaves were immediately harvested and frozen in liquid nitrogen for the extraction of total RNA.

### Na^+^ accumulation analyses of seedlings after treated with NaCl

For salt crystals of leaf surfaces assays, the leaf abaxial surfaces of seedlings treated with 200 mM NaCl for 72 h were scanned with an scanning electron microscopy (SEM) (JSM-5600LV, JEOL Ltd., Japan) with 20.0 kV current. For Ion concentration determination of the leaves, leaf samples harvested from the seedlings were treated with 200 mM NaCl for 0 h and 72 h. Three biologically independent replicates were prepared. Then, K^+^ and Na^+^ contents were measured using an atomic absorption spectrophotometer (AA240; Varian Medical Systems, USA).

### RNA extraction and quality determination

Total RNA was extracted using TRIzol reagent (Invitrogen, Carlsbad, CA, USA) according to the manufacture’s protocol. The RNA samples were digested using DNase I at 37°C for 30 min to remove potential genomic DNA contamination. The concentration and quality of each sample were determined using an Agilent 2100 bioanalyzer. High-quality RNA from three replicates at each time point at equal quantities were pooled for cDNA preparation and RNA-Seq.

### IIIumina cDNA library preparation and sequencing

Poly (A) mRNA was purified from total RNA using magnetic beads with oligo (dT). The mRNA was then broken into short fragments using divalent cations at an elevated temperature. Using these cleaved RNA fragments as templates, random hexamer primer was used for first-strand cDNA synthesis, followed by second-strand cDNA synthesis using DNA polymerase I and RNase H. These cDNA fragments were subjected to purification, end repair, and ligation to sequencing adapters. The products were then amplified by PCR to construct the cDNA library.

### Sequencing and de novo assembly

Sequencing libraries were sequenced using the Illumina HiSeq2000 system at the Beijing Genomics Institute (Shenzhen, China). Raw sequencing image data were transformed by base calling into raw reads. The raw reads were cleaned by discarding adaptor sequences and reads with unknown nucleotides that were larger than 5% and low-quality reads. The rate of reads with quality ≤ 10 was > 20%. The subsequent analysis was based on these clean, high-quality reads. The transcriptome *de novo* assembly of clean reads was performed using the short-read assembling program Trinity [57]. Short reads with overlapping sequences were first assembled to form the longest sequences, referred to as contigs. These contigs were then pooled to build de Brujin graphs. Contigs from the same transcript and the distances between these contigs were detected based on the paired-end read. Finally, sequences that lacked Ns and could not be extended on either end were defined as unigenes. Unigenes from each sample’s assembly were taken for further processing, including sequence splicing and redundancy removal using sequence clustering software to acquire non-redundant unigenes that were as long as possible. After clustering, the unigenes were divided into clusters (CL prefix) and singletons (Unigene prefix).

### Functional annotation

The expression profile of each unigene was calculated using the RPKM method (reads per kb per million reads) and normalized using ERANGE 3.1 software. If more than one transcript was identified for a given gene, then the longest transcript was used to calculate its expression level and coverage. The false discovery rate (FDR) method was used to determine the threshold of the *p* value in multiple tests. Unigene annotations provided functional annotations for all of the unigenes, together with their expression levels. All of the generated unigene sequences were aligned by BLASTX (E value < 0.0001) to public proteins databases, such as the National Center for Biotechnology Information non-redundant protein (Nr) database, Swiss-Prot, Kyoto Encyclopedia of Genes and Genomes (KEGG) database, and clusters of orthologous groups (COG) database, with an E cutoff value of 1e-5. The best-aligning results were used to determine the sequence direction of the unigenes. If the results from different databases conflicted with each other, then the following priority order was followed when deciding the sequence orientation of the unigenes: NCBI nr, Swiss-Prot, KEGG, and COG.

With Nr annotation, we used the Blast2GO program (https://www.blast2go.com/blast2go-pro; accessed July 12, 2014) to obtain the annotation of unigenes with GO terms. We then used WEGO software (http://wego.genomics.org.cn; accessed July 12, 2014) for GO functional classification for all of the unigenes and determine the distribution of gene functions of the species at the macro level. KEGG pathway annotation was performed using blastall software against the KEGG database (http://www.genome.jp/kegg/; accessed July 12, 2014).

### Prediction of unigene coding regions

For coding region prediction (CDS) of the unigenes, all of the unigenes were first aligned by blastx (E value < 0.00001) to protein databases in the priority order NCBI nr, Swiss-Prot, KEGG, and COG. Unigenes that were aligned to a higher-priority database did not align to a lower-priority database. Proteins with the highest ranks in the BLAST results were taken to determine the CDS of the unigenes, and the CDS was translated into amino acid sequences with the standard codon table. Both the nucleotide sequences (5′ → 3′) and amino acid sequences of the unigene coding region were acquired. When a unigene happened to be unaligned to none of the above databases, then ESTScan software was introduced to decide its sequence orientation and protein coding region prediction (CDS) [58].

### Identification of different expressed genes, SSRs, and SNPs

To identify genes that are regulated by salt stress, the significance of differences in gene expression between salt-treated and control samples was determined using a threshold FDR ≤ 0.001 and an absolute log2 ratio ≥ 1 (at least two-fold change). The genes that were expressed at different levels across samples were further annotated by GO enrichment analysis and KEGG pathway enrichment analysis to ascertain biological significance. The self-organizing tree algorithm of hierarchical clustering of gene express level profiles was performed on the log (RPKM) values of genes using Cluster software (version 3.0; http://rana.lbl.gov/EisenSoftware).

The SSR detection of unigenes was preformed with MicroSAtellite (MISA) software (http://pgrc.ipk-gatersleben.de/misa/misa.html; accessed July 12, 2014). Primers were designed using Primer 3 software (http://primer3.sourceforge.net; accessed July 12, 2014). We used SOAPsnp (http://soap.genomics.org.cn/soapsnp.html; accessed July 12, 2014) to identify SNPs in the transcripts of *H. glomeratus.* The SNPs could then be identified based on the consensus sequence by comparisons with the unigenes.

### Gene validation and express analysis

Samples were prepared, and total RNA was isolated using the same method mentioned above. The salt-induced expression of unigenes with potential roles in the salt stress response were chosen for validation using qRT-PCR. A total 18 unigenes were selected for qRT-PCR with RPKM values > 5.5 in the 72 h sample. Three independent biological samples of each were used in the analysis. Reverse transcription reactions were performed using SuperScript III Reverse Transcriptase (Invitrogen, Carlsbad, CA, USA) with 2 μg total RNA according to the manufacturer’s instructions. The actin gene of *H. glomeratus* (GI: 567319963) was used as an internal control for all of the experiments. The primers for RT-PCR were designed using Primer Express 2.0 software to amplify 100- to 150-bp regions of the chosen genes. The gene-specific primers are listed in Additional file 18: Table S1. qRT-PCR was performed using an ABI ViiA 7 Real Time PCR System (Applied Biosystems, Carlsbad, USA) and SYBR-Green Mastermix (Qiagen, California, USA). The relative expression levels of the selected unigenes normalized to the expression level of actin were calculated from cycle threshold values using the 2^-ΔΔCt^ method.

## Additional files

Additional file 1: Table S2. GO and COG analyses of all unigenes. Additional file 2: Figure S1. Overview of GO function classification of differentially expressed unigenes between control and treatments. Additional file 3: Table S3. KEGG analysis of all unigenes (level 1 and level 2). Additional file 4: Table S4. KEGG analysis of differentially expressed genes between 6 and 0 h (level 1 and level 2). Additional file 5: Table S5. KEGG analysis of differentially expressed genes between 12 and 0 h (level 1 and level 2). Additional file 6: Table S6. KEGG analysis of differentially expressed genes between 24 and 0 h (level 1 and level 2). Additional file 7: Table S7. KEGG analysis of differentially expressed genes between 72 and 0 h (level 1 and level 2). Additional file 8: Figure S2. All unigene CDS prediction. Additional file 9: Table S8. Salt-induced transcripts found in common to at least two stages of salt stress conditions. Additional file 10: Table S9. Function annotation of salt-induced expression unigenes. Additional file 11: Table S10. Function annotation of up-regulated unigenes. Additional file 12: Figure S3. Statistical chart of SSRs and SNPs. Additional file 13:
**KEGG analysis of all unigenes (level 3).**
Additional file 14:
**KEGG analysis of differentially expressed genes between 6 h and 0 h (level3).**
Additional file 15:
**KEGG analysis of differentially expressed genes between 12 h and 0 h (level3).**
Additional file 16:
**KEGG analysis of differentially expressed genes between 24 h and 0 h (level3).**
Additional file 17:
**KEGG analysis of differentially expressed genes between 72 h and 0 h (level3).**
Additional file 18: Table S1. RT-PCR primers.
