# Supplementary material for: Transcriptomic profiling of the salt-stress response in the halophyte Halogeton glomeratus
Source: BMC Genomics. 2015 Mar 11;16(1):169. doi: 10.1186/s12864-015-1373-z (PMC4363069; doi:10.1186/s12864-015-1373-z)

Additional file 2: Figure S1. Overview of GO function classification of differentially expressed unigenes between control and treatments.


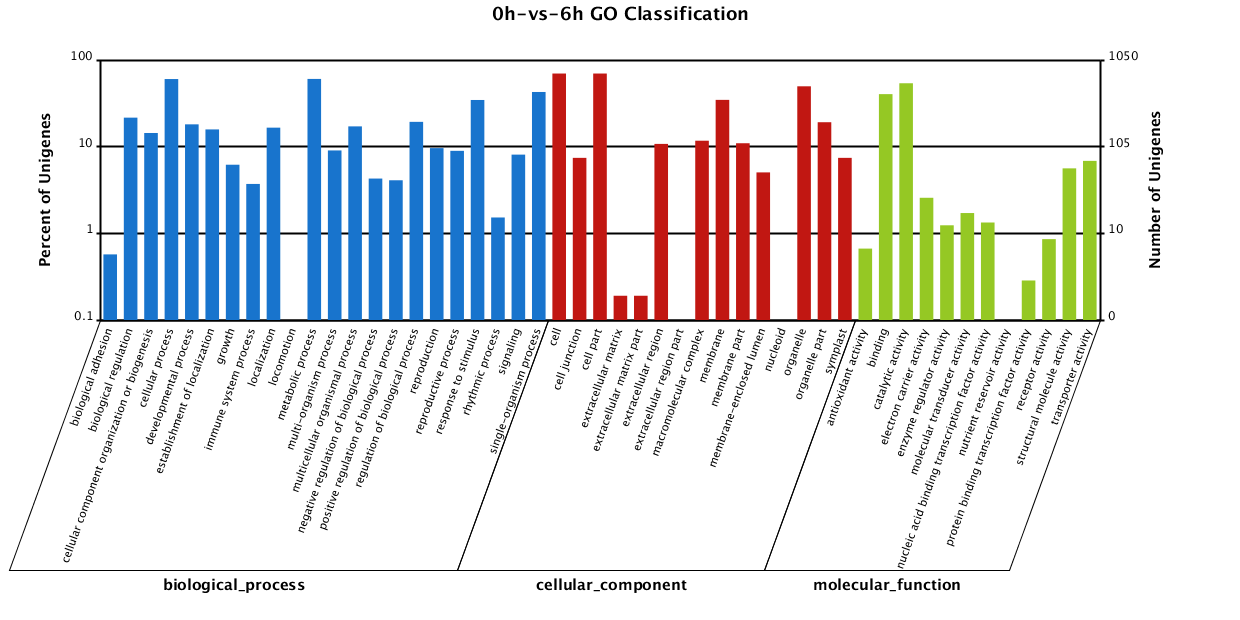


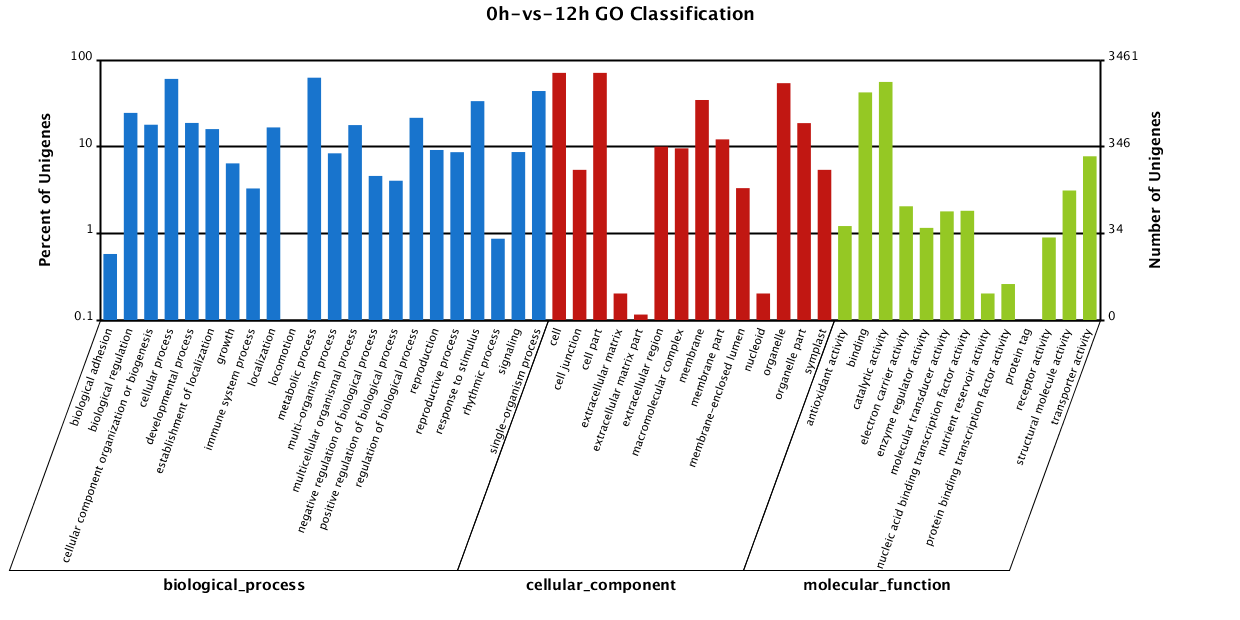


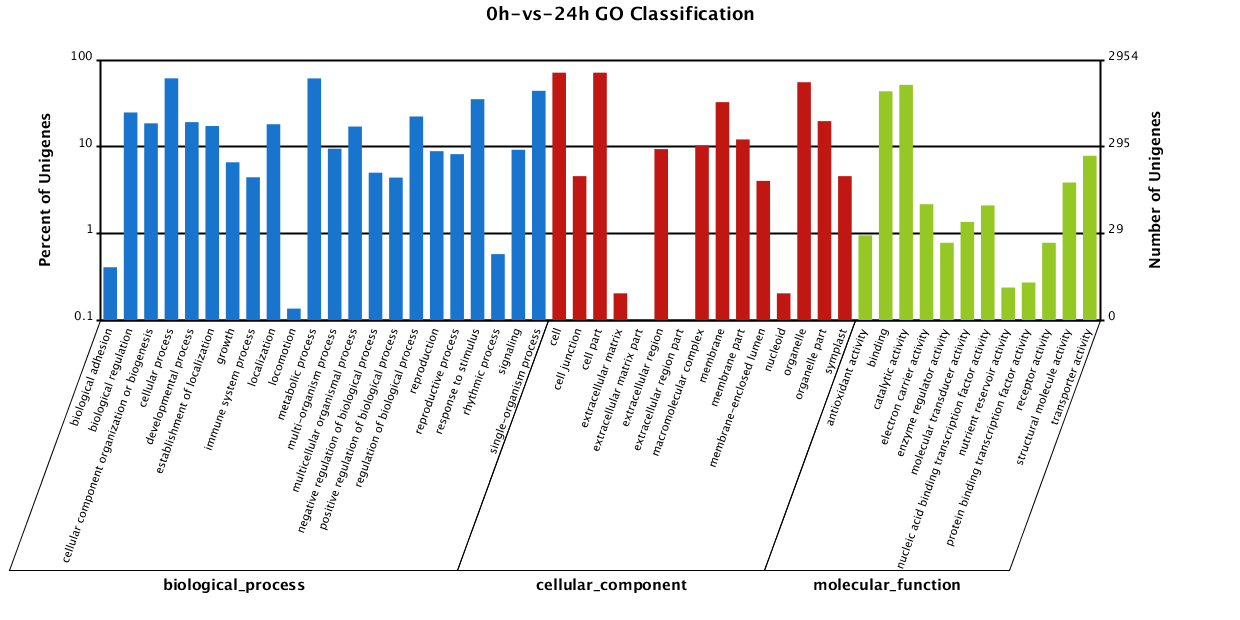


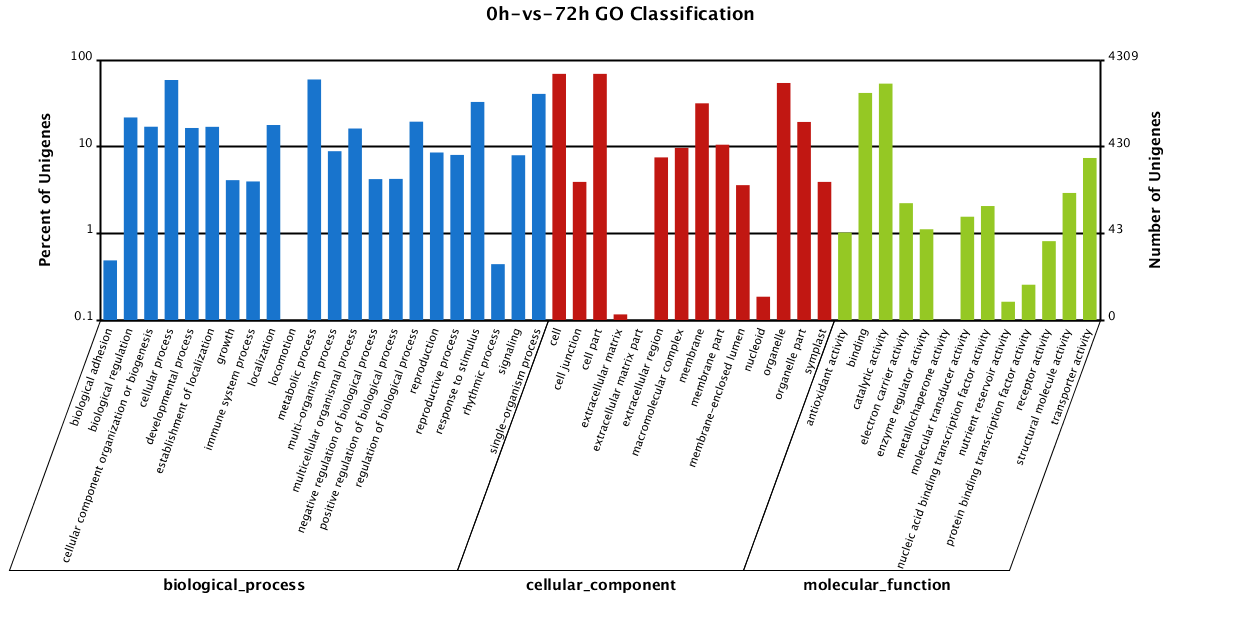

Supplement: Additional file 2: Figure S1. — Overview of GO function classification of differentially expressed unigenes between control and treatments. [file 12864_2015_1373_MOESM2_ESM.doc]
