# Supplementary material for: Transcriptomic profiling of the salt-stress response in the halophyte Halogeton glomeratus
Source: BMC Genomics. 2015 Mar 11;16(1):169. doi: 10.1186/s12864-015-1373-z (PMC4363069; doi:10.1186/s12864-015-1373-z)
Supplement: Additional file 9: Table S8. — Salt-induced transcripts found in common to at least two stages of salt stress conditions. [file 12864_2015_1373_MOESM9_ESM.doc]

Additional file 9: Table S8. Salt-induced transcripts found in common to at least two stages of salt stress conditions

| Gene ID | Length | log2 Fold Change | | | | | Database | E-value | Annonation |
| --- | --- | --- | --- | --- | --- | --- | --- | --- | --- |
|  |  | 6 h | 12 h | | 24 h | 72 h |  |  |  |
| CL5601.Contig1 | 798 |  |  | | 13.43 | 12.77 | NR | 1.0E-60 | Hypothetical protein VITISV_040451[*Vitis vinifera*] |
| CL5609.Contig1 | 799 |  |  | | 12.56 | 9.82 | NR | 4.0E-47 | Hypothetical protein PRUPE_ppb021897mg [*Prunus persica*] |
| CL5645.Contig2 | 973 |  |  | | 12.43 | 12.02 | NR | 2.0E-110 | PREDICTED: uncharacterized protein LOC101261089[*Solanum lycopersicum*] |
| CL5820.Contig1 | 433 |  |  | | 13.01 | 13.40 | NR | 4.0E-16 | peroxidase 6  [*Rubia cordifolia*] |
| Unigene2501 | 363 |  |  | | 10.85 | 13.16 | NT | 6.0E-06 | Populus EST from mild drought-stressed leaves |
| Unigene2603 | 427 |  | |  | 14.80 | 10.81 | NT | 2.0E-21 | PREDICTED: Solanum lycopersicum serine hydroxymethyl transferase 1-like |
| Unigene1996 | 934 |  |  | | 10.38 | 11.93 | Swissprot | 2.0E-08 | Retrovirus-related Pol polyprotein from transposon TNT 1-94 [*Nicotiana tabacum*] |
| Unigene2562 | 351 |  |  | | 11.00 | 10.91 | Swissprot | 5.0E-09 | 3 ubiquitin-protein ligase PUB23 [*Arabidopsis thaliana*] |
| Unigene2702 | 439 | 10.67 |  | | 11.29 | 13.20 | Swissprot | 1.0E-05 | Splicing factor U2af large subunit A  [*Oryza sativa subsp. Japonica*] |
| Unigene5334 | 352 |  |  | | 10.79 | 11.32 | Swissprot | 4.0E-36 | ATP-citrate synthase alpha chain protein 3 [*Oryza sativa subsp.Japonica*] |
| Unigene7663 | 272 |  |  | | 11.46 | 11.18 | Swissprot | 2.0E-11 | Vegetative cell wall protein gp1 [*Chlamydomonas reinhardtii*] |
| Unigene8298 | 586 |  |  | | 11.05 | 11.69 | Swissprot | 4.0E-08 | Protein ycf2  [*Oenothera elata subsp. Hookeri*] |
| Unigene8306 | 336 | 11.33 |  | | 10.96 | 11.24 | Swissprot | 4.0E-08 | Vacuolar proton ATPase a2 [*Arabidopsis thaliana*] |
| CL39.Contig1 | 861 |  |  | | 11.41 | 12.54 | COG | 5.0E-16 | General function prediction only |
| CL39.Contig2 | 705 |  |  | | 10.61 | 11.42 | COG | 4.0E-10 | General function prediction only |
| CL45.Contig1 | 957 |  |  | | 11.07 | 12.09 | COG | 1.0E-28 | Nucleotide transport and metabolism |
| Unigene13477 | 386 | 10.85 | 11.98 | | 12.31 | 11.26 | COG | 6.0E-07 | Posttranslational modification, protein turnover, chaperones |
| CL1932.Contig4 | 253 |  |  | | 12.73 | 11.28 | GO |  | GO:0005618 |
| CL2127.Contig1 | 715 |  |  | | 12.19 | 12.28 | GO |  | GO:0005737 |
| CL2401.Contig4 | 532 |  |  | | 13.19 | 12.29 | GO |  | GO:0005516 |
| CL3112.Contig1 | 473 |  |  | | 13.86 | 11.16 | GO |  | GO:0006979 |
| CL532.Contig1 | 292 | 11.35 |  | | 11.26 | 12.73 | GO |  | GO:0016787 |
| Unigene13497 | 772 |  |  | | 11.27 | 11.65 | GO |  | GO:0009987 |
| Unigene14013 | 318 |  |  | | 11.62 | 12.31 | GO |  | GO:0080118 |
| Unigene19406 | 332 |  |  | | 12.30 | 11.78 | GO |  | GO:0005737 |
| Unigene2465 | 419 |  |  | | 11.42 | 10.92 | GO |  | GO:0003676 |
| Unigene24859 | 680 |  |  | | 11.04 | 12.76 | GO |  | GO:0031981 |
| Unigene27641 | 313 |  | 10.94 | | 11.34 | 11.49 | GO |  | GO:0009827 |
| Unigene27774 | 302 |  |  | | 11.11 | 11.74 | GO |  | GO:0044710 |
| Unigene32212 | 1359 |  |  | | 12.82 | 9.14 | GO |  | GO:0006468 |
| Unigene32430 | 297 |  |  | | 12.19 | 10.94 | GO |  | GO:0003899 |
| CL1103.Contig1 | 713 |  |  | | 9.97 | 9.68 |  |  | Unknown |
| CL1103.Contig2 | 1218 |  |  | | 12.76 | 12.75 |  |  | Unknown |
| CL1242.Contig4 | 473 |  |  | | 12.64 | 12.35 |  |  | Unknown |
| CL1397.Contig2 | 533 |  |  | | 10.29 | 10.40 |  |  | Unknown |
| CL1533.Contig2 | 295 |  |  | | 11.43 | 11.58 |  |  | Unknown |
| CL1752.Contig1 | 744 |  |  | | 10.25 | 11.75 |  |  | Unknown |
| CL1857.Contig2 | 339 |  |  | | 15.67 | 16.00 |  |  | Unknown |
| CL2563.Contig3 | 2361 | 8.13 |  | | 8.25 | 10.71 |  |  | Unknown |
| CL278.Contig2 | 227 | 12.20 |  | | 11.72 | 13.15 |  |  | Unknown |
| CL2797.Contig3 | 493 |  |  | | 10.85 | 11.27 |  |  | Unknown |
| CL3108.Contig1 | 337 |  |  | | 11.15 | 10.76 |  |  | Unknown |
| CL3173.Contig2 | 310 |  | 12.42 | | 12.73 | 13.43 |  |  | Unknown |
| CL3550.Contig2 | 584 |  |  | | 11.35 | 12.38 |  |  | Unknown |
| CL3671.Contig3 | 481 |  |  | | 11.84 | 11.50 |  |  | Unknown |
| CL4080.Contig1 | 1196 |  |  | | 12.66 | 9.32 |  |  | Unknown |
| CL4703.Contig2 | 241 |  |  | | 11.54 | 12.07 |  |  | Unknown |
| CL4872.Contig2 | 495 |  |  | | 10.40 | 12.66 |  |  | Unknown |
| CL5054.Contig1 | 344 |  |  | | 12.90 | 11.56 |  |  | Unknown |
| CL5088.Contig1 | 461 |  |  | | 10.60 | 11.82 |  |  | Unknown |
| CL5227.Contig1 | 252 |  |  | | 11.57 | 11.94 |  |  | Unknown |
| CL5237.Contig1 | 220 |  |  | | 11.93 | 11.85 |  |  | Unknown |
| CL5525.Contig2 | 389 |  |  | | 11.11 | 11.02 |  |  | Unknown |
| CL6067.Contig1 | 413 |  |  | | 12.50 | 11.52 |  |  | Unknown |
| CL681.Contig3 | 230 |  |  | | 11.87 | 13.28 |  |  | Unknown |
| CL871.Contig1 | 1111 | 10.84 | 9.94 | | 10.07 | 11.68 |  |  | Unknown |
| Unigene13303 | 304 | 11.09 | 11.09 | | 11.30 | 12.12 |  |  | Unknown |
| Unigene13528 | 497 |  |  | | 12.54 | 11.26 |  |  | Unknown |
| Unigene13837 | 343 |  |  | | 11.12 | 11.68 |  |  | Unknown |
| Unigene13878 | 450 |  |  | | 11.19 | 12.10 |  |  | Unknown |
| Unigene13915 | 696 |  |  | | 12.64 | 10.41 |  |  | Unknown |
| Unigene14012 | 247 |  |  | | 11.40 | 11.21 |  |  | Unknown |
| Unigene15975 | 341 |  | 11.20 | | 10.94 | 11.29 |  |  | Unknown |
| Unigene16491 | 287 |  |  | | 11.84 | 14.75 |  |  | Unknown |
| Unigene16600 | 815 |  |  | | 11.76 | 10.69 |  |  | Unknown |
| Unigene16689 | 279 |  |  | | 11.23 | 12.09 |  |  | Unknown |
| Unigene18828 | 585 |  |  | | 11.60 | 11.76 |  |  | Unknown |
| Unigene18968 | 517 |  |  | | 10.70 | 11.69 |  |  | Unknown |
| Unigene19095 | 749 |  |  | | 11.72 | 13.41 |  |  | Unknown |
| Unigene19352 | 448 |  |  | | 12.29 | 12.67 |  |  | Unknown |
| Unigene19412 | 349 |  |  | | 12.62 | 11.47 |  |  | Unknown |
| Unigene19458 | 373 |  |  | | 11.81 | 13.51 |  |  | Unknown |
| Unigene19538 | 291 |  |  | | 11.27 | 12.83 |  |  | Unknown |
| Unigene2092 | 610 |  |  | | 11.34 | 10.60 |  |  | Unknown |
| Unigene21033 | 1286 |  |  | | 12.07 | 11.17 |  |  | Unknown |
| Unigene21311 | 625 |  |  | | 11.78 | 11.30 |  |  | Unknown |
| Unigene21765 | 342 |  |  | | 11.45 | 10.85 |  |  | Unknown |
| Unigene21787 | 397 |  |  | | 10.91 | 11.53 |  |  | Unknown |
| Unigene21851 | 299 |  |  | | 12.13 | 11.04 |  |  | Unknown |
| Unigene22070 | 389 |  |  | | 14.22 | 14.19 |  |  | Unknown |
| Unigene22097 | 444 |  |  | | 11.61 | 12.09 |  |  | Unknown |
| Unigene22121 | 331 |  |  | | 11.42 | 11.34 |  |  | Unknown |
| Unigene22136 | 362 |  |  | | 13.38 | 13.28 |  |  | Unknown |
| Unigene2253 | 288 |  |  | | 11.18 | 12.75 |  |  | Unknown |
| Unigene2271 | 326 |  |  | | 11.28 | 11.20 |  |  | Unknown |
| Unigene2293 | 432 |  |  | | 10.60 | 11.91 |  |  | Unknown |
| Unigene2321 | 402 |  |  | | 10.98 | 12.78 |  |  | Unknown |
| Unigene24595 | 846 |  |  | | 11.77 | 11.06 |  |  | Unknown |
| Unigene24658 | 276 |  |  | | 12.83 | 13.26 |  |  | Unknown |
| Unigene24879 | 331 |  |  | | 11.08 | 10.79 |  |  | Unknown |
| Unigene24927 | 327 |  |  | | 12.05 | 11.10 |  |  | Unknown |
| Unigene24964 | 456 |  | 10.40 | | 11.03 | 12.77 |  |  | Unknown |
| Unigene25076 | 230 |  |  | | 12.02 | 11.61 |  |  | Unknown |
| Unigene25107 | 303 |  |  | | 11.69 | 11.12 |  |  | Unknown |
| Unigene2533 | 316 |  |  | | 11.33 | 11.40 |  |  | Unknown |
| Unigene2704 | 342 |  |  | | 10.93 | 12.29 |  |  | Unknown |
| Unigene27481 | 261 | 11.31 |  | | 11.32 | 11.24 |  |  | Unknown |
| Unigene27588 | 368 |  |  | | 11.11 | 11.69 |  |  | Unknown |
| Unigene27722 | 678 |  |  | | 12.51 | 11.26 |  |  | Unknown |
| Unigene27825 | 252 |  |  | | 11.37 | 11.73 |  |  | Unknown |
| Unigene3142 | 369 |  |  | | 12.74 | 10.63 |  |  | Unknown |
| Unigene31668 | 237 |  |  | | 11.74 | 12.32 |  |  | Unknown |
| Unigene31674 | 203 |  |  | | 11.58 | 13.22 |  |  | Unknown |
| Unigene31896 | 480 |  |  | | 14.25 | 10.55 |  |  | Unknown |
| Unigene32332 | 497 |  |  | | 13.06 | 10.31 |  |  | Unknown |
| Unigene32333 | 337 |  |  | | 13.30 | 11.15 |  |  | Unknown |
| Unigene33627 | 439 |  |  | | 11.41 | 12.91 |  |  | Unknown |
| Unigene33828 | 465 |  |  | | 10.49 | 12.55 |  |  | Unknown |
| Unigene33874 | 597 |  |  | | 10.41 | 12.85 |  |  | Unknown |
| Unigene34165 | 397 |  |  | | 10.82 | 12.95 |  |  | Unknown |
| Unigene34281 | 272 |  | 11.35 | | 11.46 | 12.62 |  |  | Unknown |
| Unigene34317 | 321 |  |  | | 11.03 | 12.18 |  |  | Unknown |
| Unigene34489 | 468 |  |  | | 10.92 | 11.76 |  |  | Unknown |
| Unigene5175 | 288 |  |  | | 11.18 | 12.50 |  |  | Unknown |
| Unigene6804 | 718 |  |  | | 11.23 | 13.27 |  |  | Unknown |
| Unigene7547 | 1793 |  |  | | 14.16 | 13.68 |  |  | Unknown |
| Unigene8196 | 374 |  |  | | 11.17 | 11.16 |  |  | Unknown |
| Unigene9427 | 829 |  |  | | 12.29 | 11.38 |  |  | Unknown |

Note: The transcript abundance changes (log2 fold) based on RPKM values according to RNA-Seq.
