# Supplementary material for: Transcriptomic profiling of the salt-stress response in the halophyte Halogeton glomeratus
Source: BMC Genomics. 2015 Mar 11;16(1):169. doi: 10.1186/s12864-015-1373-z (PMC4363069; doi:10.1186/s12864-015-1373-z)
Supplement: Additional file 12: Figure S3. — Statistical chart of SSRs and SNPs. [file 12864_2015_1373_MOESM12_ESM.doc]

Additional file 12: Figure S3. Statistical chart of SSRs (A) and SNPs (B).

(A)
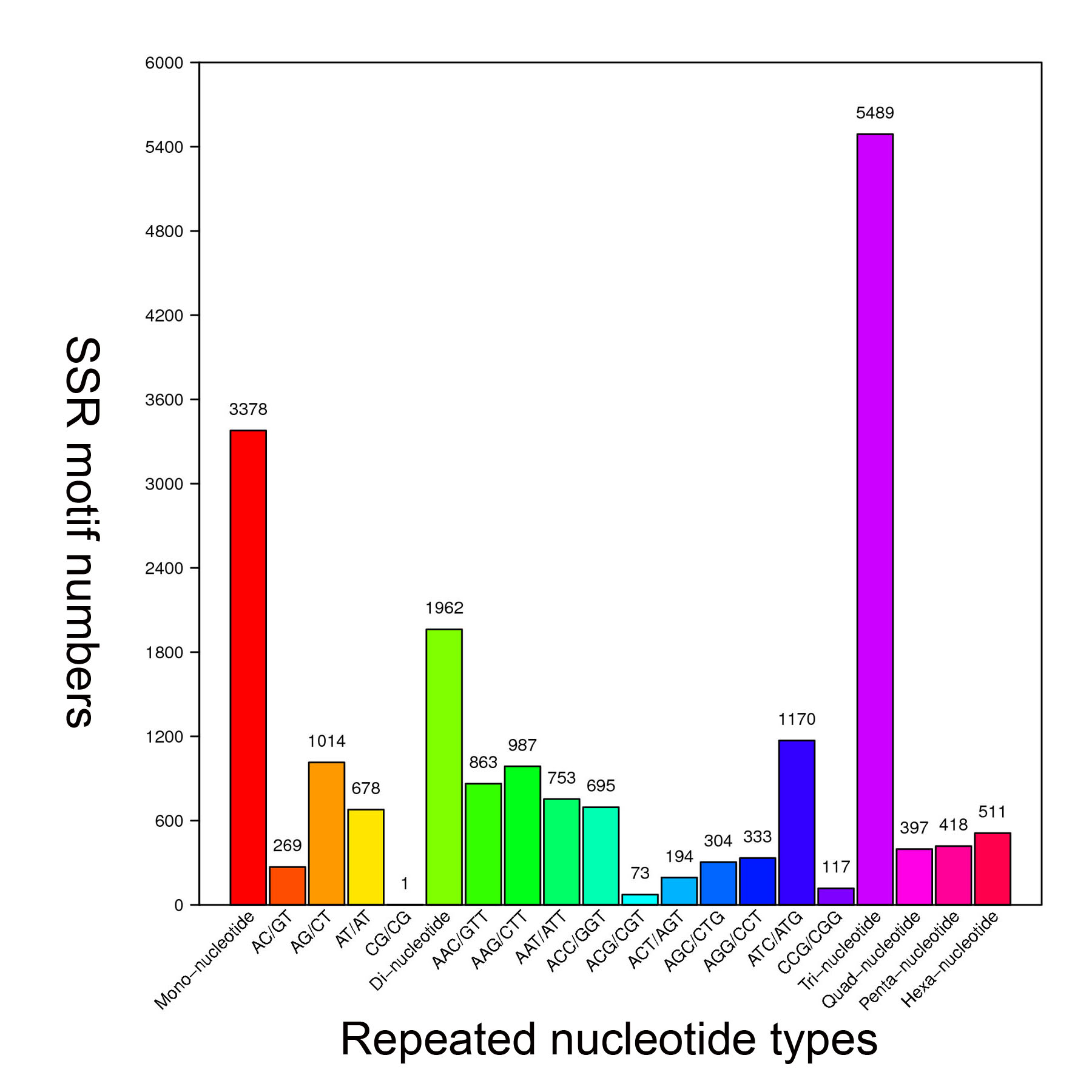


Note: The X-axis is the repeat times of repeat units. The Y-axis is the number of SSRs.

(B)
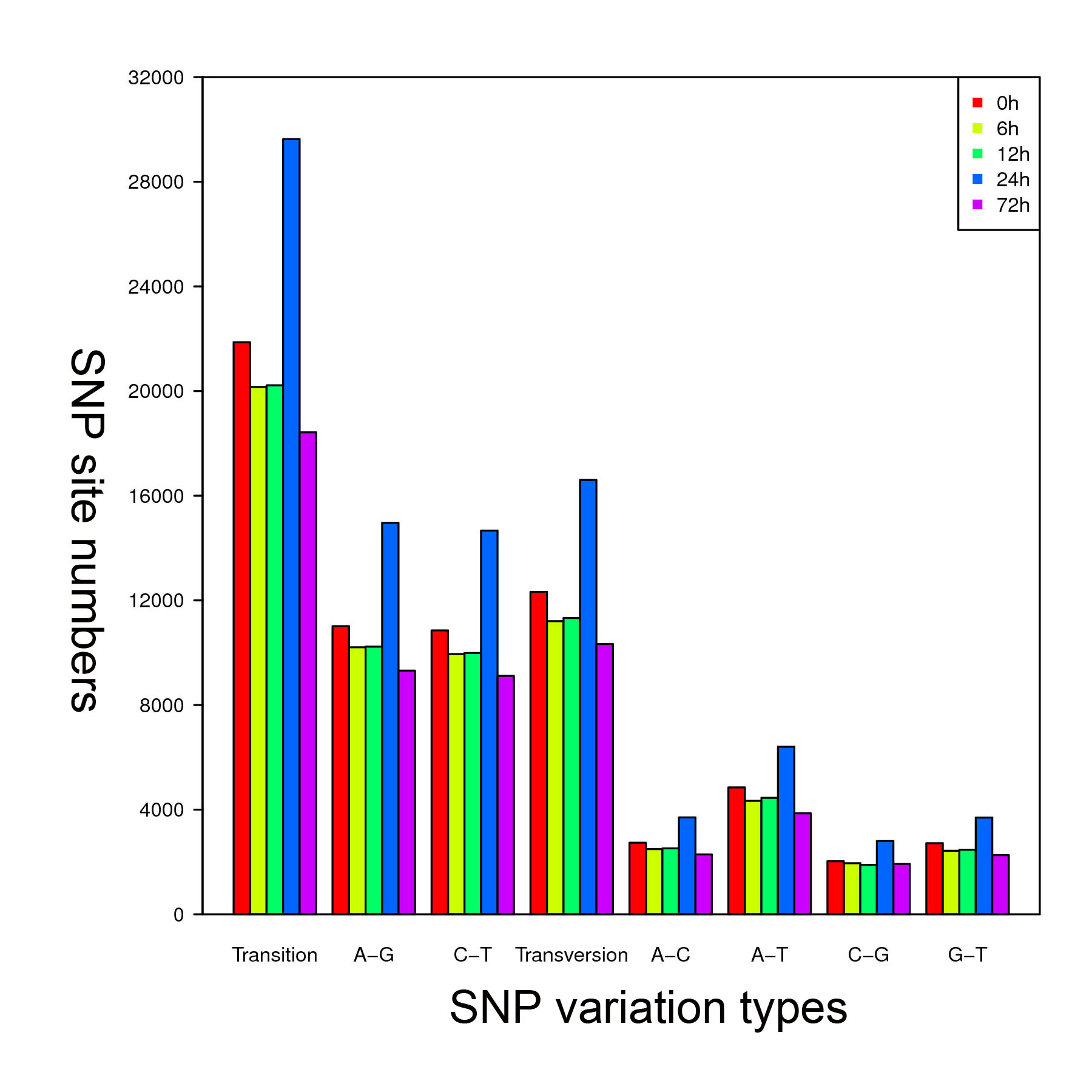


Note: The X-axis is SNP types, the Y-axis is the number of SNP.
