# Supplementary figures and images for: Transcriptomic profiling of the salt-stress response in the halophyte Halogeton glomeratus (part 5 of 5)
Source: BMC Genomics. 2015 Mar 11;16(1):169. doi: 10.1186/s12864-015-1373-z (PMC4363069; doi:10.1186/s12864-015-1373-z)

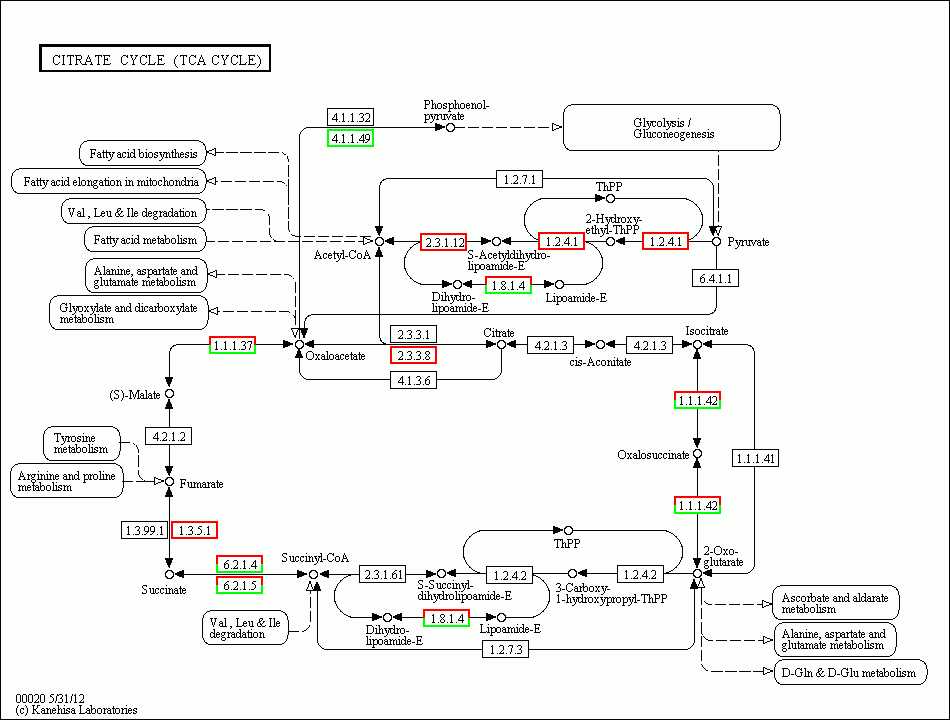

Supplement: Additional file 17: — KEGG analysis of differentially expressed genes between 72 h and 0 h (level3). [file 12864_2015_1373_MOESM17_ESM.zip › Additional file 5. KEGG analysis of differentially expressed genes between 72 h and 0 h (level3)/map00020.png]

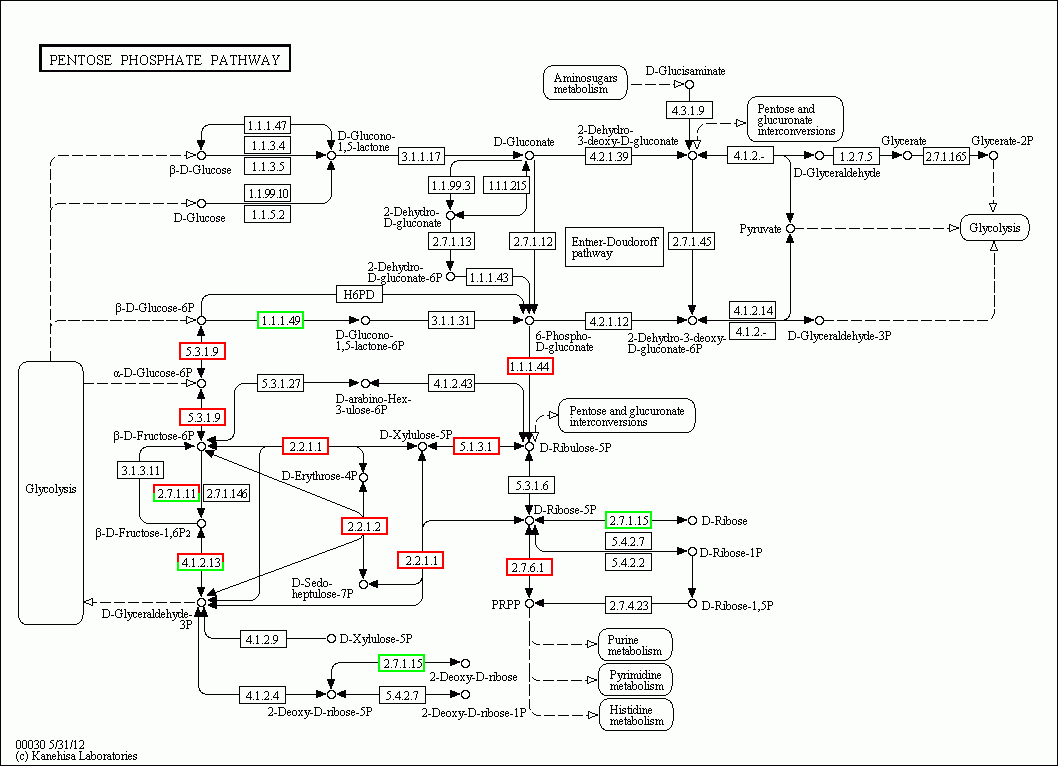

Supplement: Additional file 17: — KEGG analysis of differentially expressed genes between 72 h and 0 h (level3). [file 12864_2015_1373_MOESM17_ESM.zip › Additional file 5. KEGG analysis of differentially expressed genes between 72 h and 0 h (level3)/map00030.png]

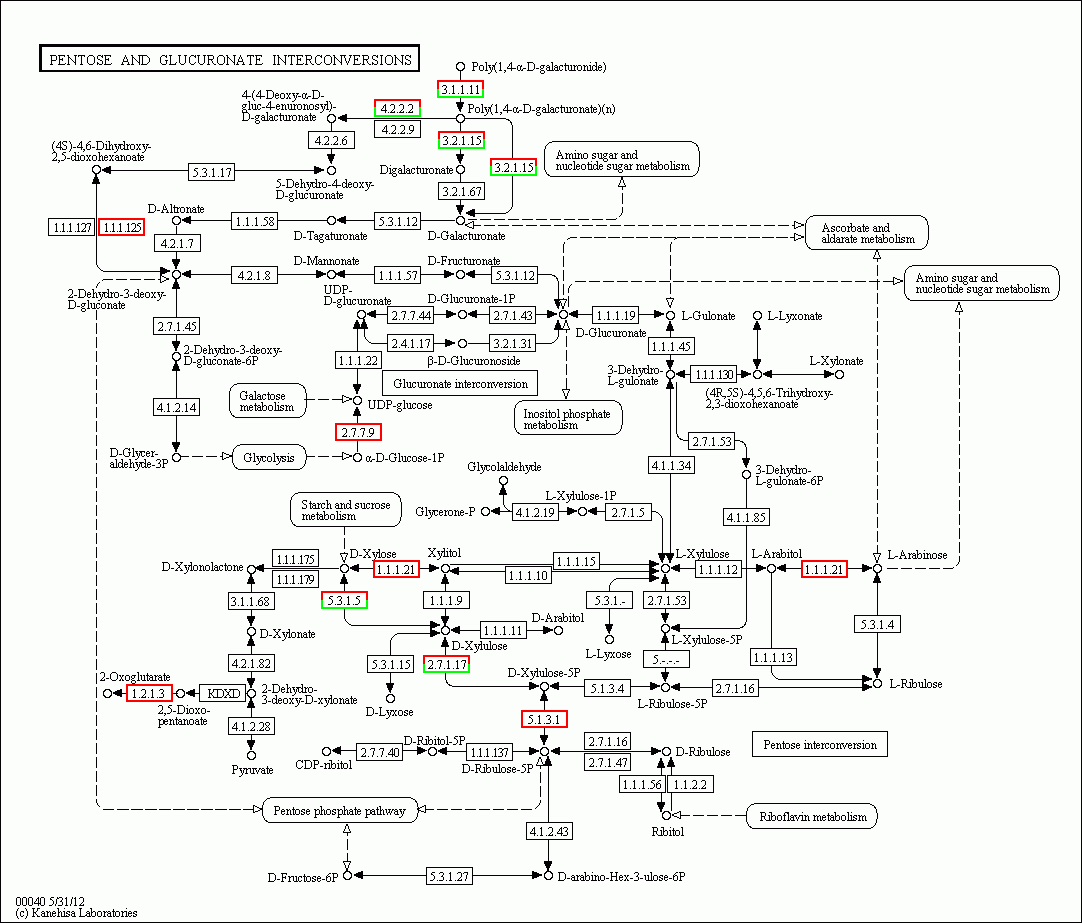

Supplement: Additional file 17: — KEGG analysis of differentially expressed genes between 72 h and 0 h (level3). [file 12864_2015_1373_MOESM17_ESM.zip › Additional file 5. KEGG analysis of differentially expressed genes between 72 h and 0 h (level3)/map00040.png]

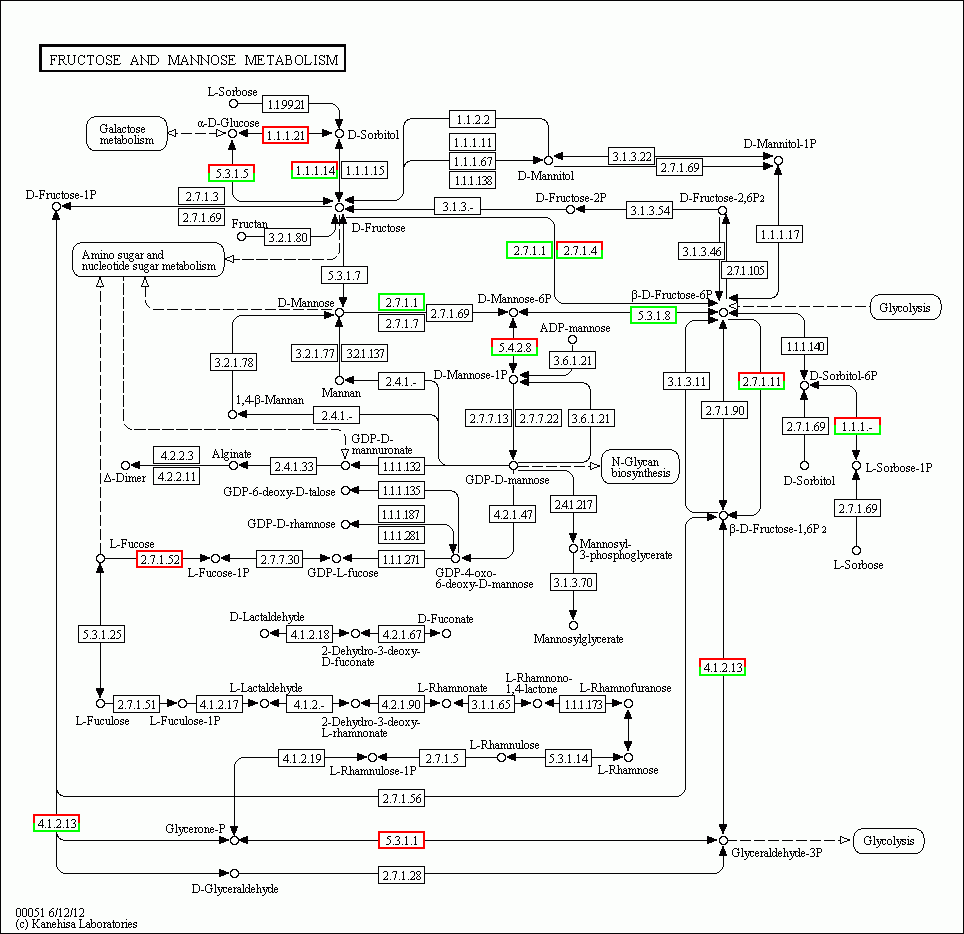

Supplement: Additional file 17: — KEGG analysis of differentially expressed genes between 72 h and 0 h (level3). [file 12864_2015_1373_MOESM17_ESM.zip › Additional file 5. KEGG analysis of differentially expressed genes between 72 h and 0 h (level3)/map00051.png]

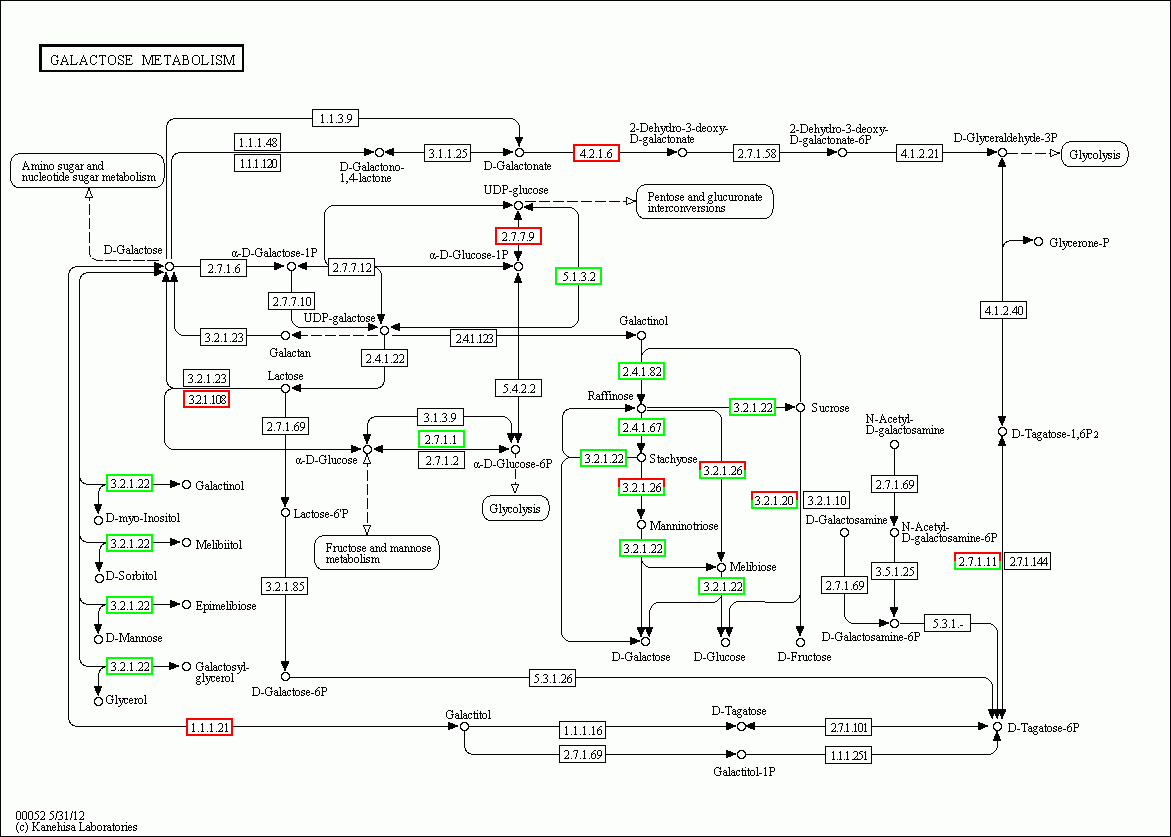

Supplement: Additional file 17: — KEGG analysis of differentially expressed genes between 72 h and 0 h (level3). [file 12864_2015_1373_MOESM17_ESM.zip › Additional file 5. KEGG analysis of differentially expressed genes between 72 h and 0 h (level3)/map00052.png]

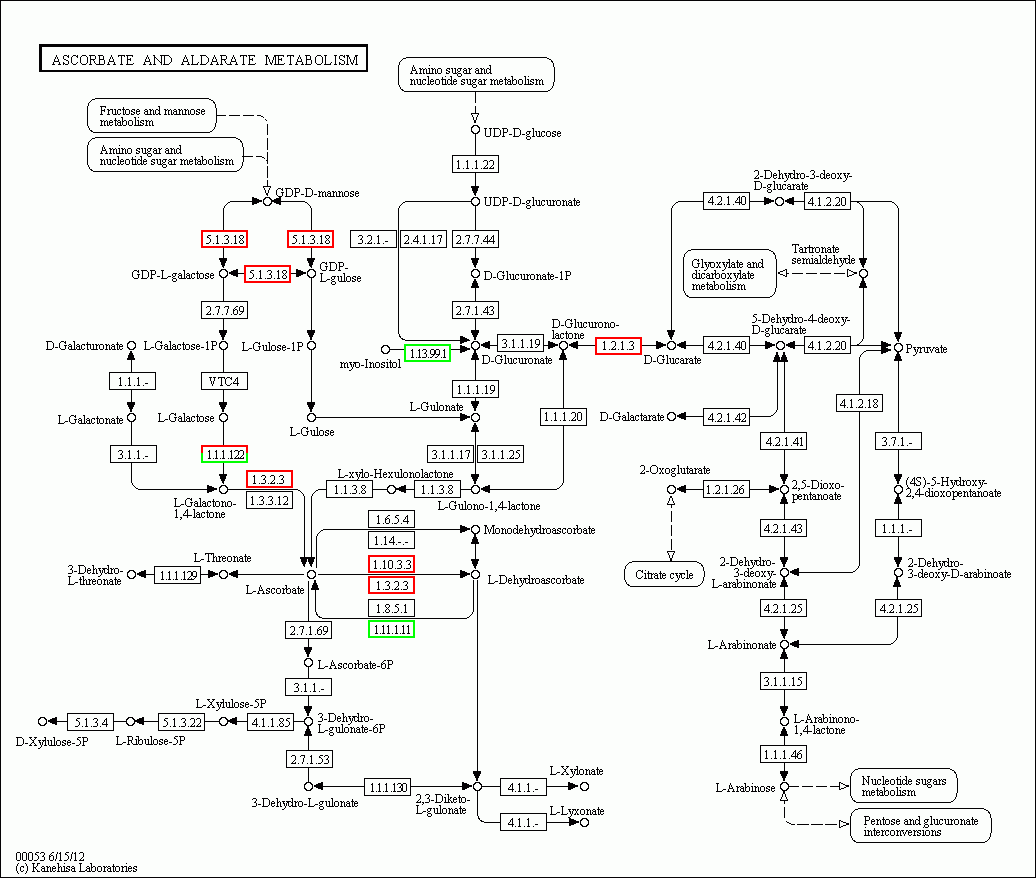

Supplement: Additional file 17: — KEGG analysis of differentially expressed genes between 72 h and 0 h (level3). [file 12864_2015_1373_MOESM17_ESM.zip › Additional file 5. KEGG analysis of differentially expressed genes between 72 h and 0 h (level3)/map00053.png]

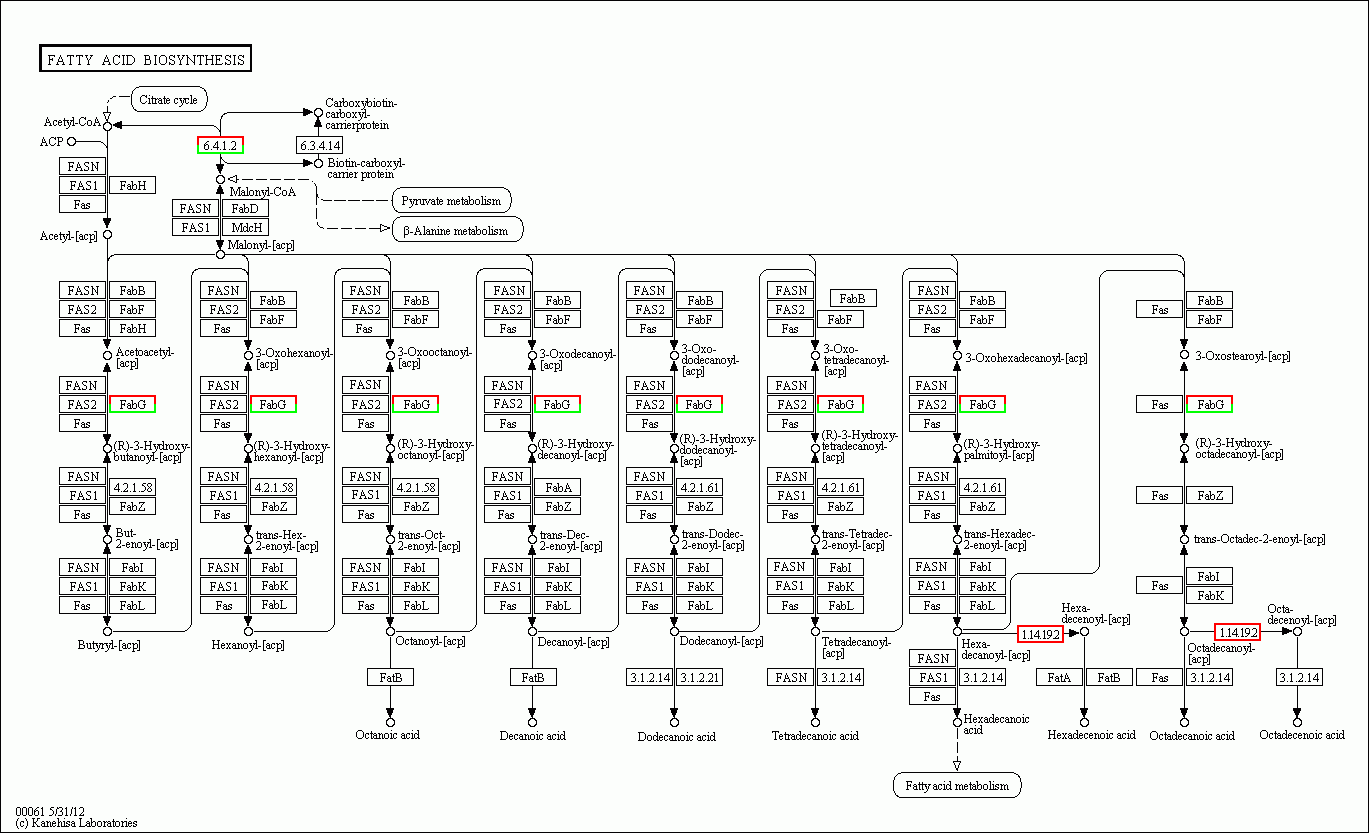

Supplement: Additional file 17: — KEGG analysis of differentially expressed genes between 72 h and 0 h (level3). [file 12864_2015_1373_MOESM17_ESM.zip › Additional file 5. KEGG analysis of differentially expressed genes between 72 h and 0 h (level3)/map00061.png]

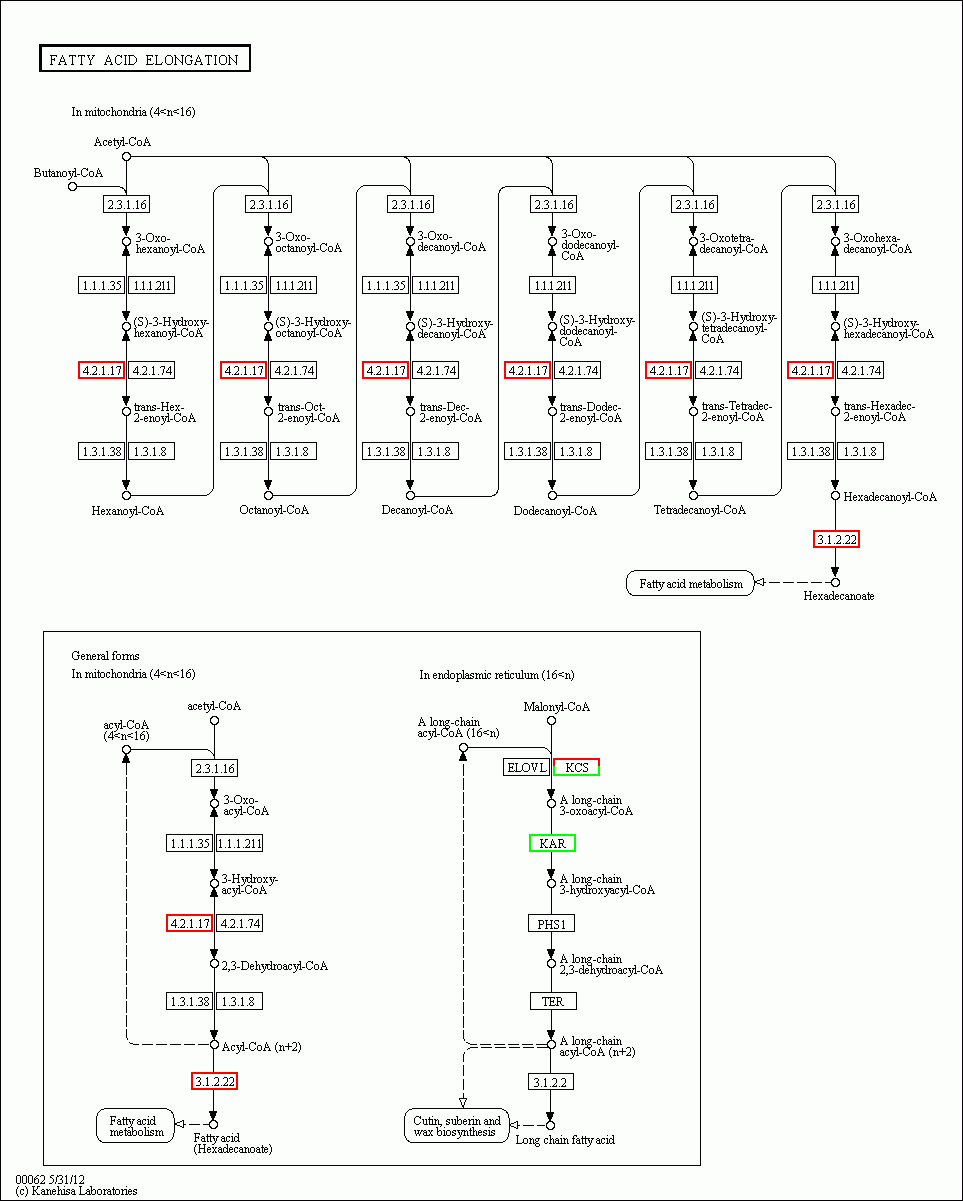

Supplement: Additional file 17: — KEGG analysis of differentially expressed genes between 72 h and 0 h (level3). [file 12864_2015_1373_MOESM17_ESM.zip › Additional file 5. KEGG analysis of differentially expressed genes between 72 h and 0 h (level3)/map00062.png]

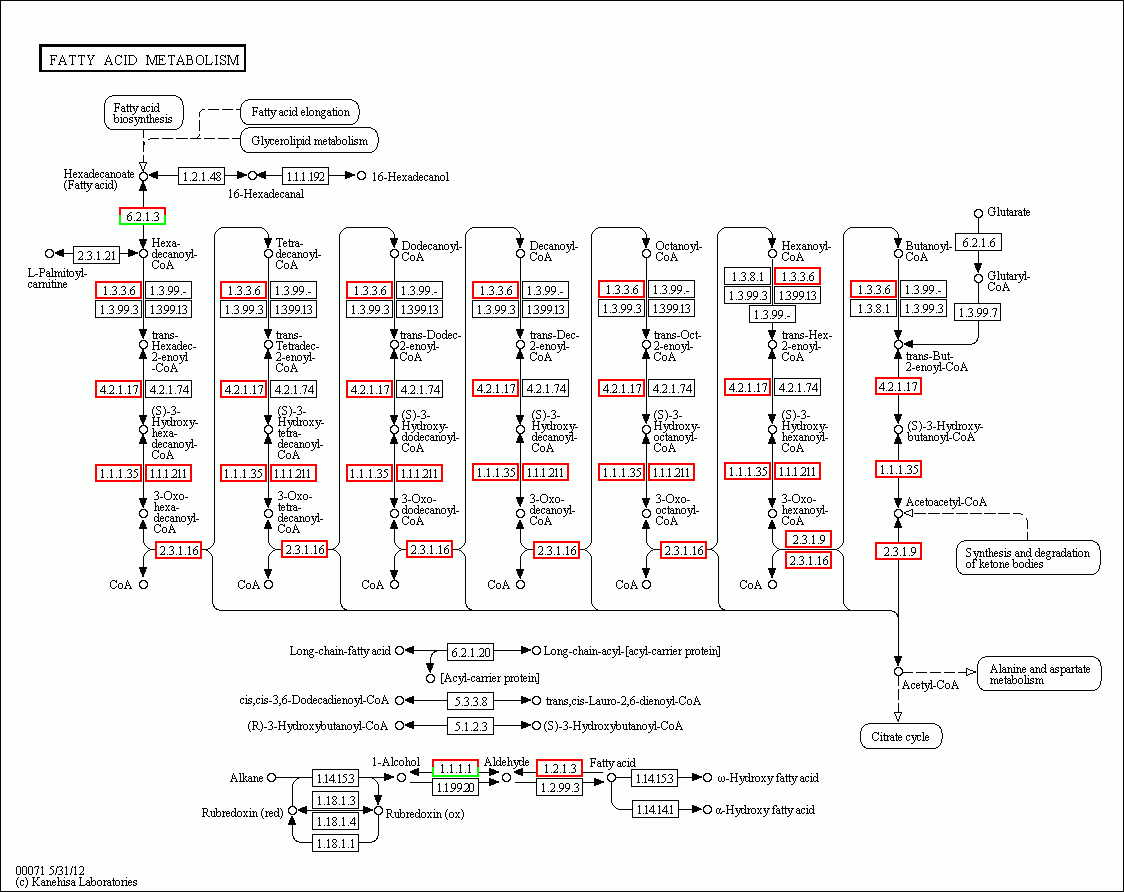

Supplement: Additional file 17: — KEGG analysis of differentially expressed genes between 72 h and 0 h (level3). [file 12864_2015_1373_MOESM17_ESM.zip › Additional file 5. KEGG analysis of differentially expressed genes between 72 h and 0 h (level3)/map00071.png]

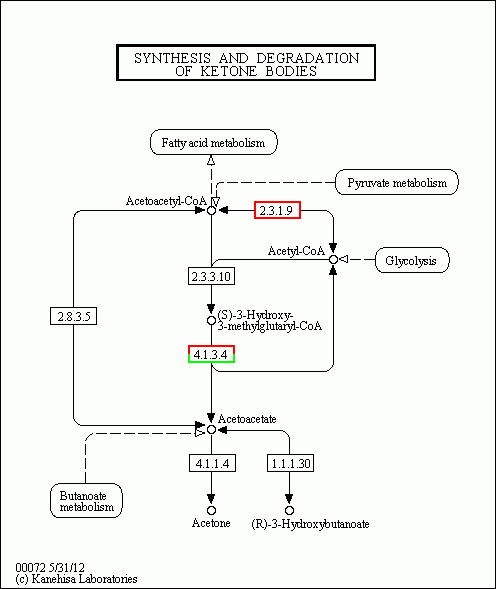

Supplement: Additional file 17: — KEGG analysis of differentially expressed genes between 72 h and 0 h (level3). [file 12864_2015_1373_MOESM17_ESM.zip › Additional file 5. KEGG analysis of differentially expressed genes between 72 h and 0 h (level3)/map00072.png]

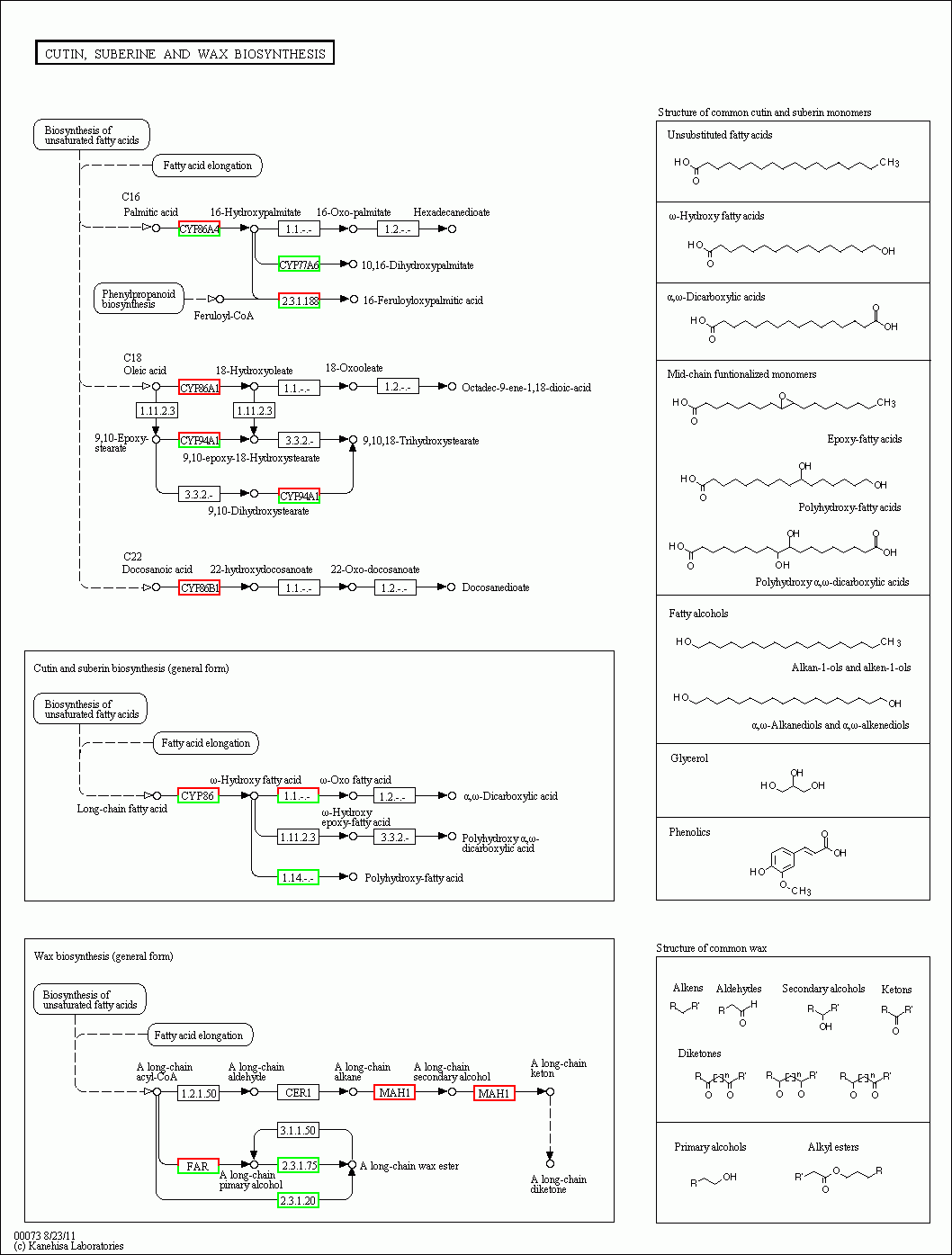

Supplement: Additional file 17: — KEGG analysis of differentially expressed genes between 72 h and 0 h (level3). [file 12864_2015_1373_MOESM17_ESM.zip › Additional file 5. KEGG analysis of differentially expressed genes between 72 h and 0 h (level3)/map00073.png]

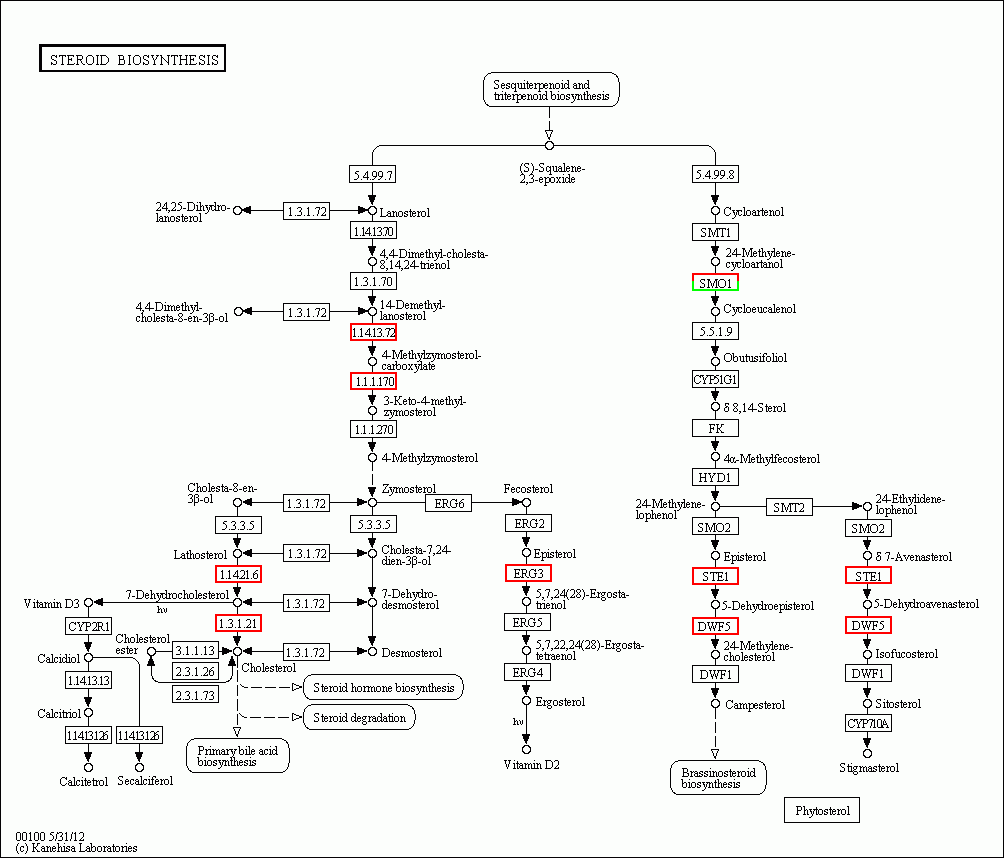

Supplement: Additional file 17: — KEGG analysis of differentially expressed genes between 72 h and 0 h (level3). [file 12864_2015_1373_MOESM17_ESM.zip › Additional file 5. KEGG analysis of differentially expressed genes between 72 h and 0 h (level3)/map00100.png]

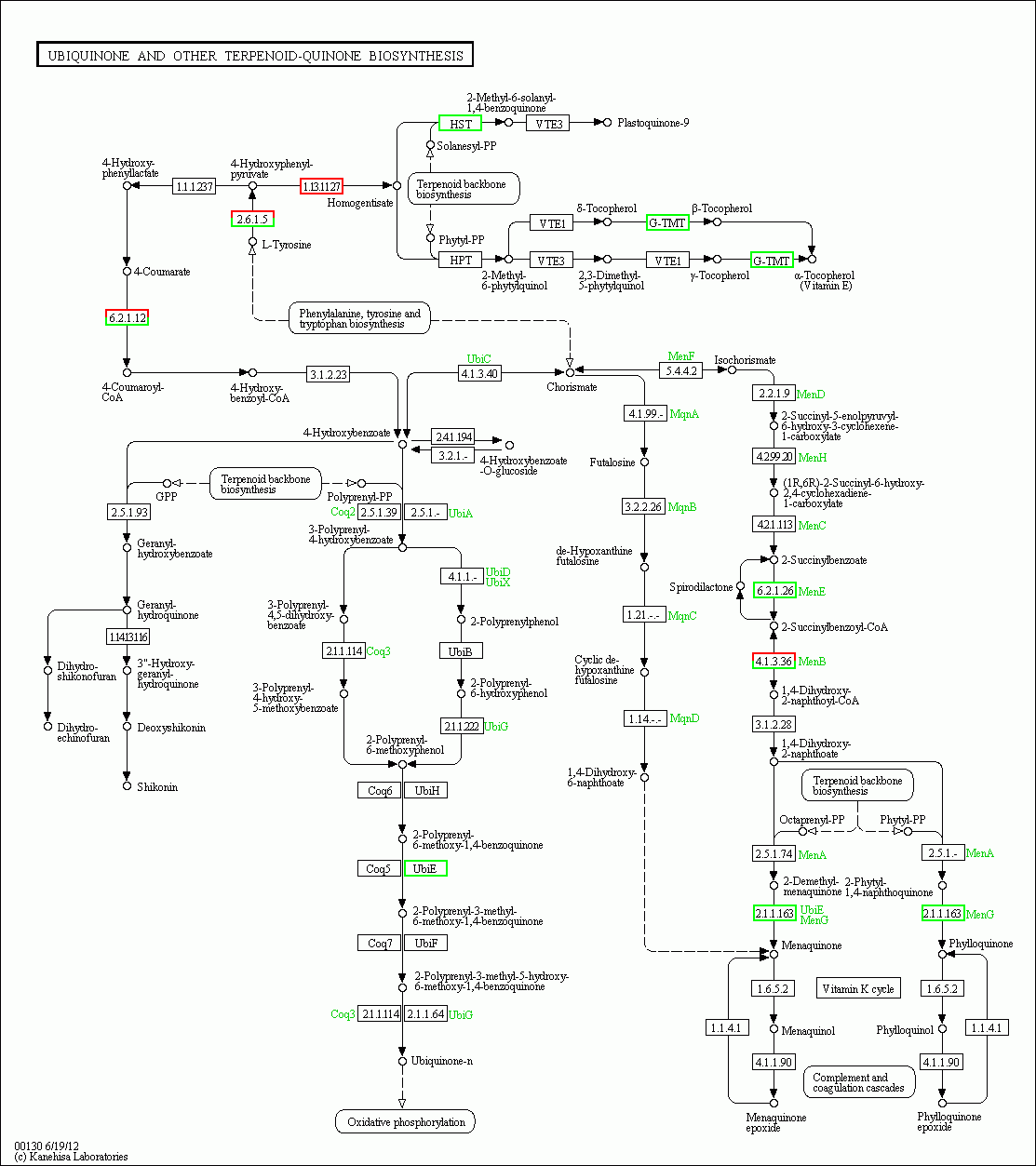

Supplement: Additional file 17: — KEGG analysis of differentially expressed genes between 72 h and 0 h (level3). [file 12864_2015_1373_MOESM17_ESM.zip › Additional file 5. KEGG analysis of differentially expressed genes between 72 h and 0 h (level3)/map00130.png]

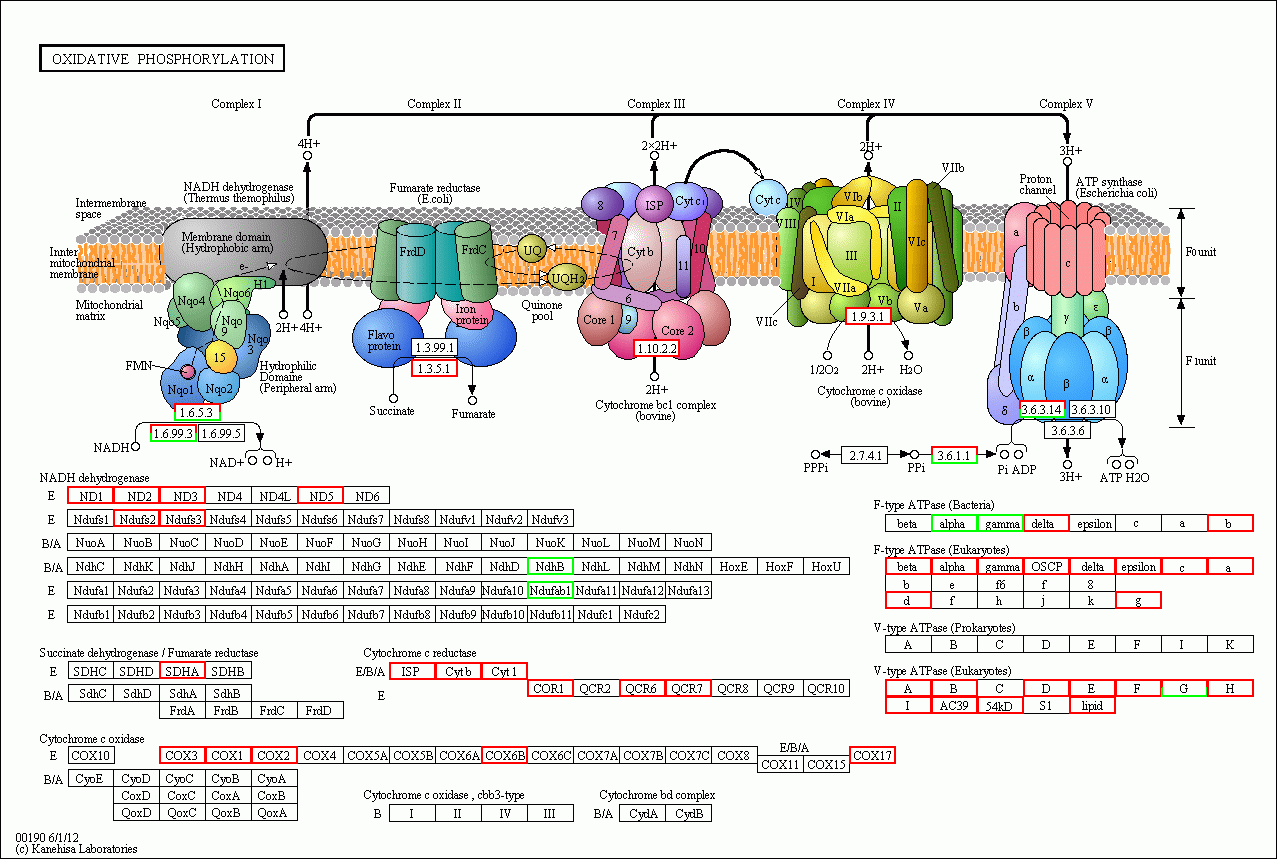

Supplement: Additional file 17: — KEGG analysis of differentially expressed genes between 72 h and 0 h (level3). [file 12864_2015_1373_MOESM17_ESM.zip › Additional file 5. KEGG analysis of differentially expressed genes between 72 h and 0 h (level3)/map00190.png]

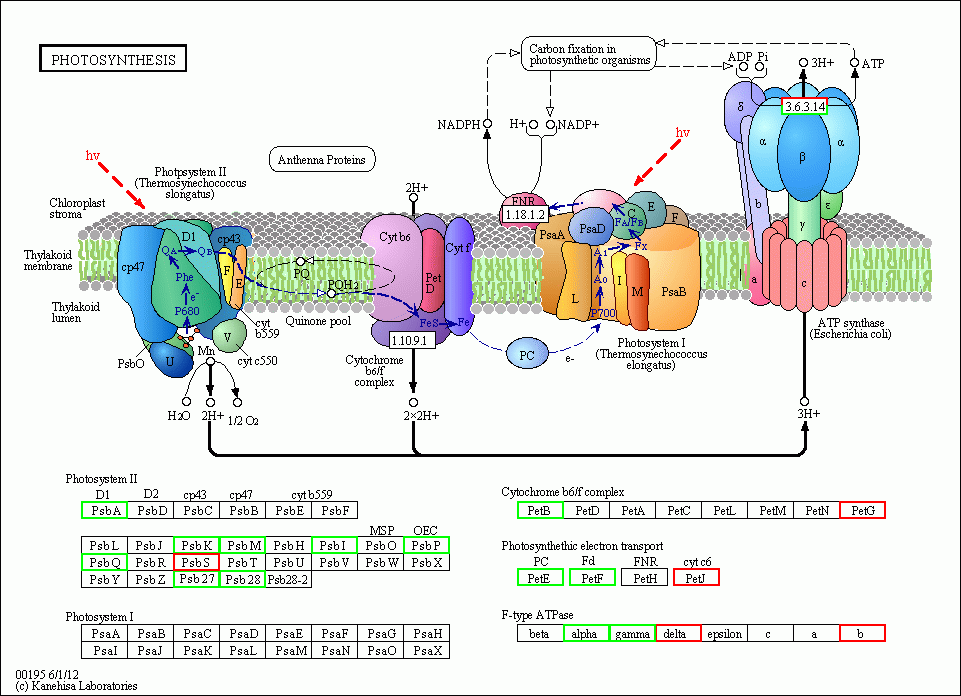

Supplement: Additional file 17: — KEGG analysis of differentially expressed genes between 72 h and 0 h (level3). [file 12864_2015_1373_MOESM17_ESM.zip › Additional file 5. KEGG analysis of differentially expressed genes between 72 h and 0 h (level3)/map00195.png]

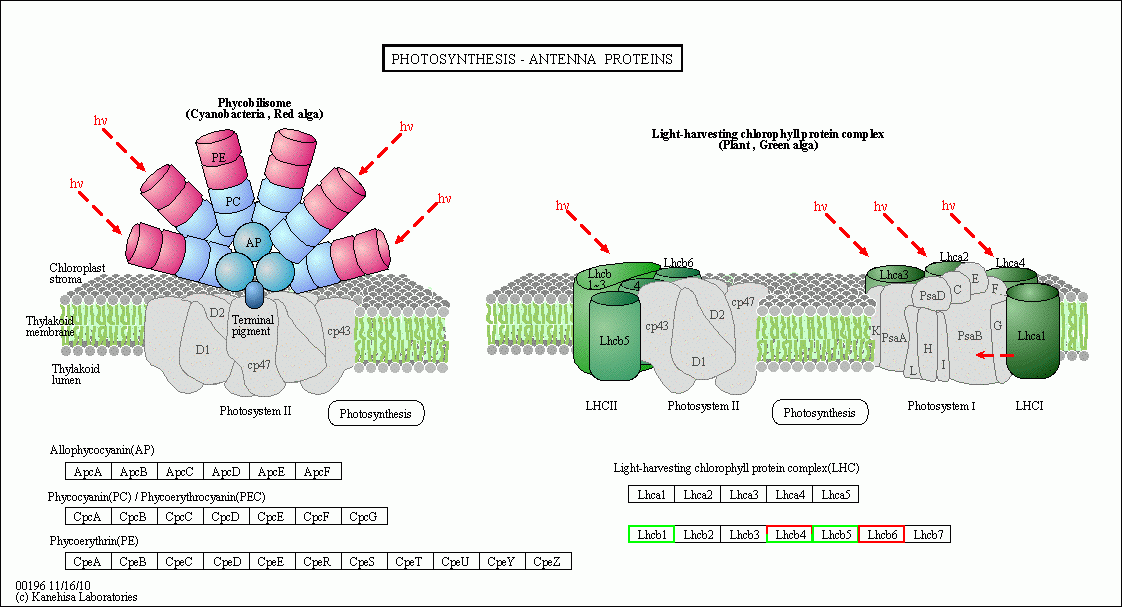

Supplement: Additional file 17: — KEGG analysis of differentially expressed genes between 72 h and 0 h (level3). [file 12864_2015_1373_MOESM17_ESM.zip › Additional file 5. KEGG analysis of differentially expressed genes between 72 h and 0 h (level3)/map00196.png]

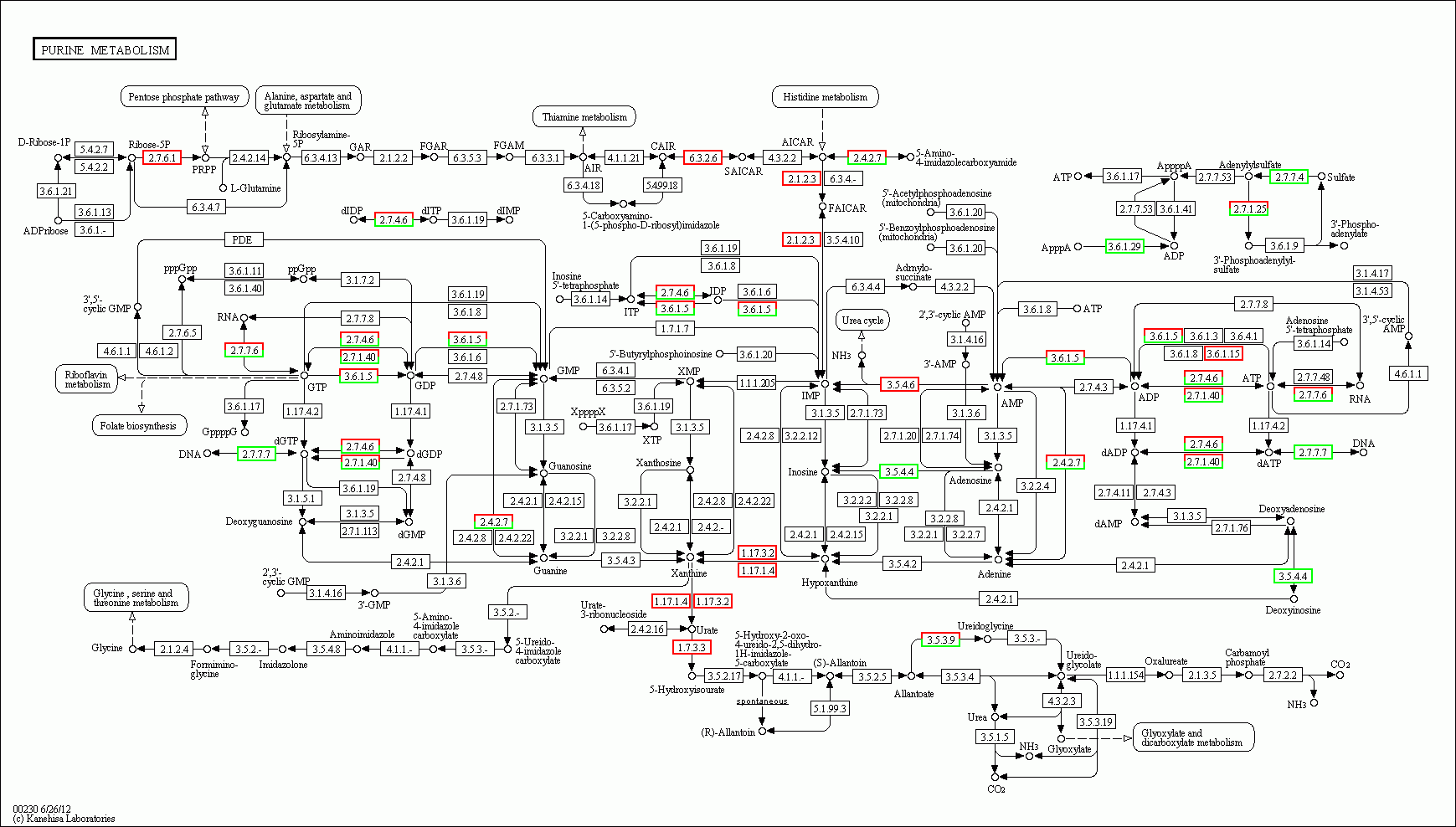

Supplement: Additional file 17: — KEGG analysis of differentially expressed genes between 72 h and 0 h (level3). [file 12864_2015_1373_MOESM17_ESM.zip › Additional file 5. KEGG analysis of differentially expressed genes between 72 h and 0 h (level3)/map00230.png]

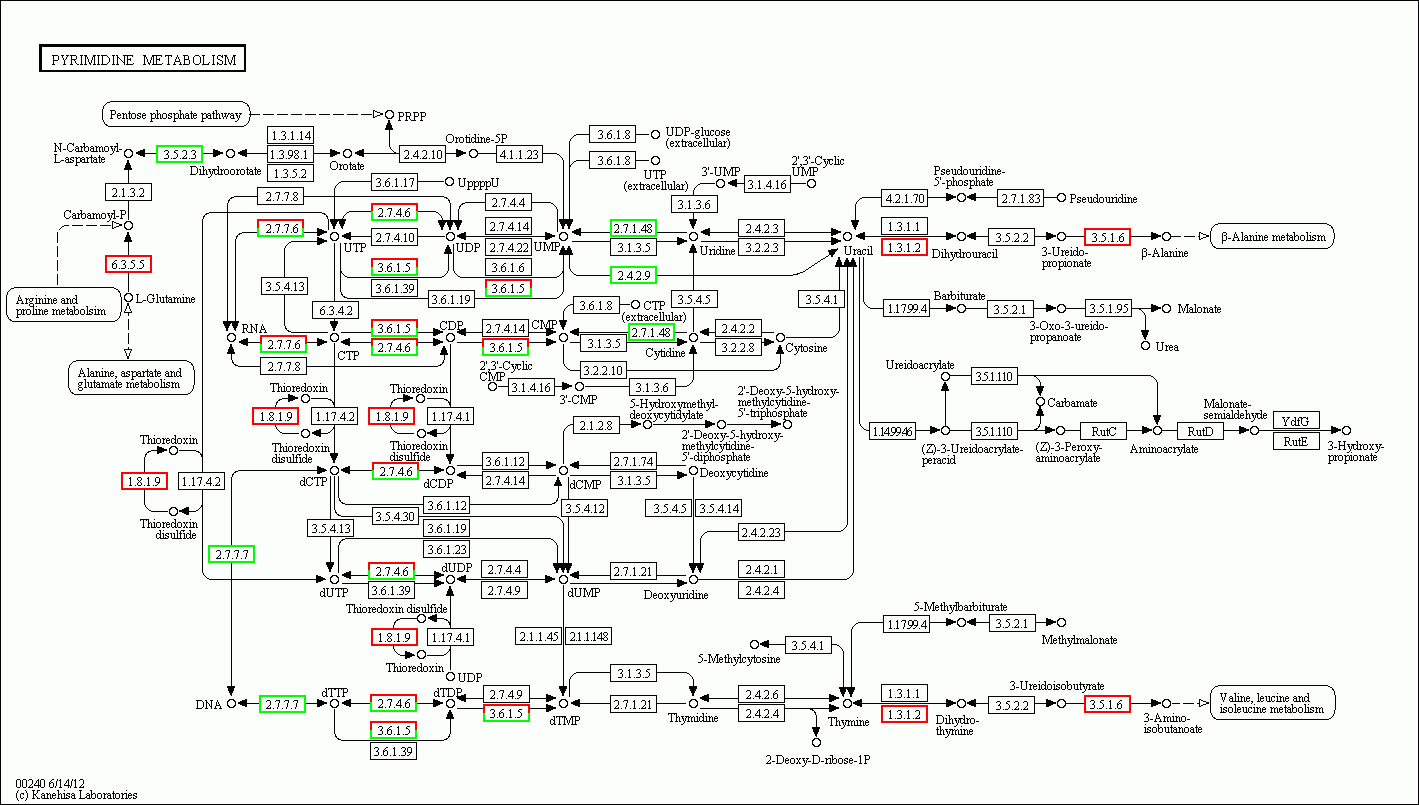

Supplement: Additional file 17: — KEGG analysis of differentially expressed genes between 72 h and 0 h (level3). [file 12864_2015_1373_MOESM17_ESM.zip › Additional file 5. KEGG analysis of differentially expressed genes between 72 h and 0 h (level3)/map00240.png]

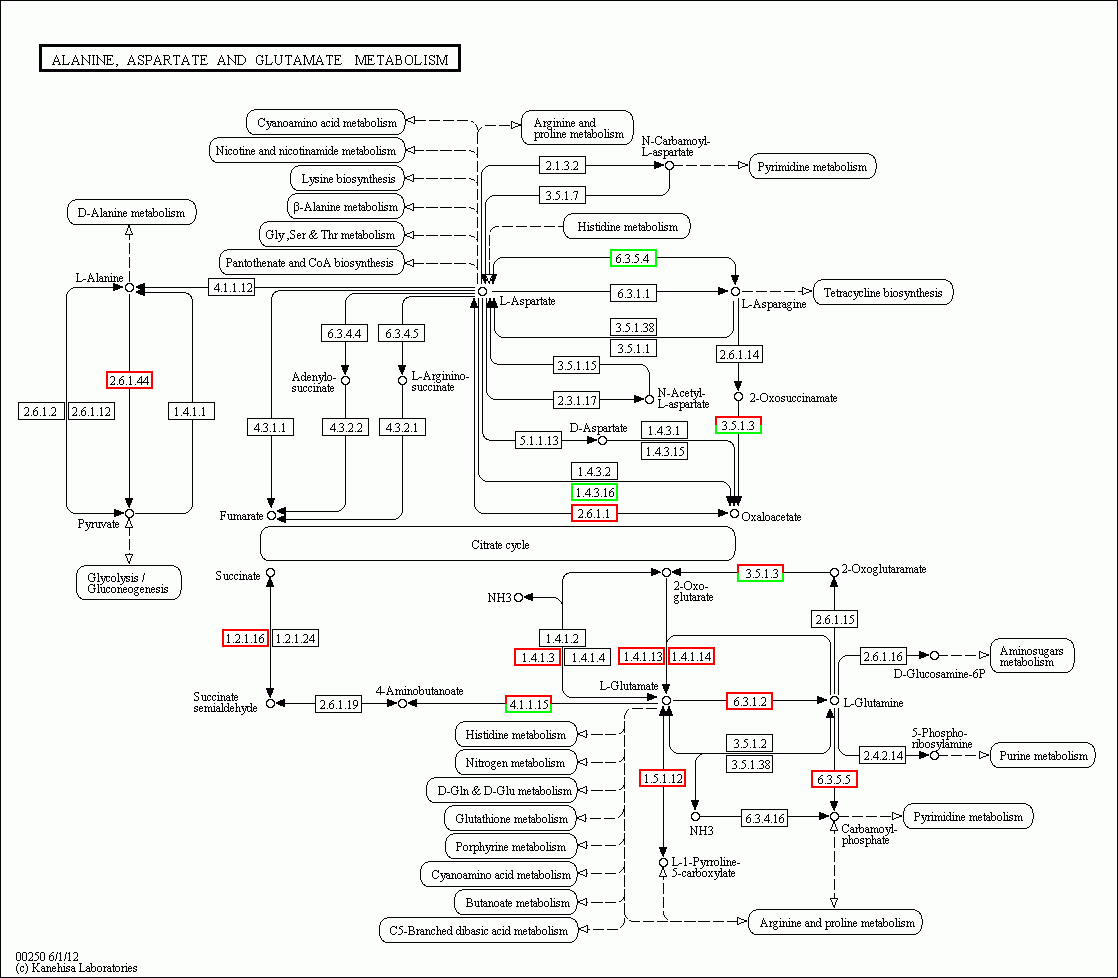

Supplement: Additional file 17: — KEGG analysis of differentially expressed genes between 72 h and 0 h (level3). [file 12864_2015_1373_MOESM17_ESM.zip › Additional file 5. KEGG analysis of differentially expressed genes between 72 h and 0 h (level3)/map00250.png]

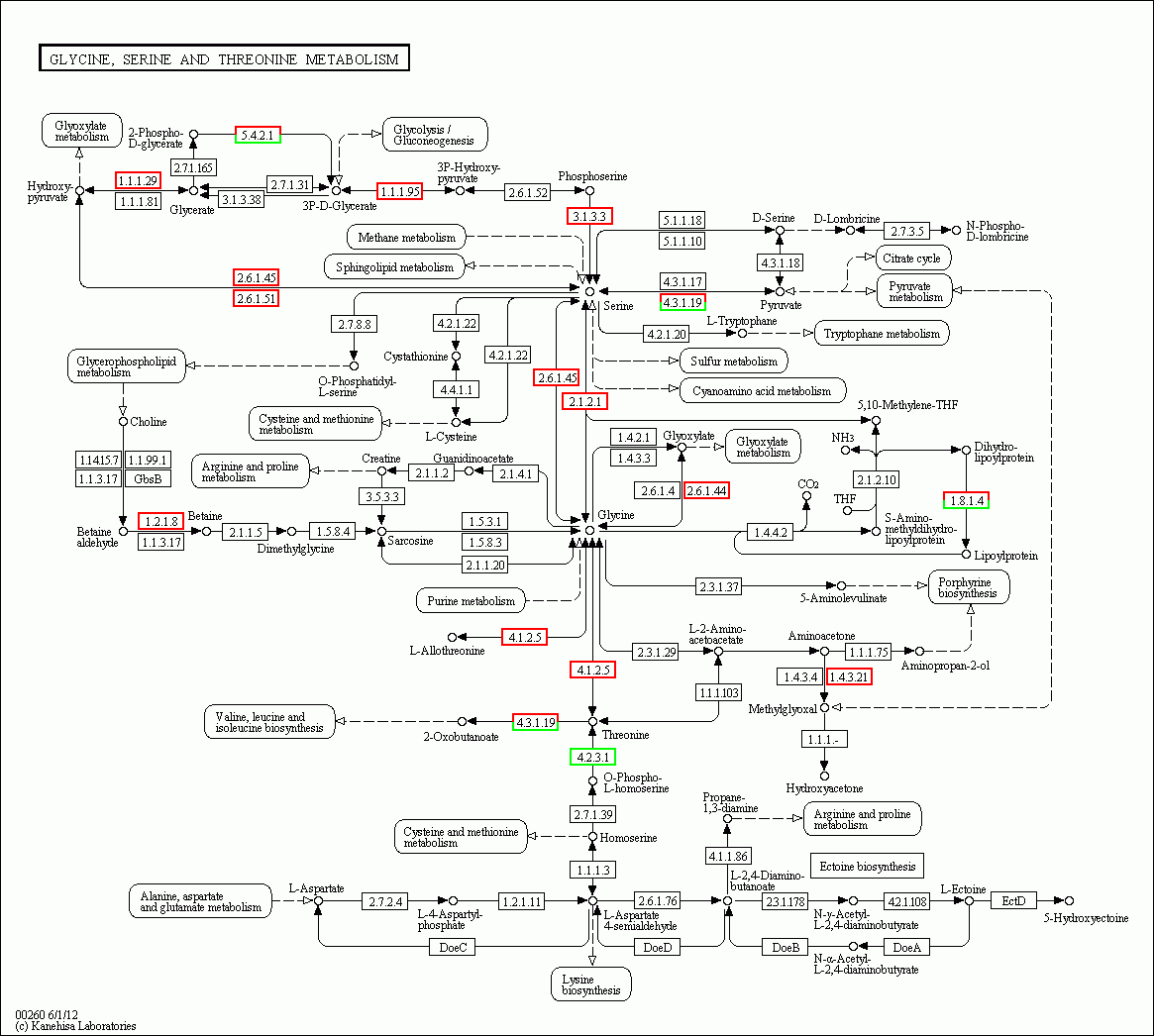

Supplement: Additional file 17: — KEGG analysis of differentially expressed genes between 72 h and 0 h (level3). [file 12864_2015_1373_MOESM17_ESM.zip › Additional file 5. KEGG analysis of differentially expressed genes between 72 h and 0 h (level3)/map00260.png]

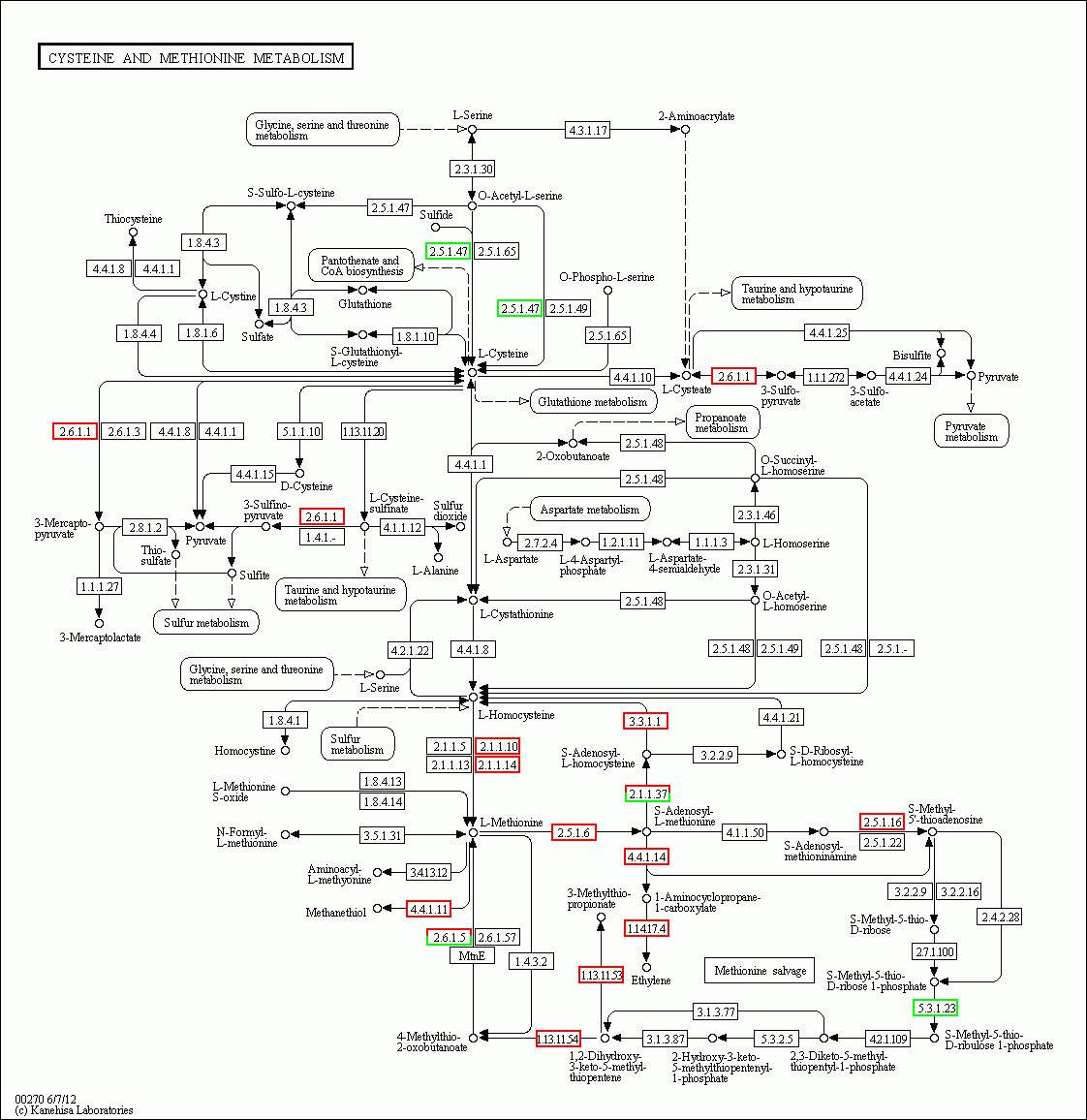

Supplement: Additional file 17: — KEGG analysis of differentially expressed genes between 72 h and 0 h (level3). [file 12864_2015_1373_MOESM17_ESM.zip › Additional file 5. KEGG analysis of differentially expressed genes between 72 h and 0 h (level3)/map00270.png]

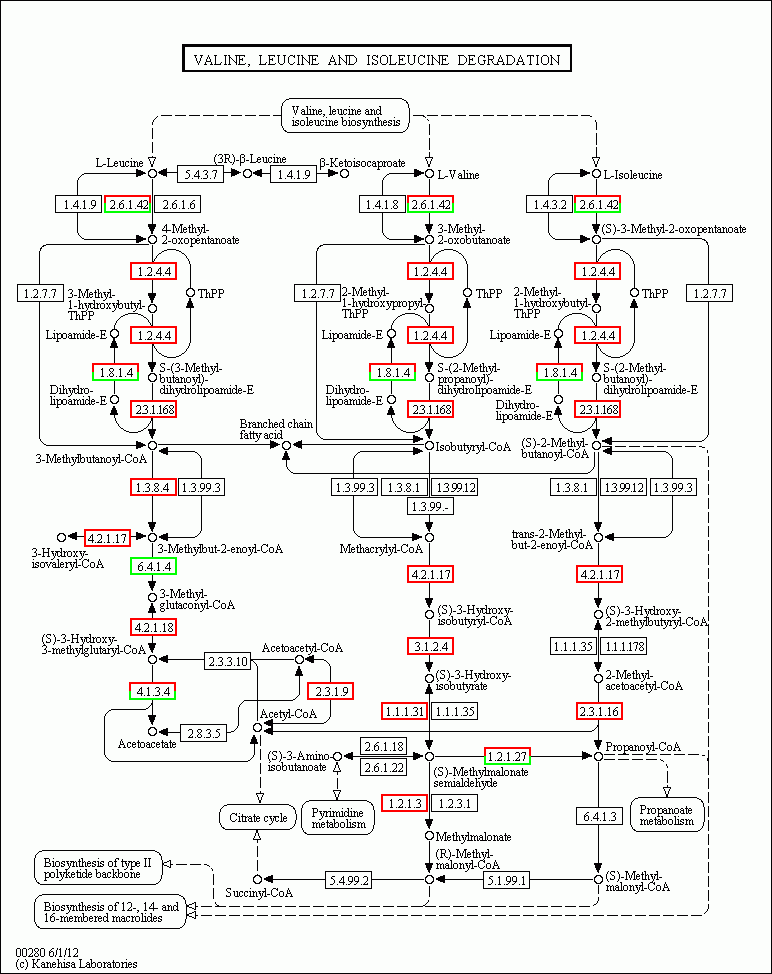

Supplement: Additional file 17: — KEGG analysis of differentially expressed genes between 72 h and 0 h (level3). [file 12864_2015_1373_MOESM17_ESM.zip › Additional file 5. KEGG analysis of differentially expressed genes between 72 h and 0 h (level3)/map00280.png]

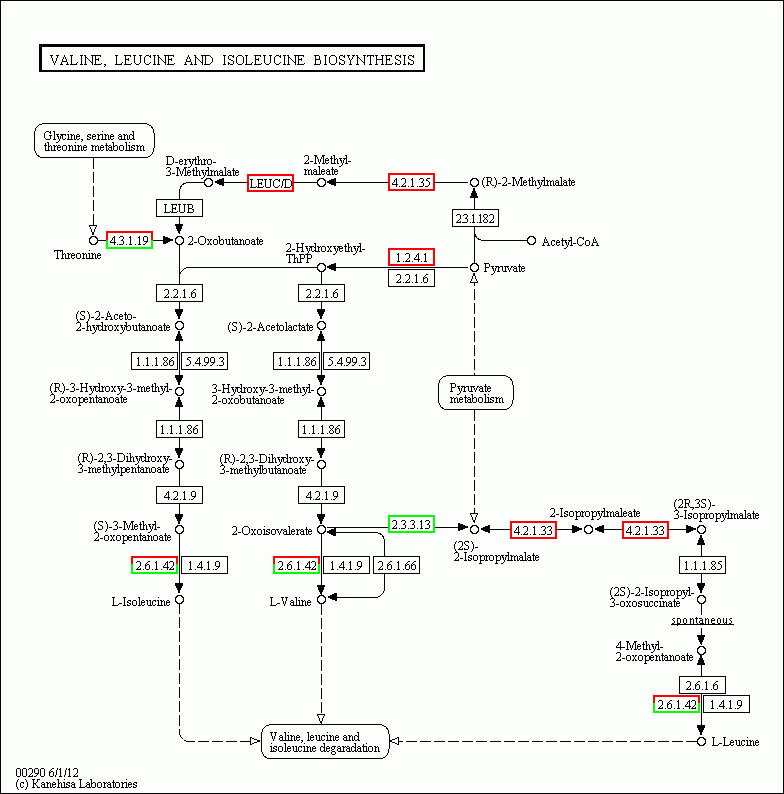

Supplement: Additional file 17: — KEGG analysis of differentially expressed genes between 72 h and 0 h (level3). [file 12864_2015_1373_MOESM17_ESM.zip › Additional file 5. KEGG analysis of differentially expressed genes between 72 h and 0 h (level3)/map00290.png]

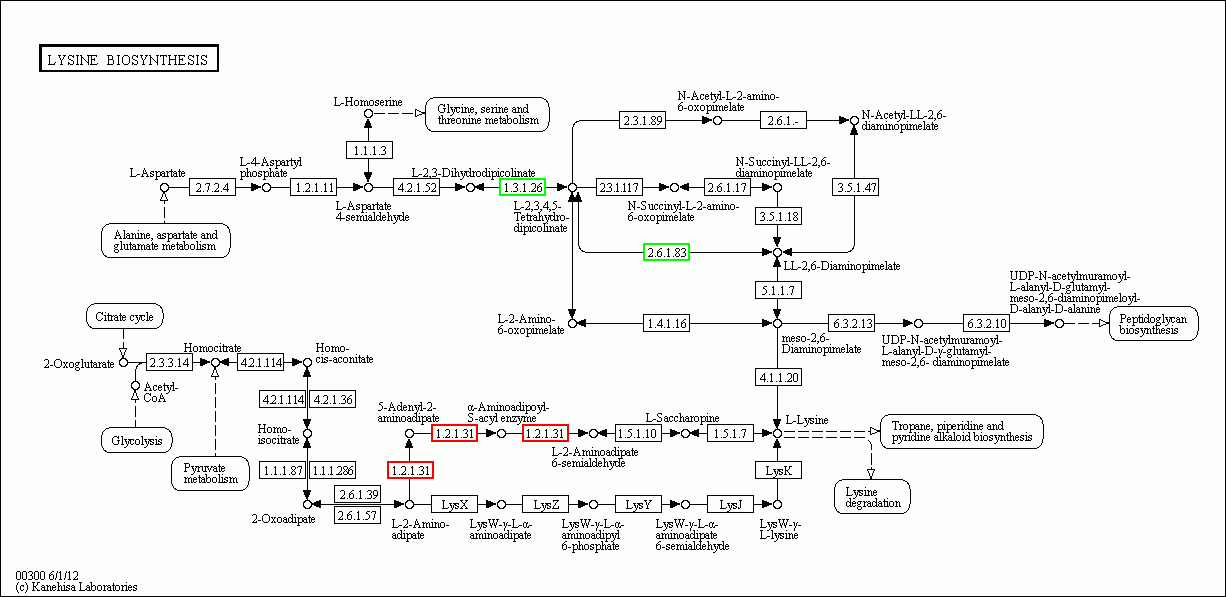

Supplement: Additional file 17: — KEGG analysis of differentially expressed genes between 72 h and 0 h (level3). [file 12864_2015_1373_MOESM17_ESM.zip › Additional file 5. KEGG analysis of differentially expressed genes between 72 h and 0 h (level3)/map00300.png]

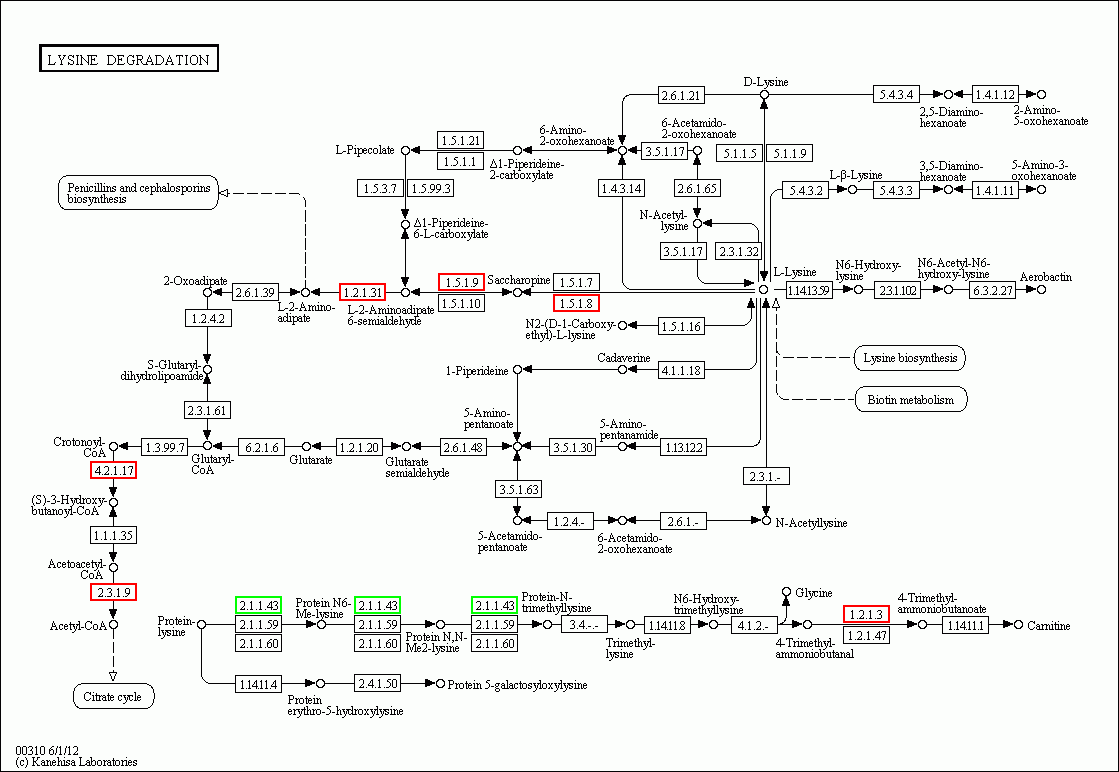

Supplement: Additional file 17: — KEGG analysis of differentially expressed genes between 72 h and 0 h (level3). [file 12864_2015_1373_MOESM17_ESM.zip › Additional file 5. KEGG analysis of differentially expressed genes between 72 h and 0 h (level3)/map00310.png]

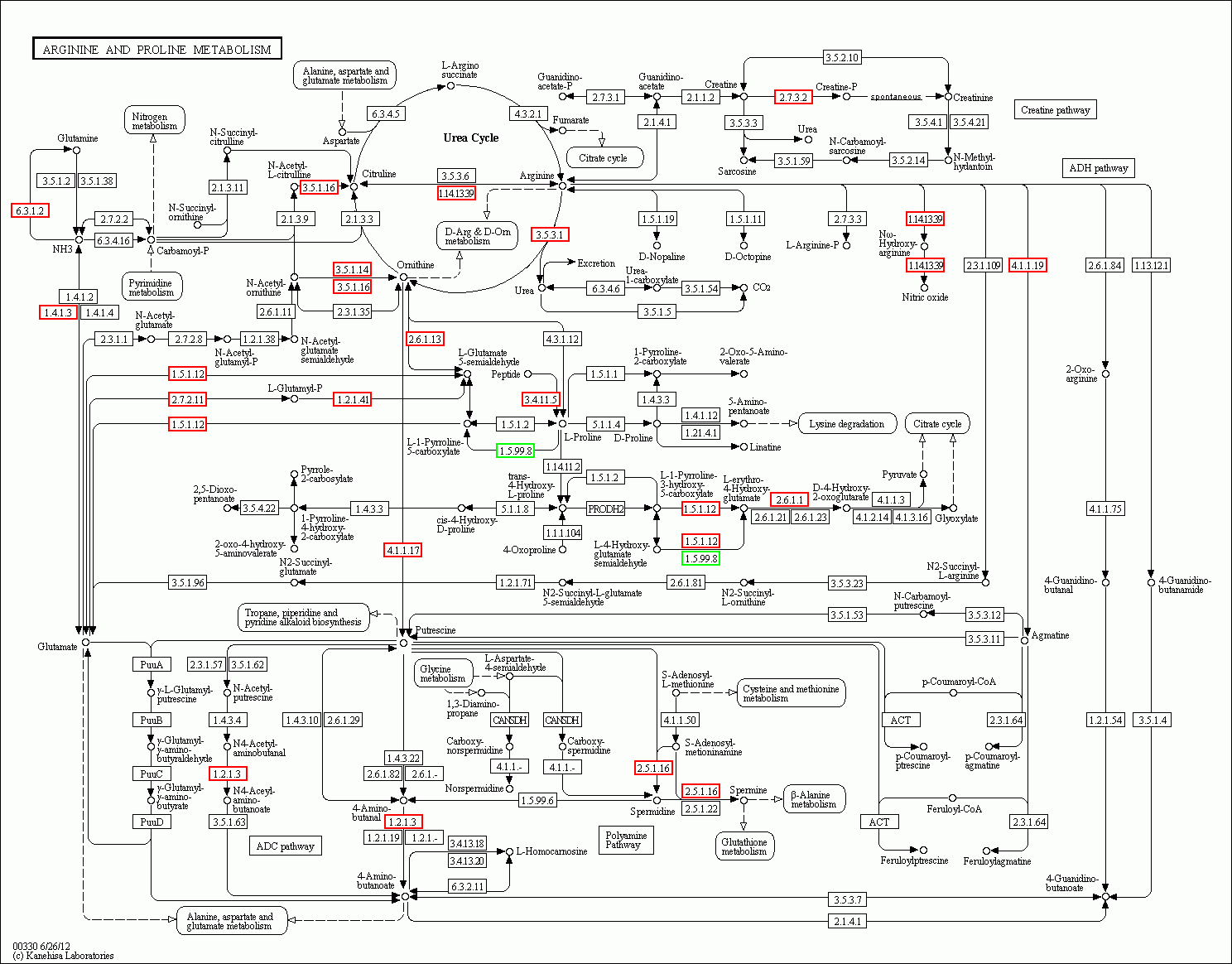

Supplement: Additional file 17: — KEGG analysis of differentially expressed genes between 72 h and 0 h (level3). [file 12864_2015_1373_MOESM17_ESM.zip › Additional file 5. KEGG analysis of differentially expressed genes between 72 h and 0 h (level3)/map00330.png]

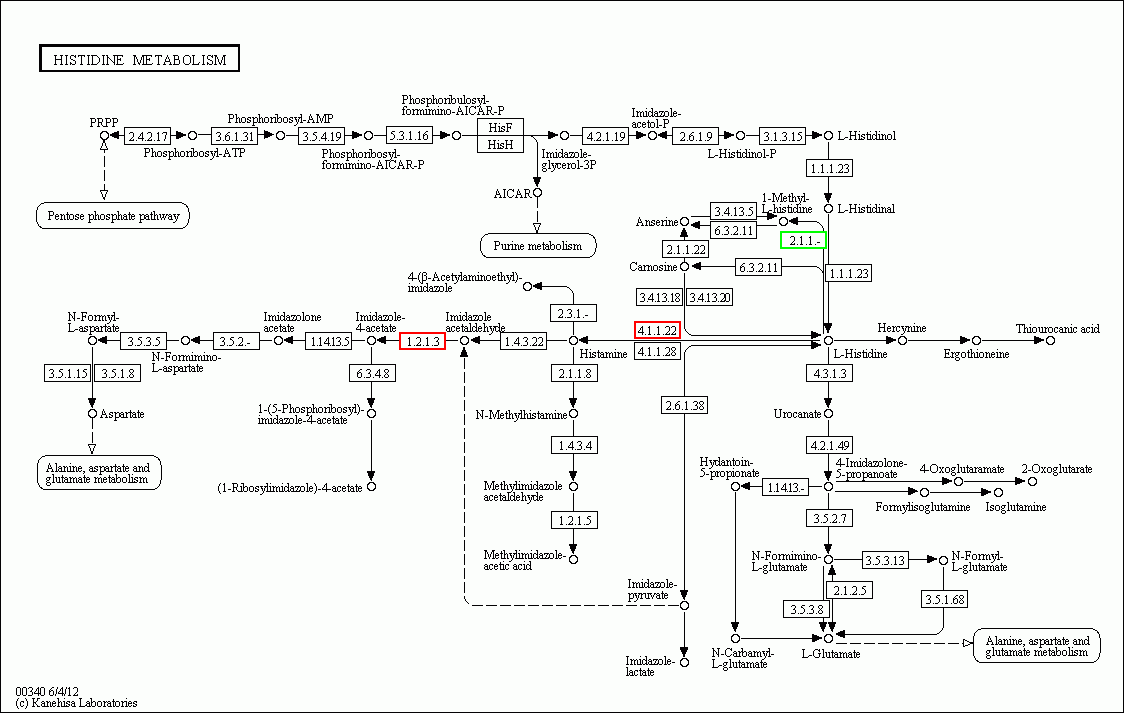

Supplement: Additional file 17: — KEGG analysis of differentially expressed genes between 72 h and 0 h (level3). [file 12864_2015_1373_MOESM17_ESM.zip › Additional file 5. KEGG analysis of differentially expressed genes between 72 h and 0 h (level3)/map00340.png]

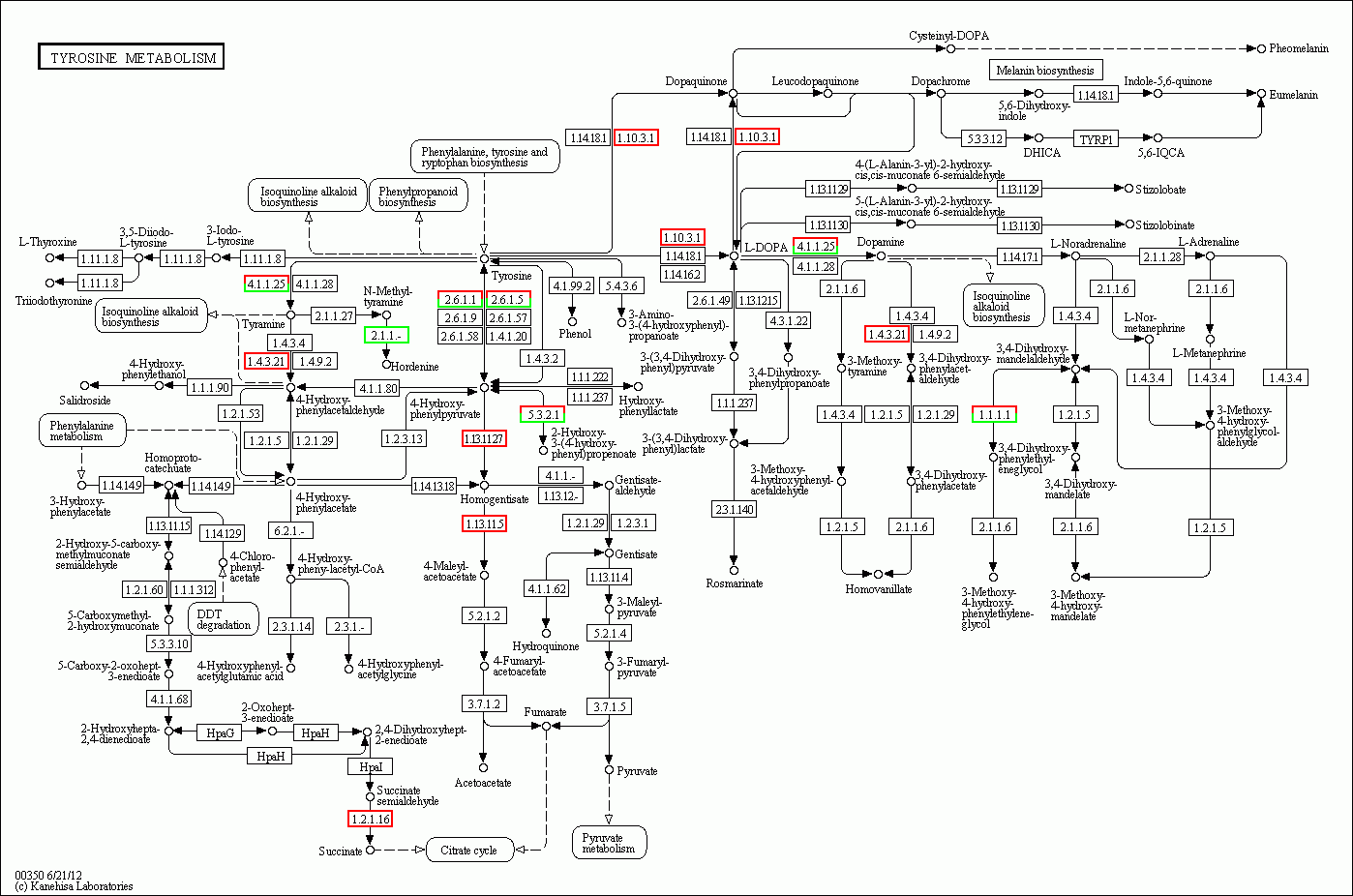

Supplement: Additional file 17: — KEGG analysis of differentially expressed genes between 72 h and 0 h (level3). [file 12864_2015_1373_MOESM17_ESM.zip › Additional file 5. KEGG analysis of differentially expressed genes between 72 h and 0 h (level3)/map00350.png]

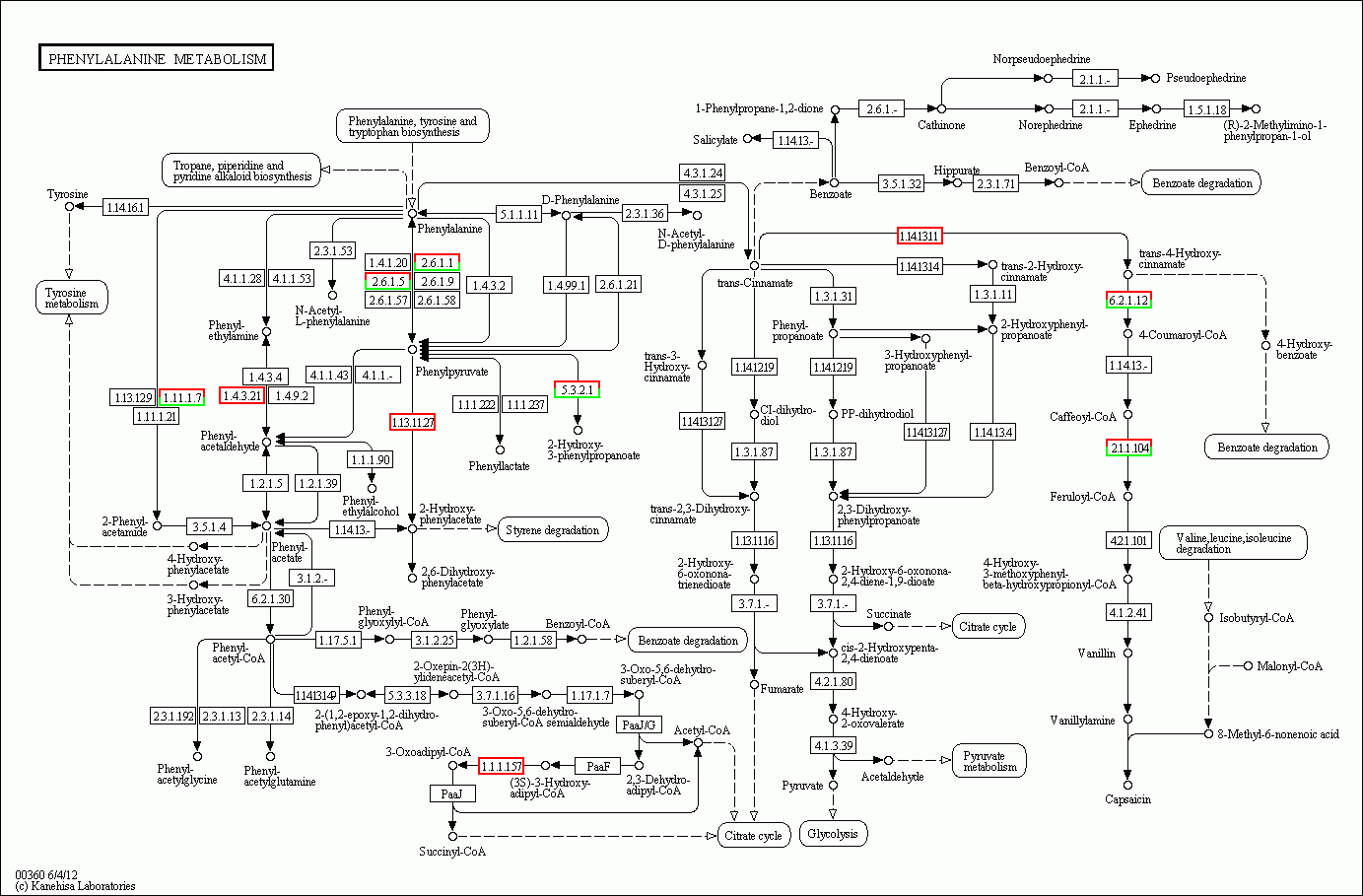

Supplement: Additional file 17: — KEGG analysis of differentially expressed genes between 72 h and 0 h (level3). [file 12864_2015_1373_MOESM17_ESM.zip › Additional file 5. KEGG analysis of differentially expressed genes between 72 h and 0 h (level3)/map00360.png]

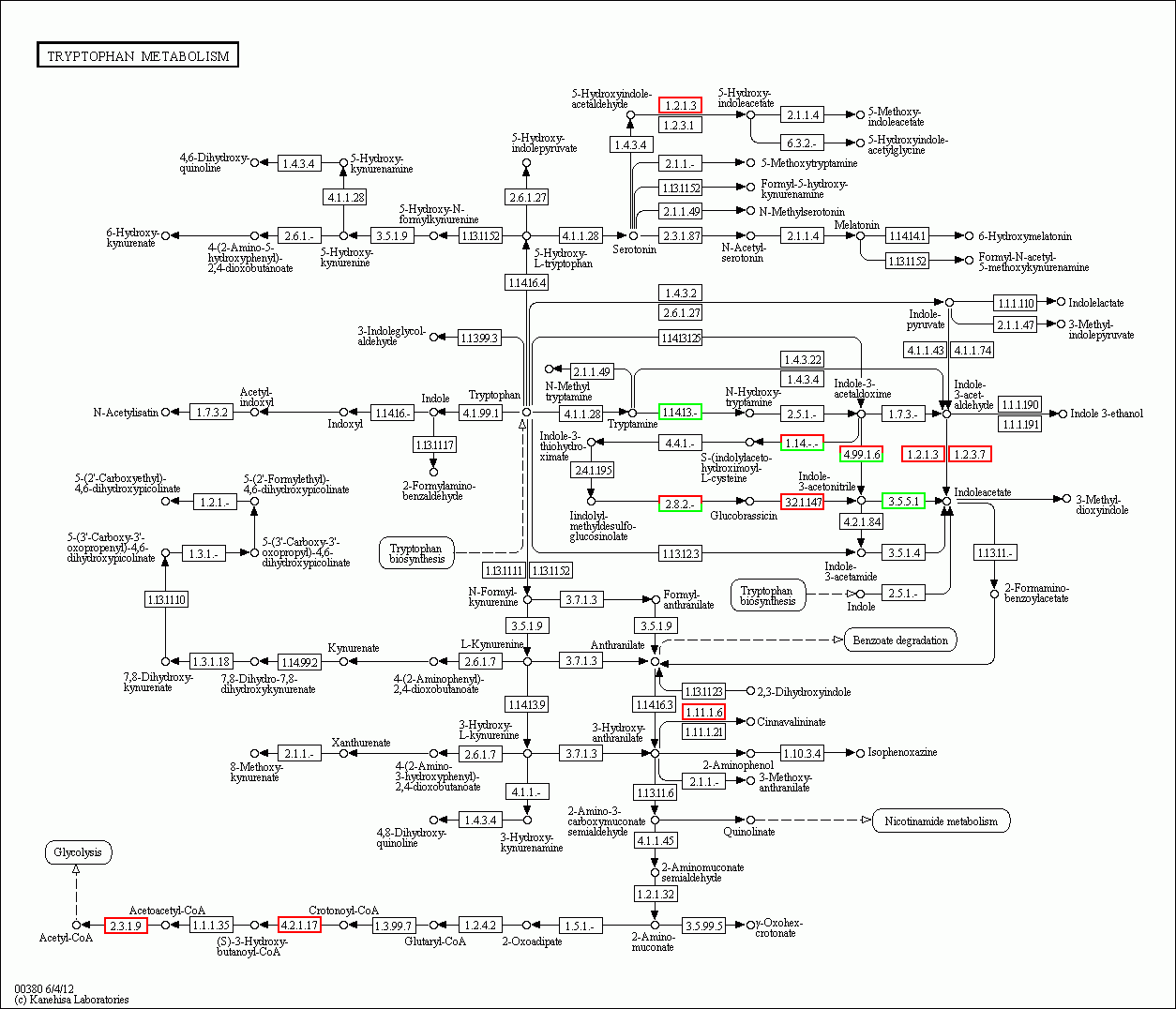

Supplement: Additional file 17: — KEGG analysis of differentially expressed genes between 72 h and 0 h (level3). [file 12864_2015_1373_MOESM17_ESM.zip › Additional file 5. KEGG analysis of differentially expressed genes between 72 h and 0 h (level3)/map00380.png]

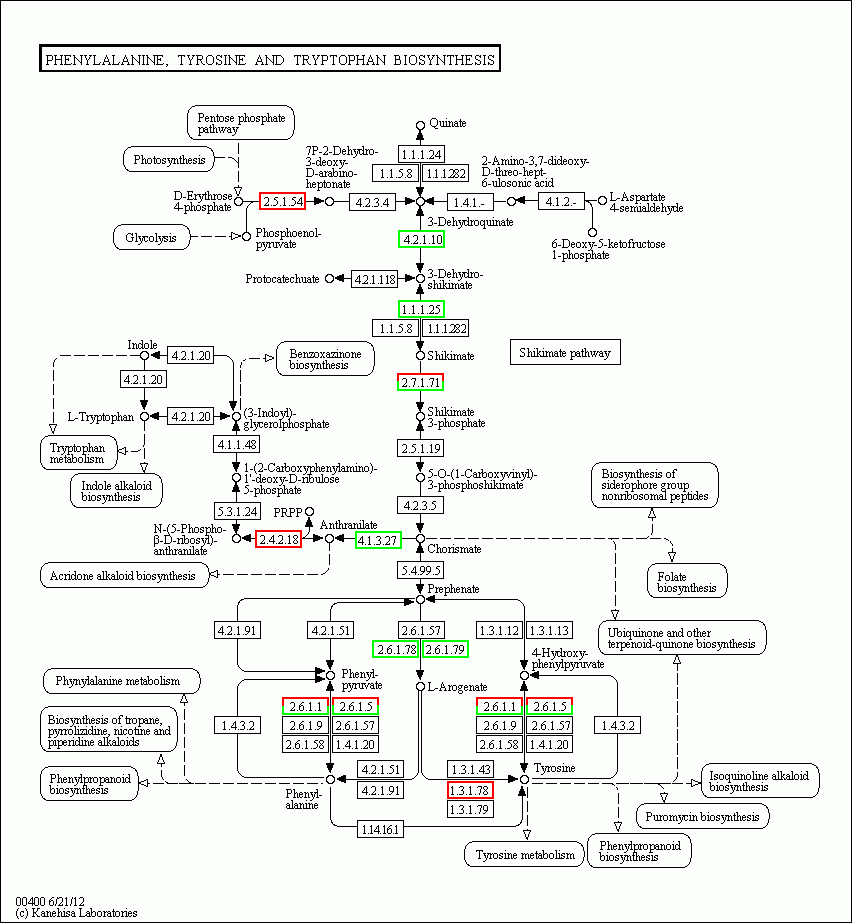

Supplement: Additional file 17: — KEGG analysis of differentially expressed genes between 72 h and 0 h (level3). [file 12864_2015_1373_MOESM17_ESM.zip › Additional file 5. KEGG analysis of differentially expressed genes between 72 h and 0 h (level3)/map00400.png]

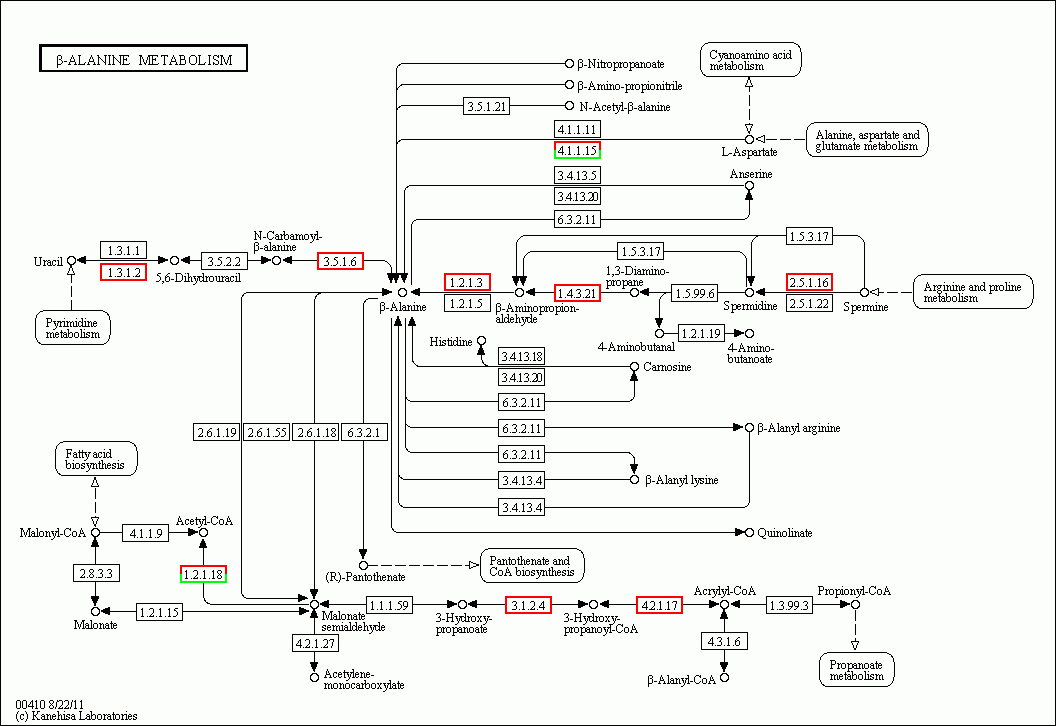

Supplement: Additional file 17: — KEGG analysis of differentially expressed genes between 72 h and 0 h (level3). [file 12864_2015_1373_MOESM17_ESM.zip › Additional file 5. KEGG analysis of differentially expressed genes between 72 h and 0 h (level3)/map00410.png]

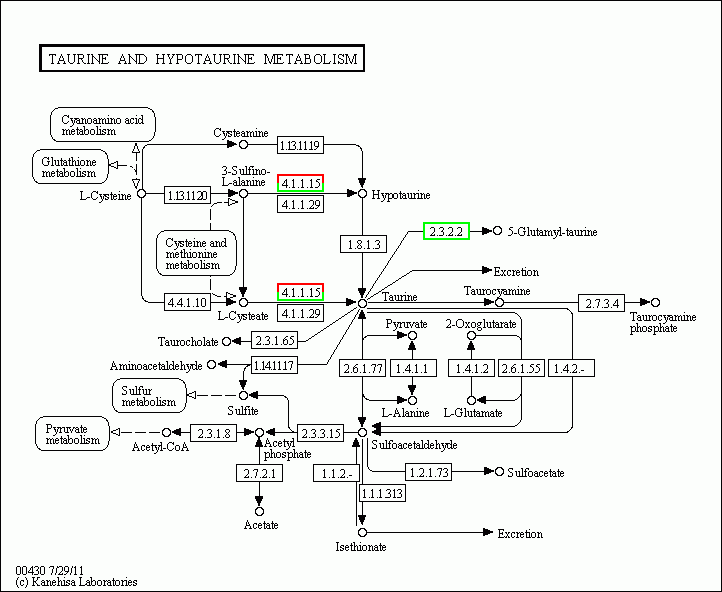

Supplement: Additional file 17: — KEGG analysis of differentially expressed genes between 72 h and 0 h (level3). [file 12864_2015_1373_MOESM17_ESM.zip › Additional file 5. KEGG analysis of differentially expressed genes between 72 h and 0 h (level3)/map00430.png]

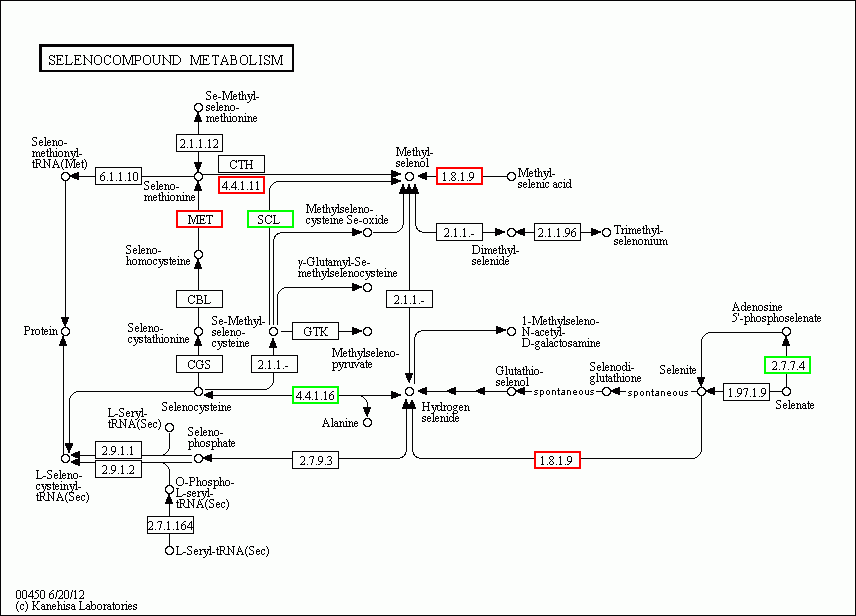

Supplement: Additional file 17: — KEGG analysis of differentially expressed genes between 72 h and 0 h (level3). [file 12864_2015_1373_MOESM17_ESM.zip › Additional file 5. KEGG analysis of differentially expressed genes between 72 h and 0 h (level3)/map00450.png]

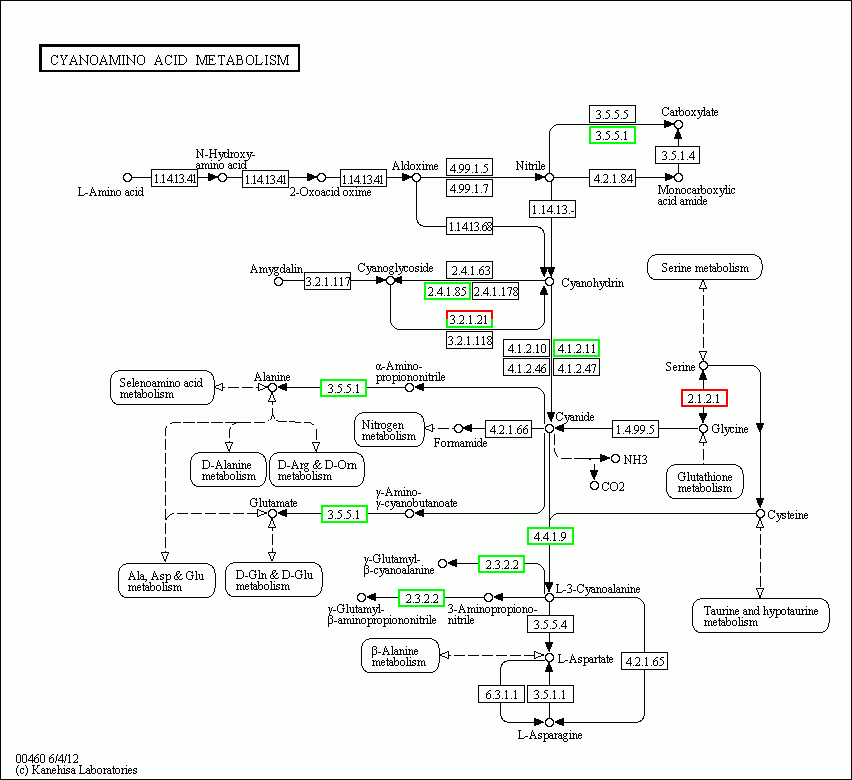

Supplement: Additional file 17: — KEGG analysis of differentially expressed genes between 72 h and 0 h (level3). [file 12864_2015_1373_MOESM17_ESM.zip › Additional file 5. KEGG analysis of differentially expressed genes between 72 h and 0 h (level3)/map00460.png]

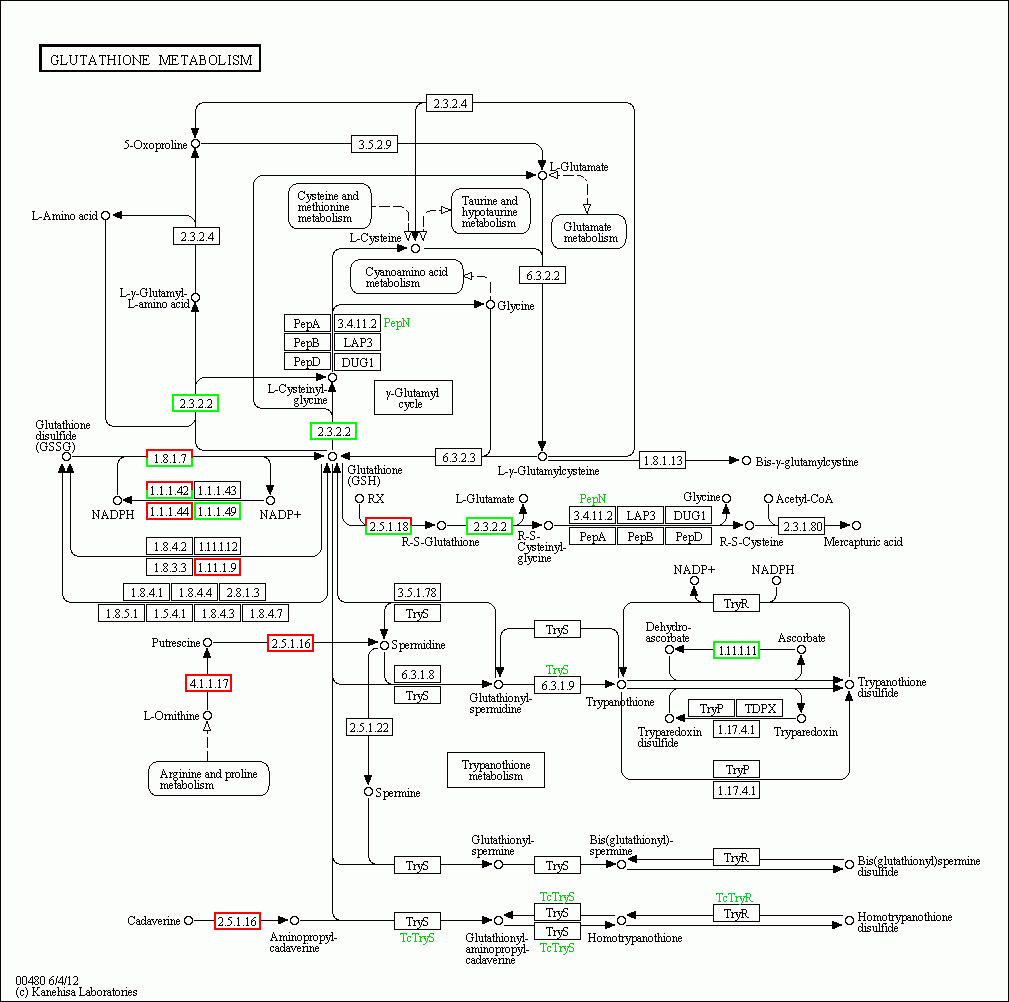

Supplement: Additional file 17: — KEGG analysis of differentially expressed genes between 72 h and 0 h (level3). [file 12864_2015_1373_MOESM17_ESM.zip › Additional file 5. KEGG analysis of differentially expressed genes between 72 h and 0 h (level3)/map00480.png]

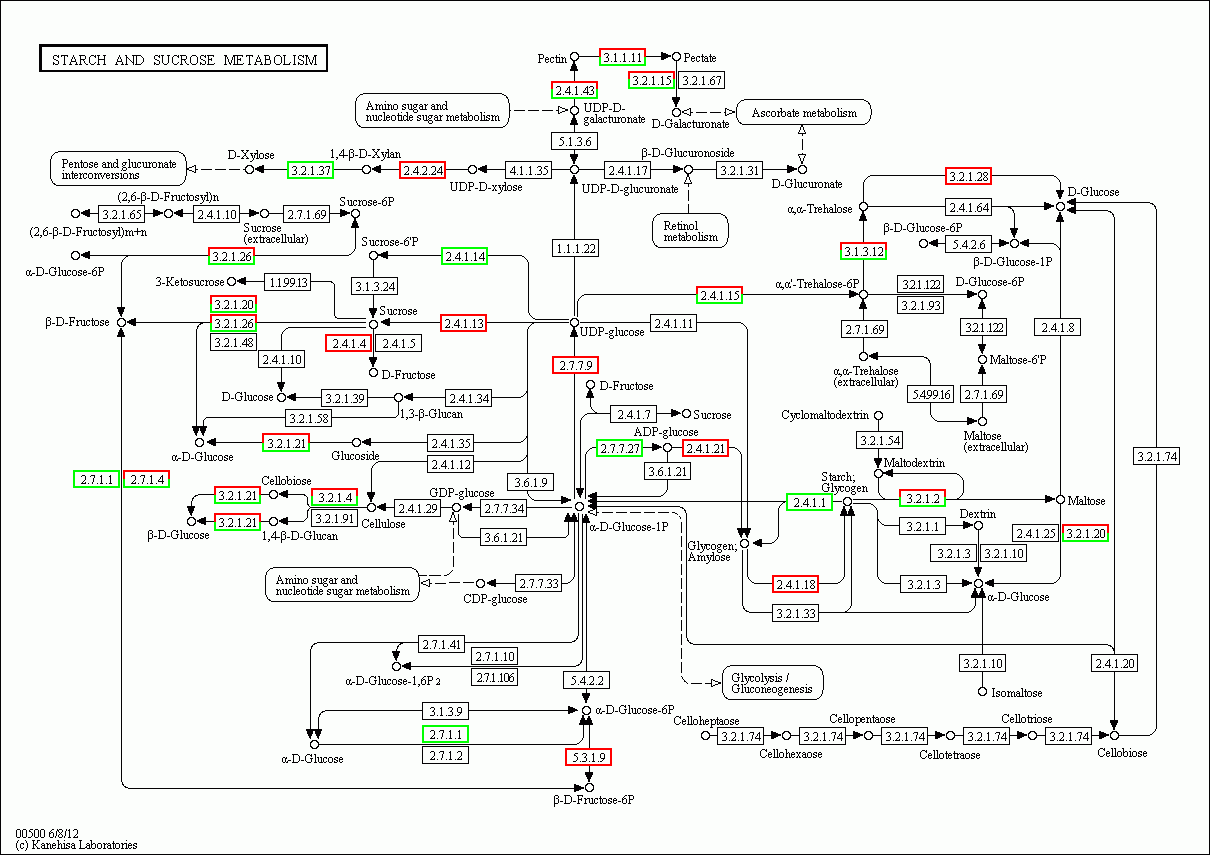

Supplement: Additional file 17: — KEGG analysis of differentially expressed genes between 72 h and 0 h (level3). [file 12864_2015_1373_MOESM17_ESM.zip › Additional file 5. KEGG analysis of differentially expressed genes between 72 h and 0 h (level3)/map00500.png]

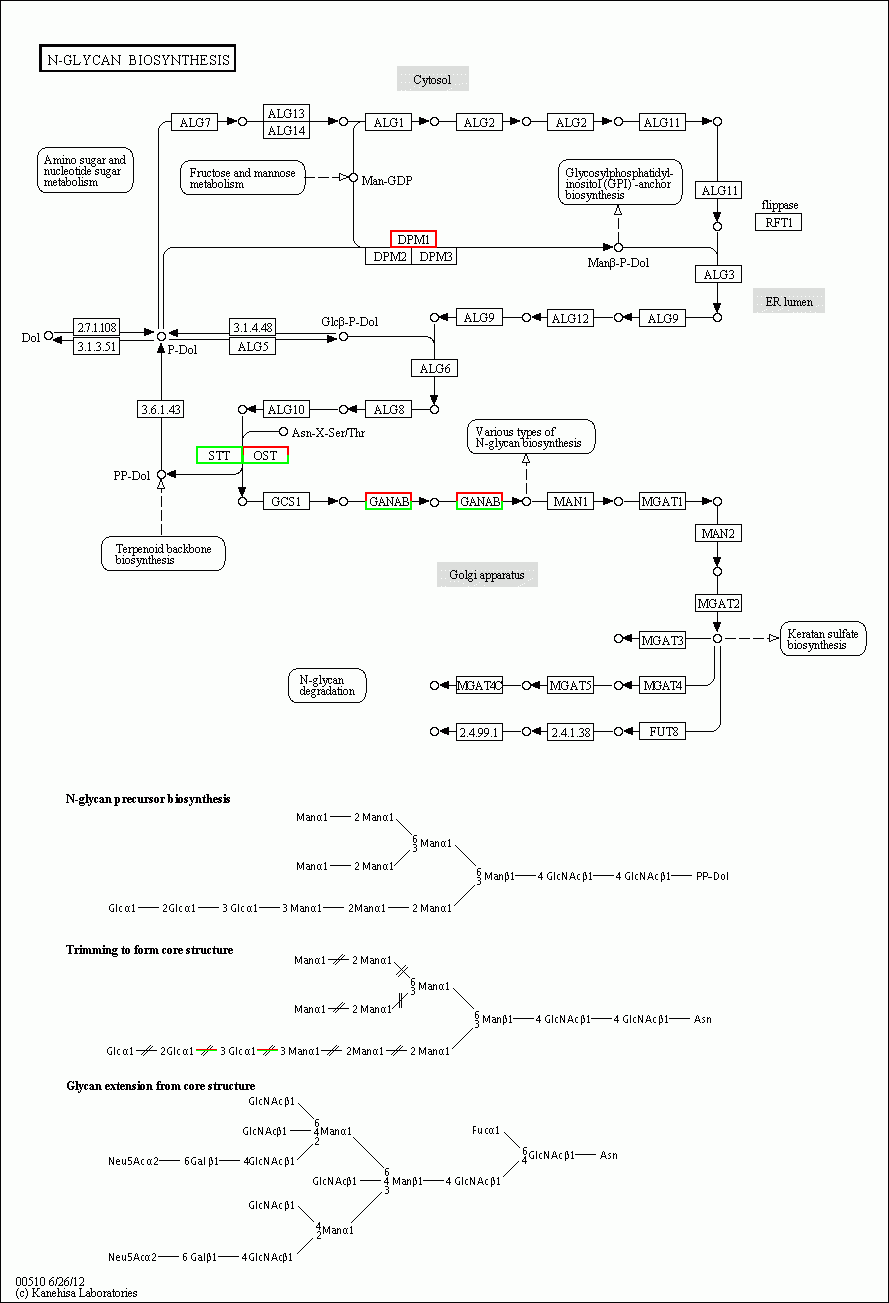

Supplement: Additional file 17: — KEGG analysis of differentially expressed genes between 72 h and 0 h (level3). [file 12864_2015_1373_MOESM17_ESM.zip › Additional file 5. KEGG analysis of differentially expressed genes between 72 h and 0 h (level3)/map00510.png]

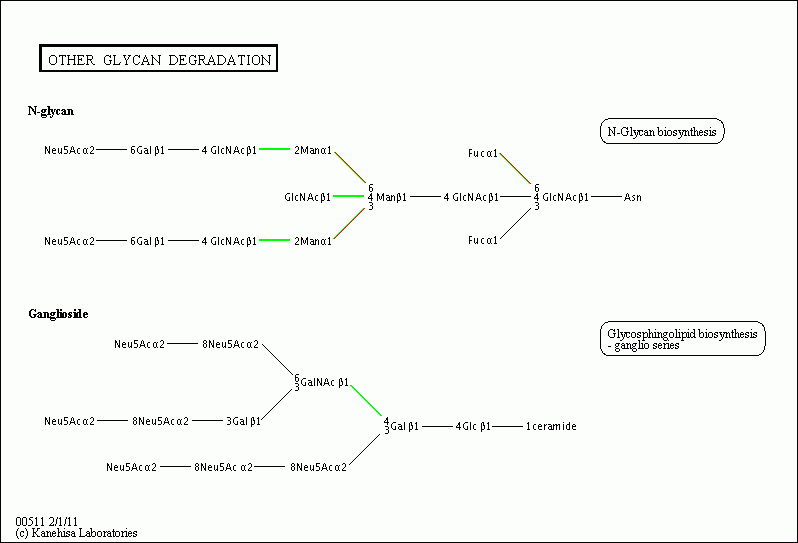

Supplement: Additional file 17: — KEGG analysis of differentially expressed genes between 72 h and 0 h (level3). [file 12864_2015_1373_MOESM17_ESM.zip › Additional file 5. KEGG analysis of differentially expressed genes between 72 h and 0 h (level3)/map00511.png]

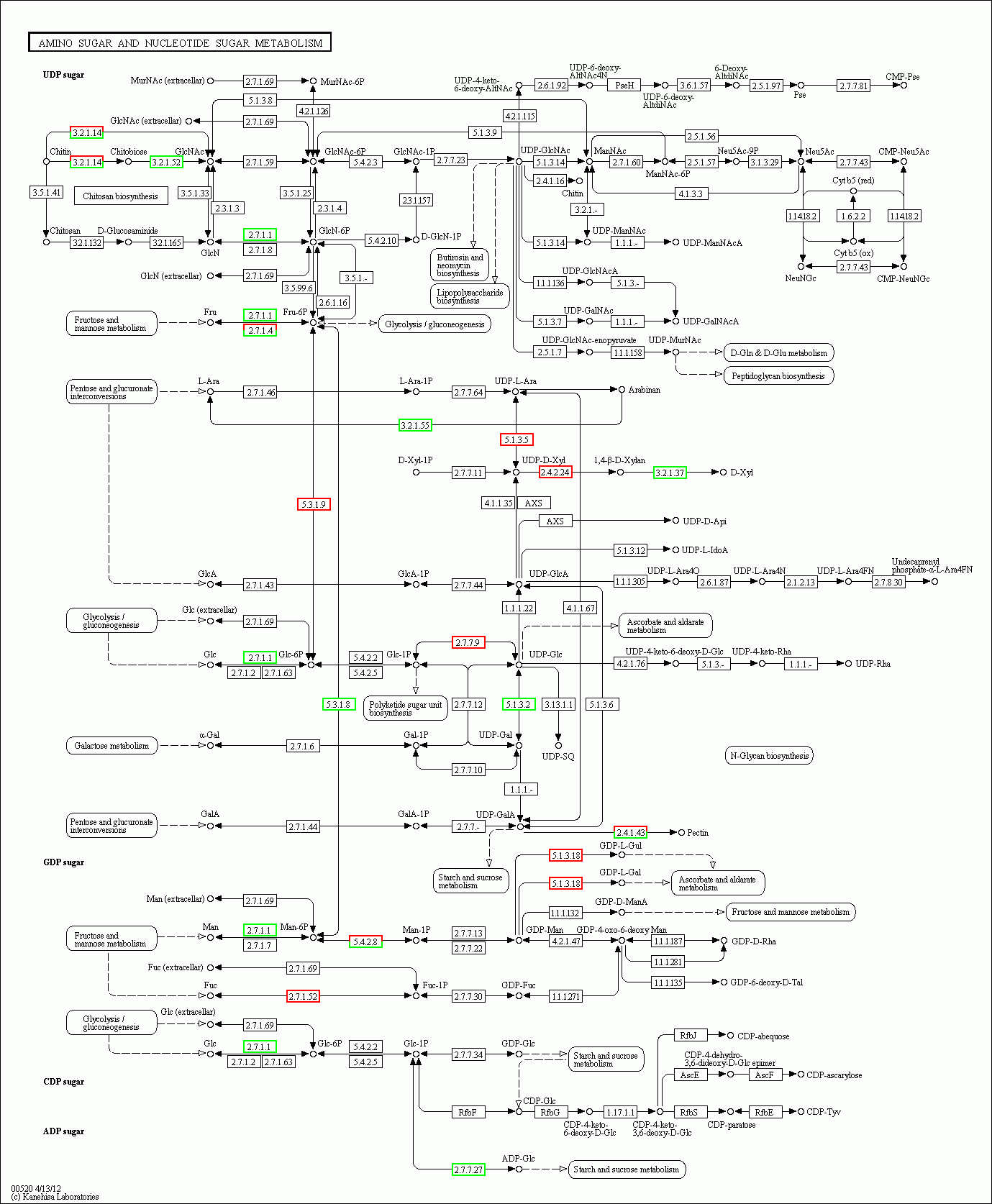

Supplement: Additional file 17: — KEGG analysis of differentially expressed genes between 72 h and 0 h (level3). [file 12864_2015_1373_MOESM17_ESM.zip › Additional file 5. KEGG analysis of differentially expressed genes between 72 h and 0 h (level3)/map00520.png]

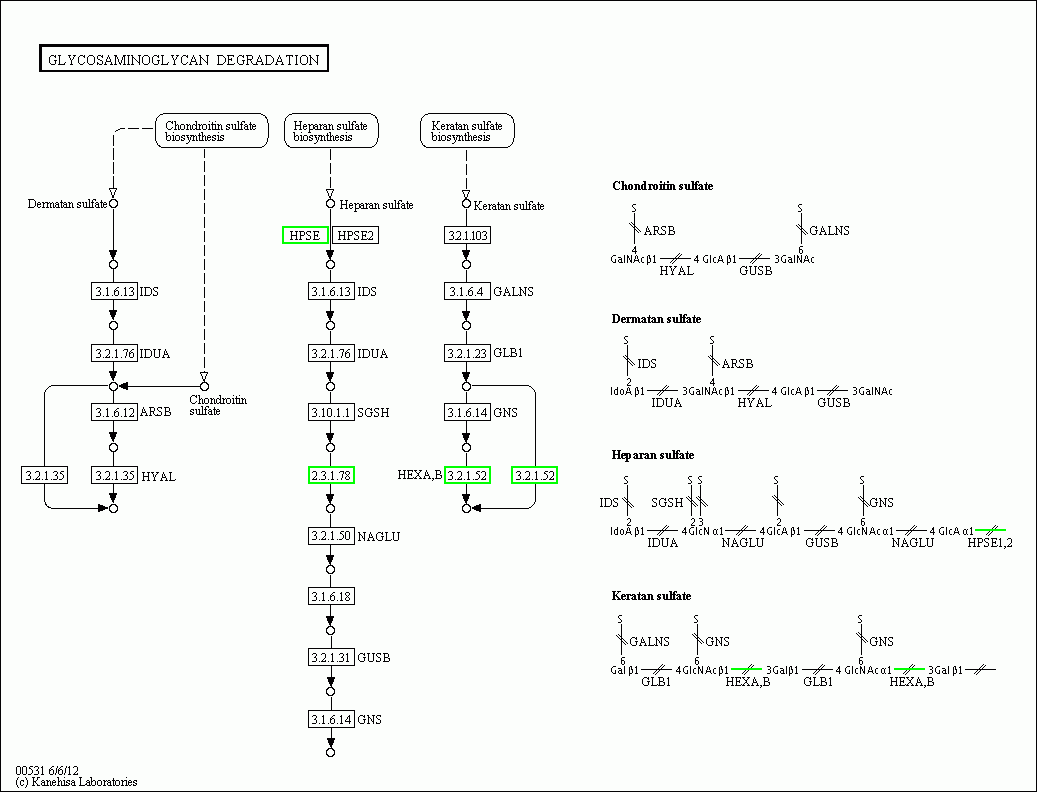

Supplement: Additional file 17: — KEGG analysis of differentially expressed genes between 72 h and 0 h (level3). [file 12864_2015_1373_MOESM17_ESM.zip › Additional file 5. KEGG analysis of differentially expressed genes between 72 h and 0 h (level3)/map00531.png]

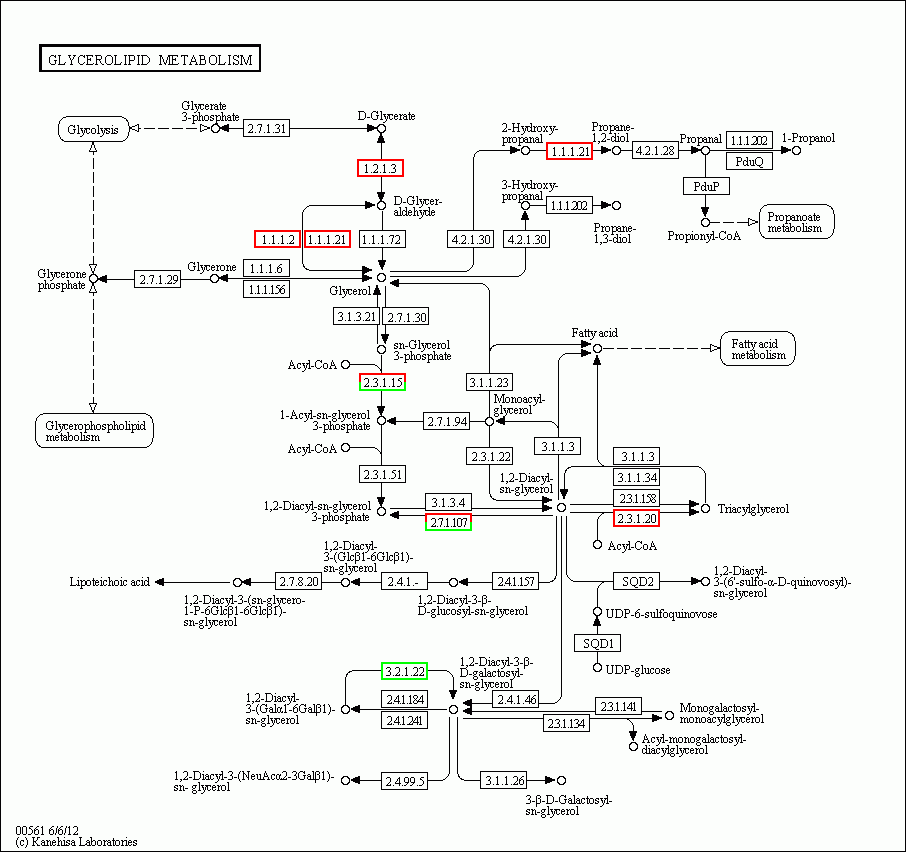

Supplement: Additional file 17: — KEGG analysis of differentially expressed genes between 72 h and 0 h (level3). [file 12864_2015_1373_MOESM17_ESM.zip › Additional file 5. KEGG analysis of differentially expressed genes between 72 h and 0 h (level3)/map00561.png]

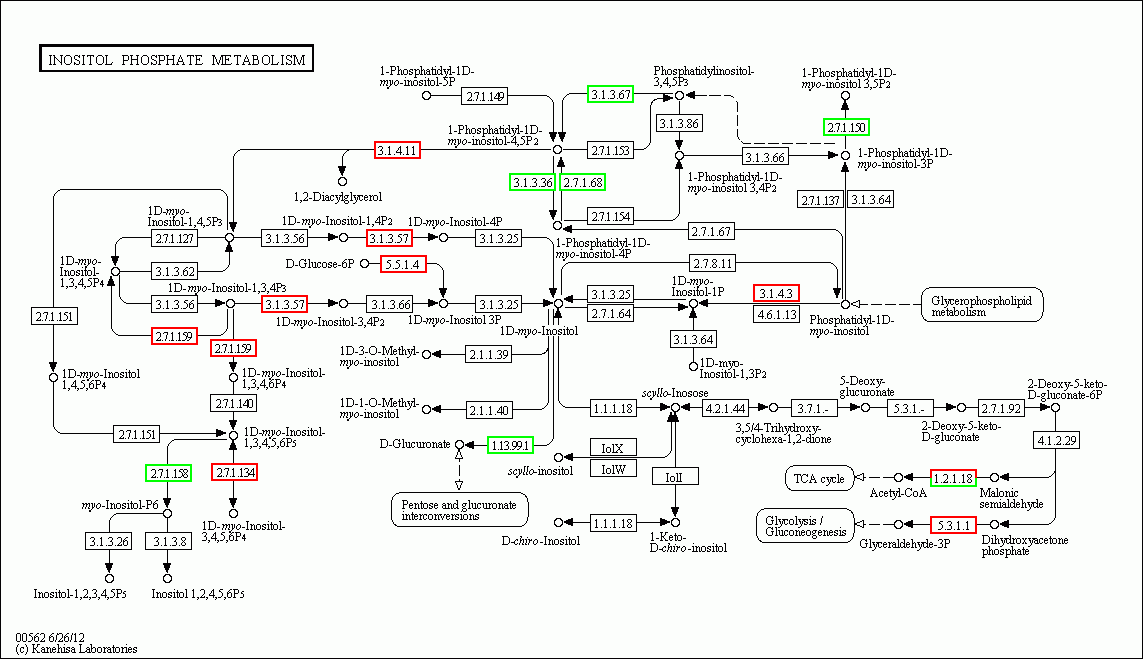

Supplement: Additional file 17: — KEGG analysis of differentially expressed genes between 72 h and 0 h (level3). [file 12864_2015_1373_MOESM17_ESM.zip › Additional file 5. KEGG analysis of differentially expressed genes between 72 h and 0 h (level3)/map00562.png]

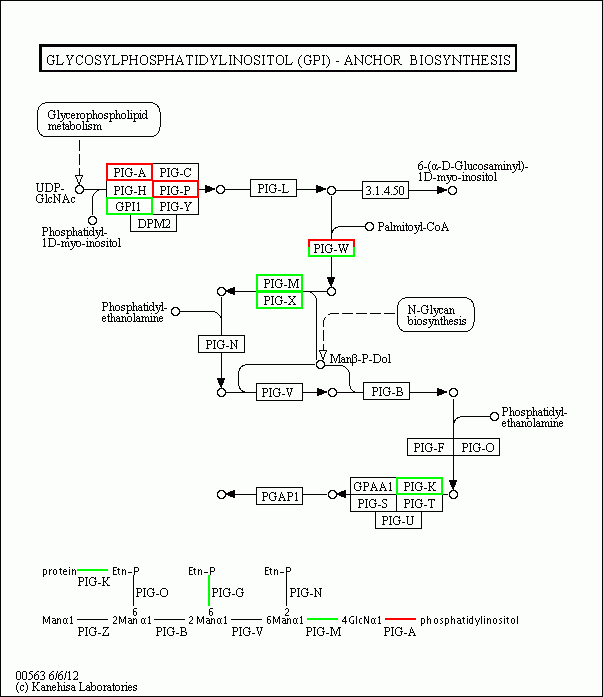

Supplement: Additional file 17: — KEGG analysis of differentially expressed genes between 72 h and 0 h (level3). [file 12864_2015_1373_MOESM17_ESM.zip › Additional file 5. KEGG analysis of differentially expressed genes between 72 h and 0 h (level3)/map00563.png]

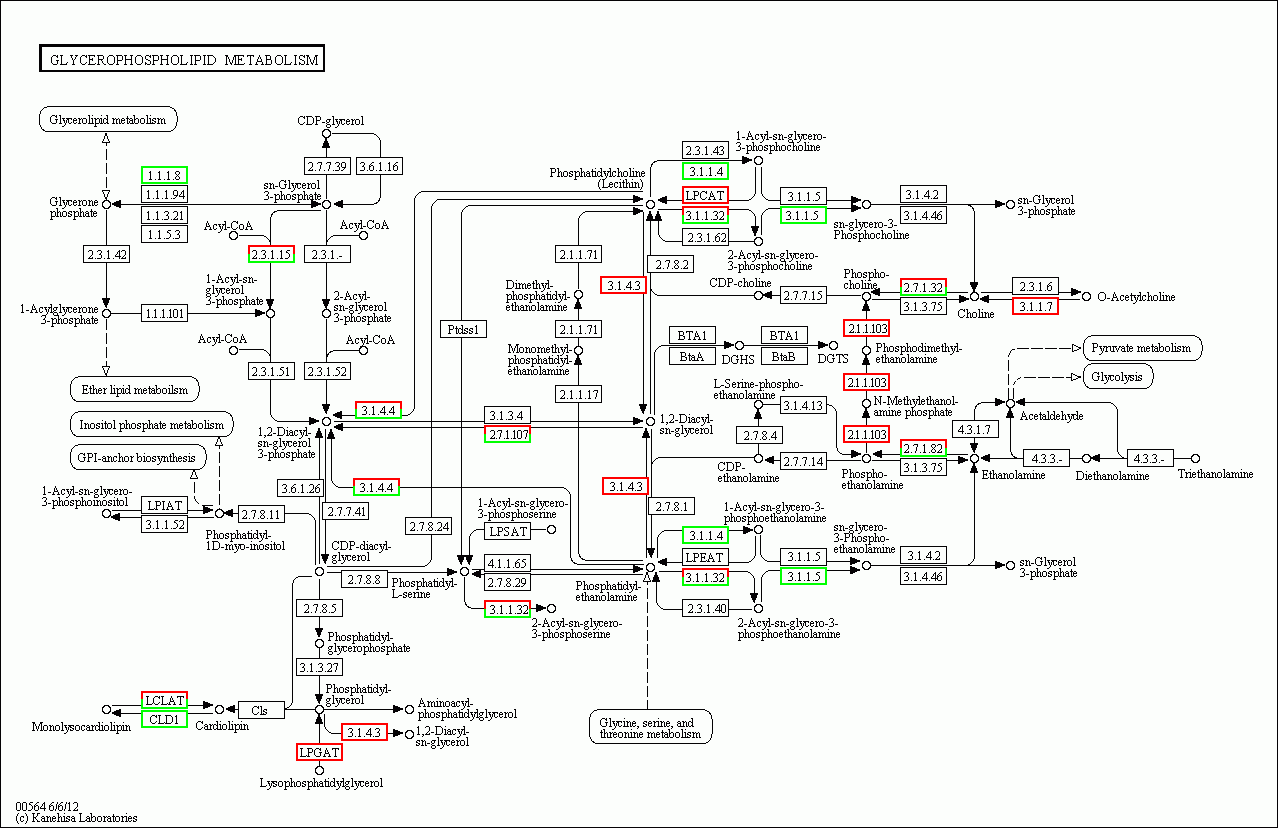

Supplement: Additional file 17: — KEGG analysis of differentially expressed genes between 72 h and 0 h (level3). [file 12864_2015_1373_MOESM17_ESM.zip › Additional file 5. KEGG analysis of differentially expressed genes between 72 h and 0 h (level3)/map00564.png]

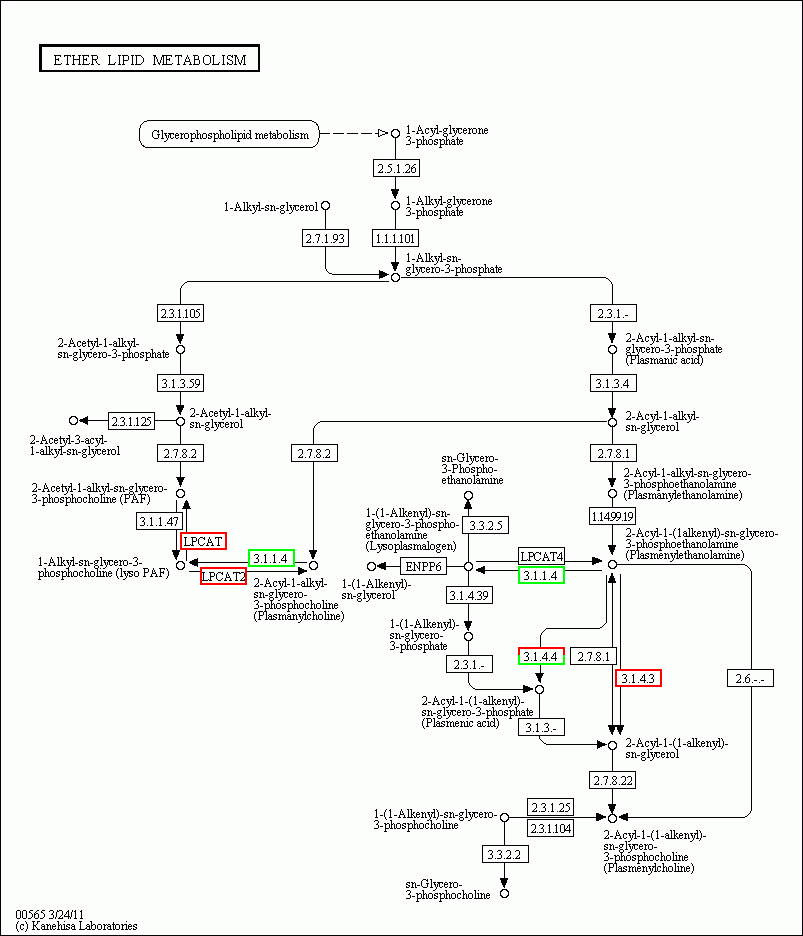

Supplement: Additional file 17: — KEGG analysis of differentially expressed genes between 72 h and 0 h (level3). [file 12864_2015_1373_MOESM17_ESM.zip › Additional file 5. KEGG analysis of differentially expressed genes between 72 h and 0 h (level3)/map00565.png]

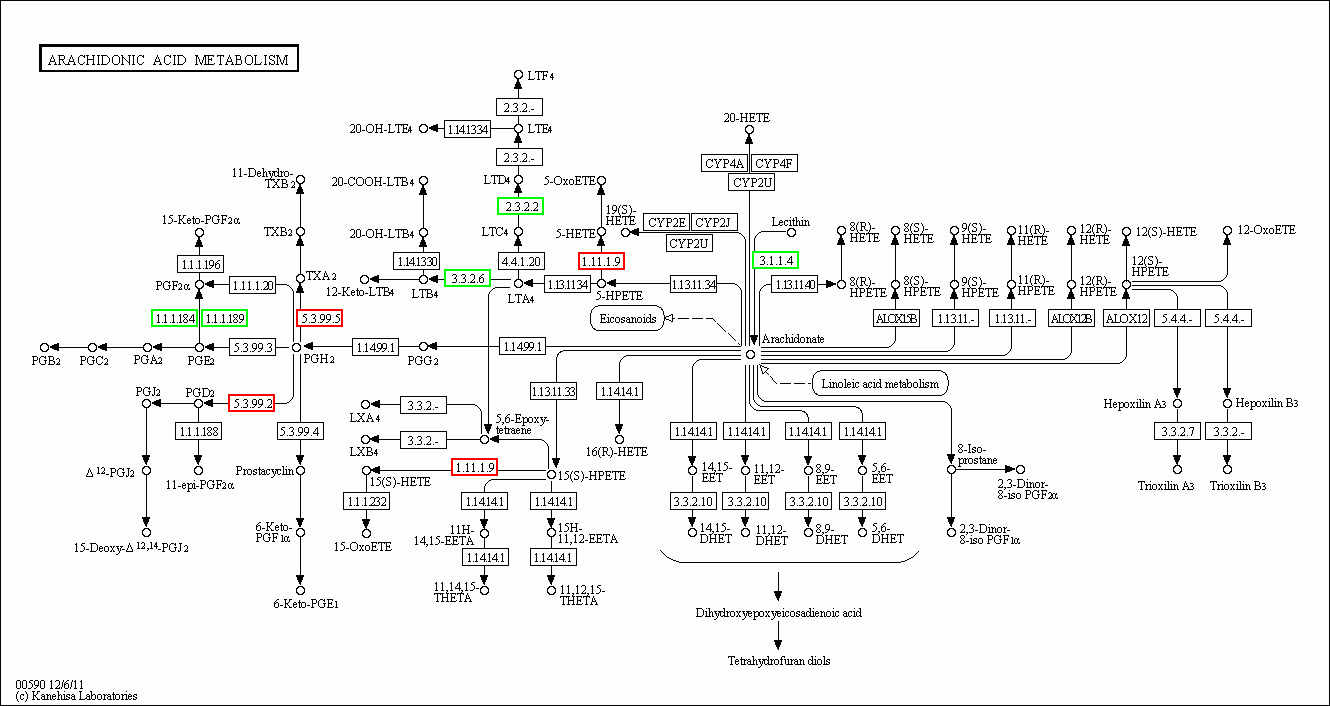

Supplement: Additional file 17: — KEGG analysis of differentially expressed genes between 72 h and 0 h (level3). [file 12864_2015_1373_MOESM17_ESM.zip › Additional file 5. KEGG analysis of differentially expressed genes between 72 h and 0 h (level3)/map00590.png]

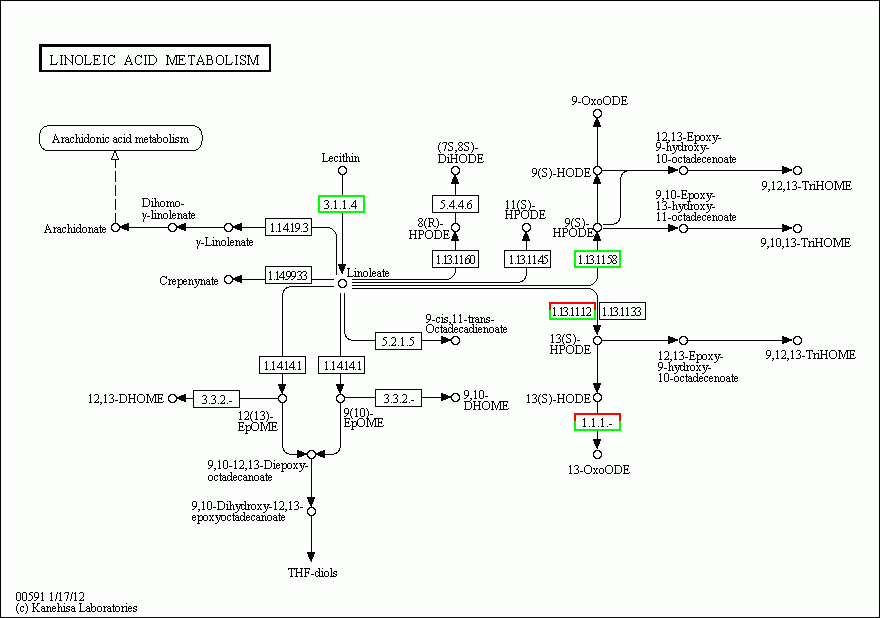

Supplement: Additional file 17: — KEGG analysis of differentially expressed genes between 72 h and 0 h (level3). [file 12864_2015_1373_MOESM17_ESM.zip › Additional file 5. KEGG analysis of differentially expressed genes between 72 h and 0 h (level3)/map00591.png]

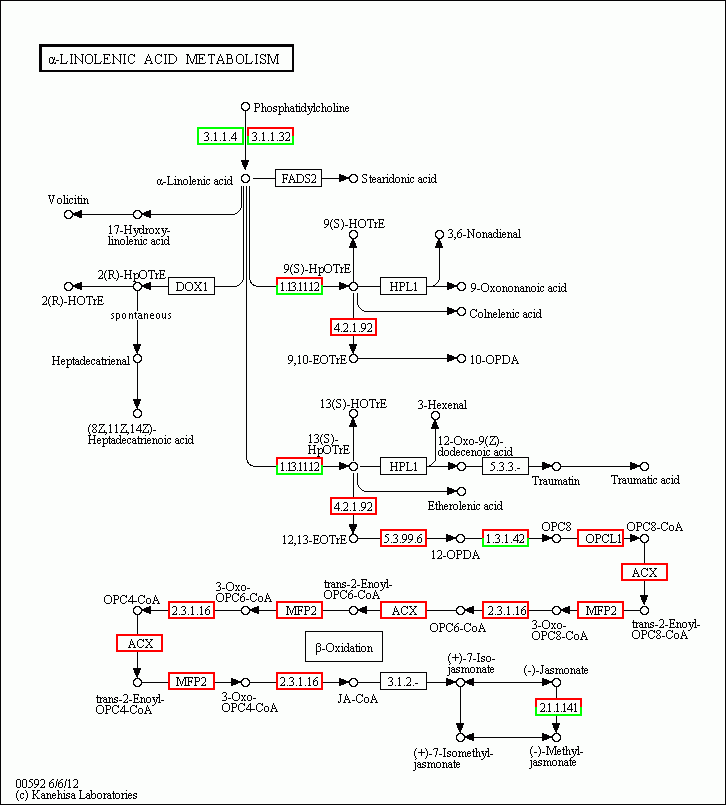

Supplement: Additional file 17: — KEGG analysis of differentially expressed genes between 72 h and 0 h (level3). [file 12864_2015_1373_MOESM17_ESM.zip › Additional file 5. KEGG analysis of differentially expressed genes between 72 h and 0 h (level3)/map00592.png]

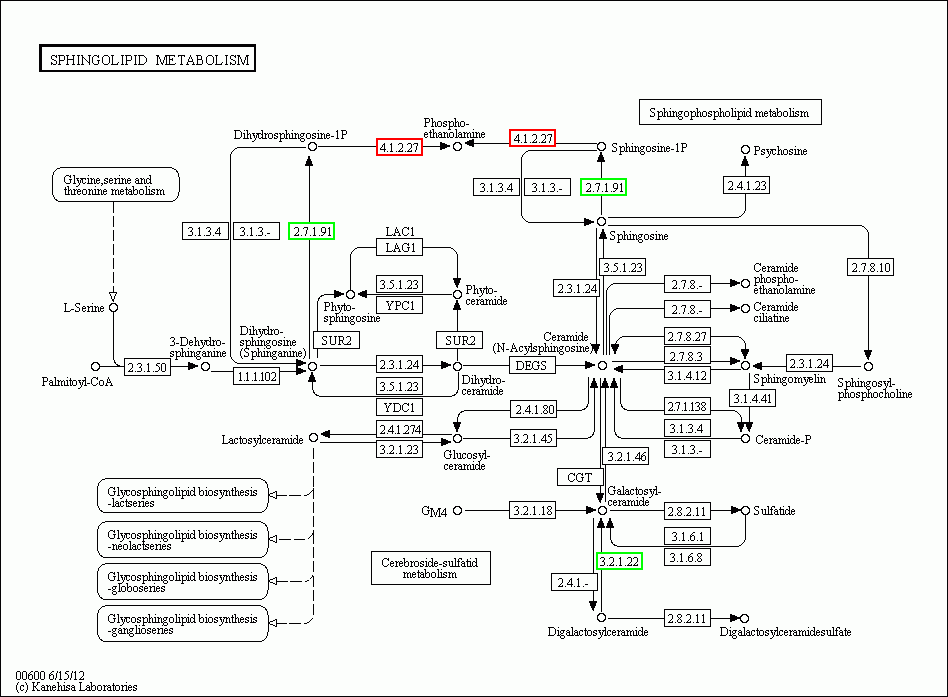

Supplement: Additional file 17: — KEGG analysis of differentially expressed genes between 72 h and 0 h (level3). [file 12864_2015_1373_MOESM17_ESM.zip › Additional file 5. KEGG analysis of differentially expressed genes between 72 h and 0 h (level3)/map00600.png]

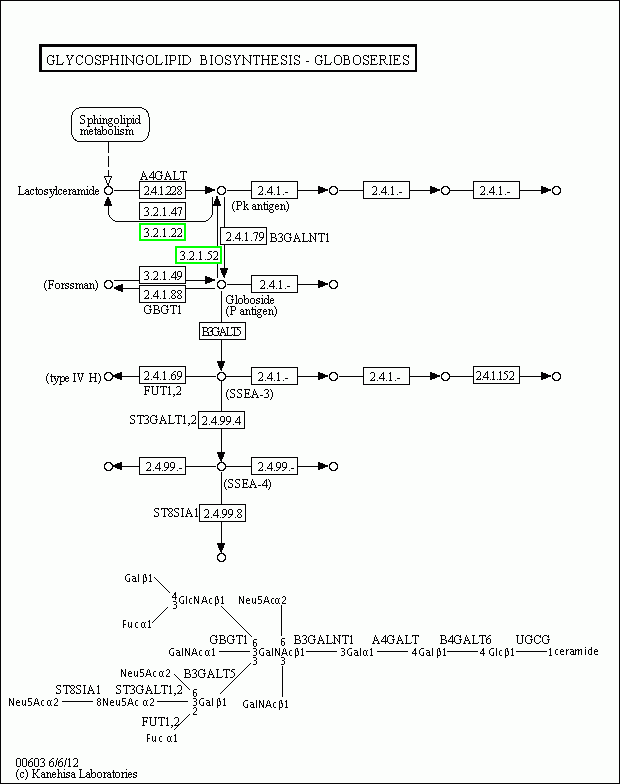

Supplement: Additional file 17: — KEGG analysis of differentially expressed genes between 72 h and 0 h (level3). [file 12864_2015_1373_MOESM17_ESM.zip › Additional file 5. KEGG analysis of differentially expressed genes between 72 h and 0 h (level3)/map00603.png]

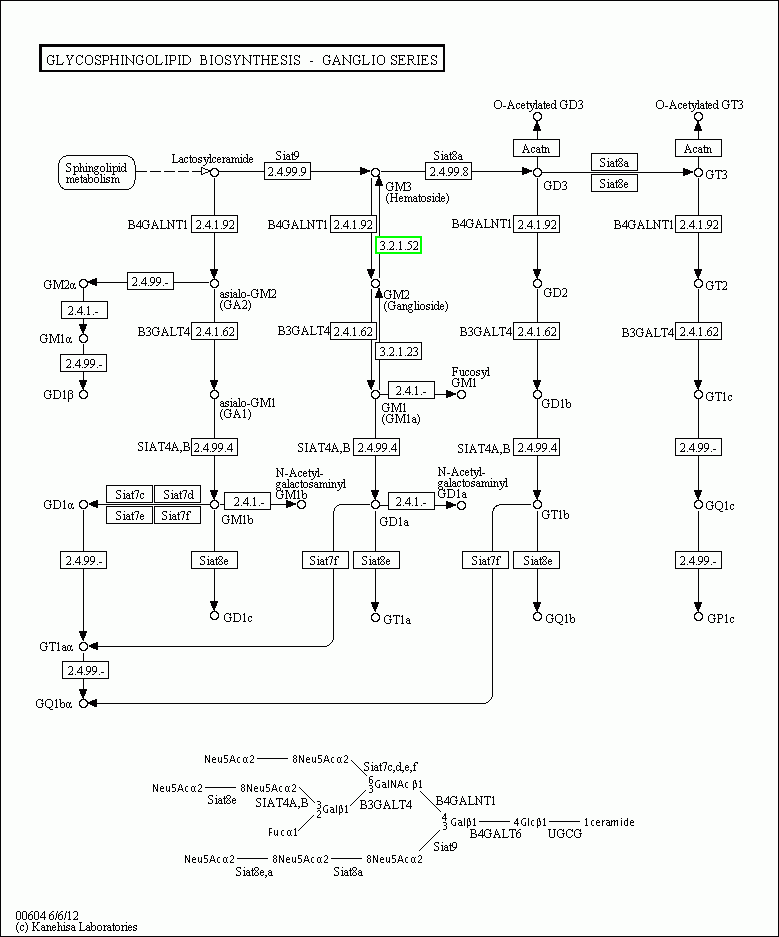

Supplement: Additional file 17: — KEGG analysis of differentially expressed genes between 72 h and 0 h (level3). [file 12864_2015_1373_MOESM17_ESM.zip › Additional file 5. KEGG analysis of differentially expressed genes between 72 h and 0 h (level3)/map00604.png]

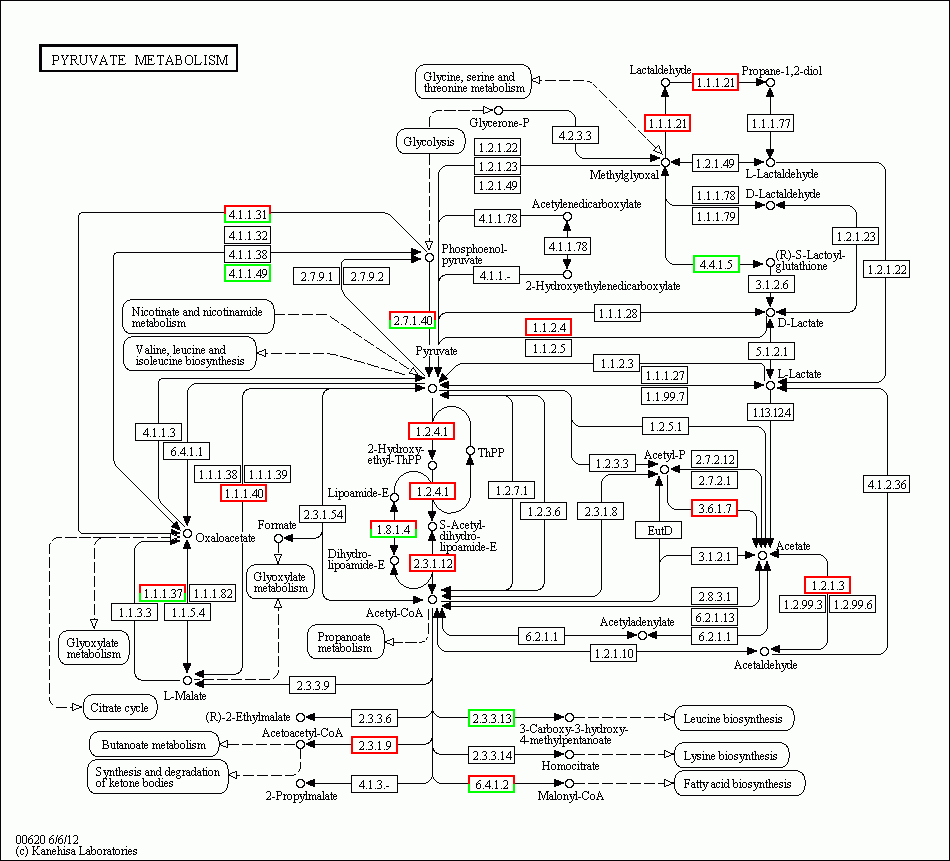

Supplement: Additional file 17: — KEGG analysis of differentially expressed genes between 72 h and 0 h (level3). [file 12864_2015_1373_MOESM17_ESM.zip › Additional file 5. KEGG analysis of differentially expressed genes between 72 h and 0 h (level3)/map00620.png]

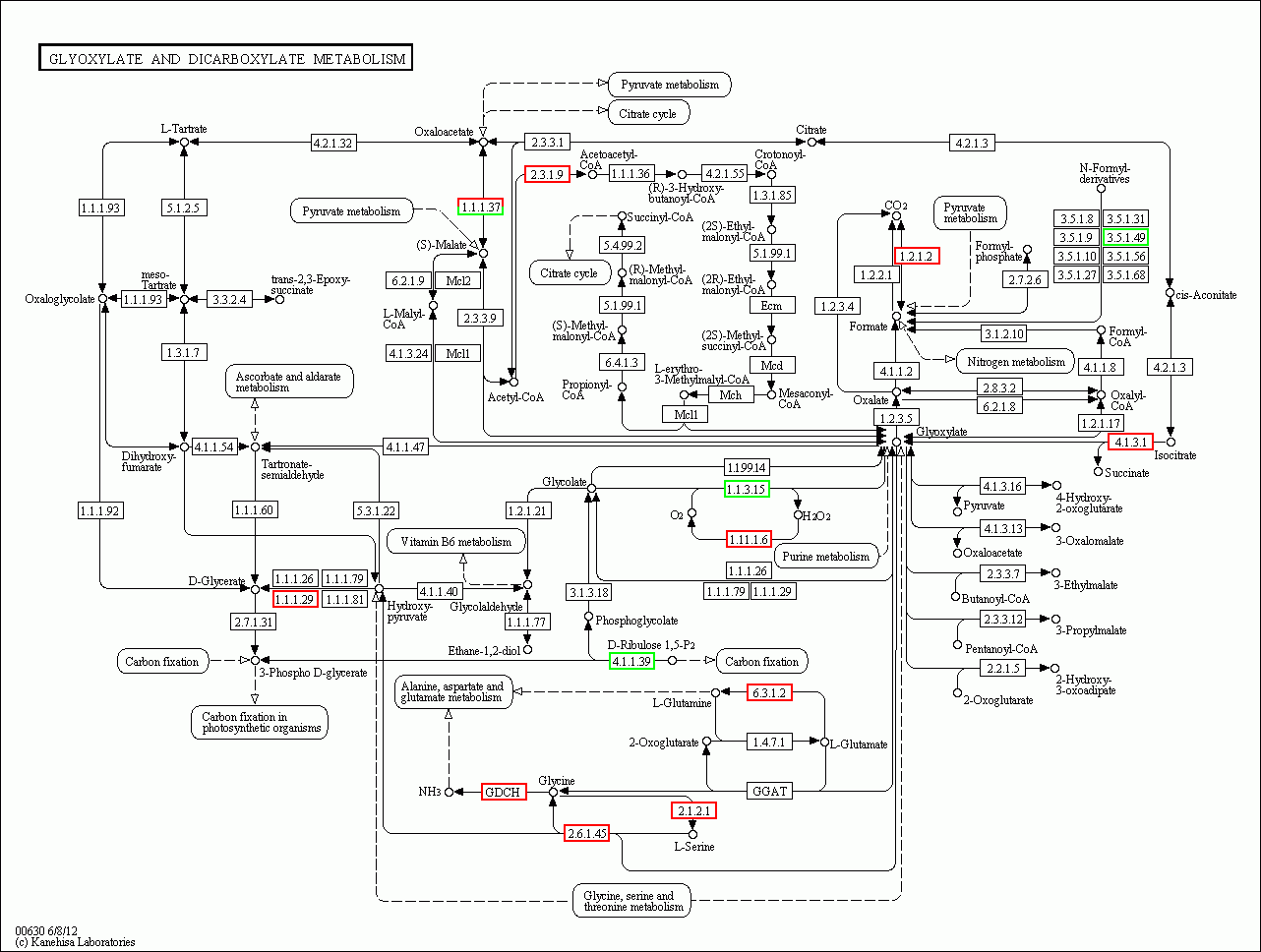

Supplement: Additional file 17: — KEGG analysis of differentially expressed genes between 72 h and 0 h (level3). [file 12864_2015_1373_MOESM17_ESM.zip › Additional file 5. KEGG analysis of differentially expressed genes between 72 h and 0 h (level3)/map00630.png]

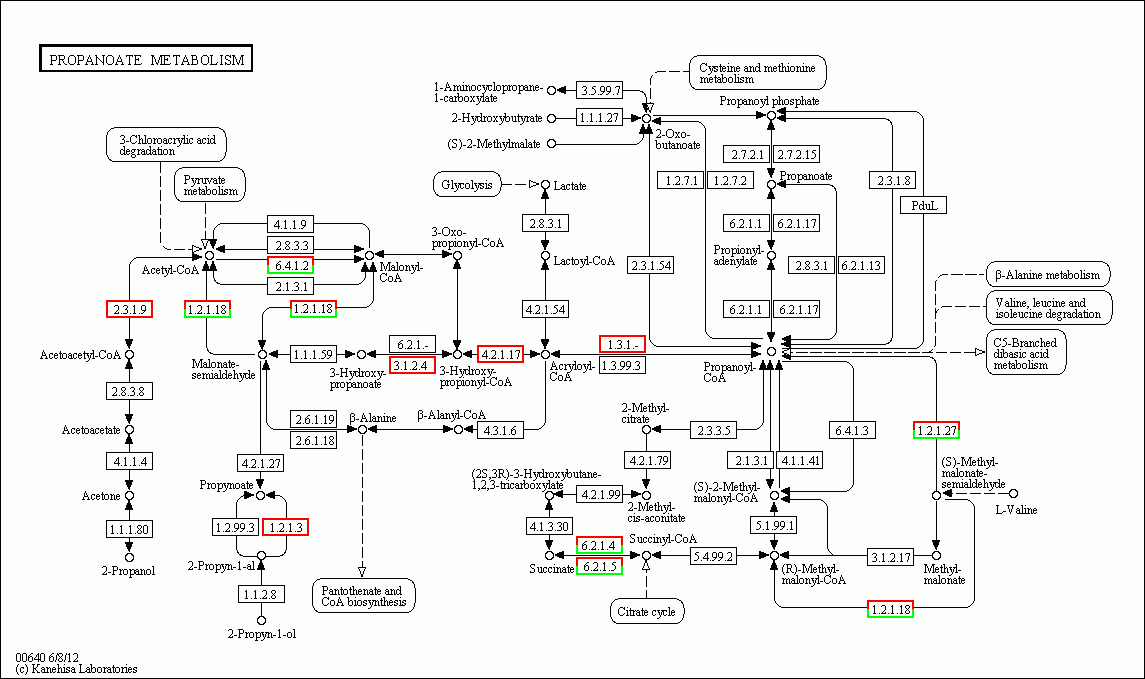

Supplement: Additional file 17: — KEGG analysis of differentially expressed genes between 72 h and 0 h (level3). [file 12864_2015_1373_MOESM17_ESM.zip › Additional file 5. KEGG analysis of differentially expressed genes between 72 h and 0 h (level3)/map00640.png]

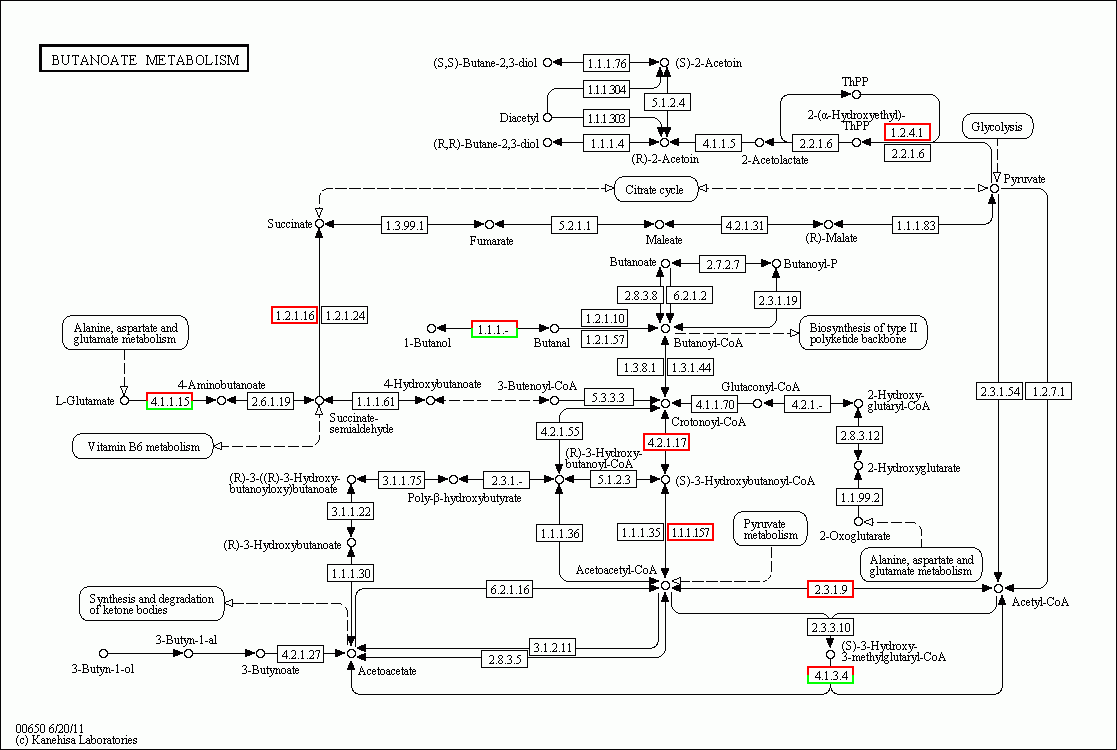

Supplement: Additional file 17: — KEGG analysis of differentially expressed genes between 72 h and 0 h (level3). [file 12864_2015_1373_MOESM17_ESM.zip › Additional file 5. KEGG analysis of differentially expressed genes between 72 h and 0 h (level3)/map00650.png]

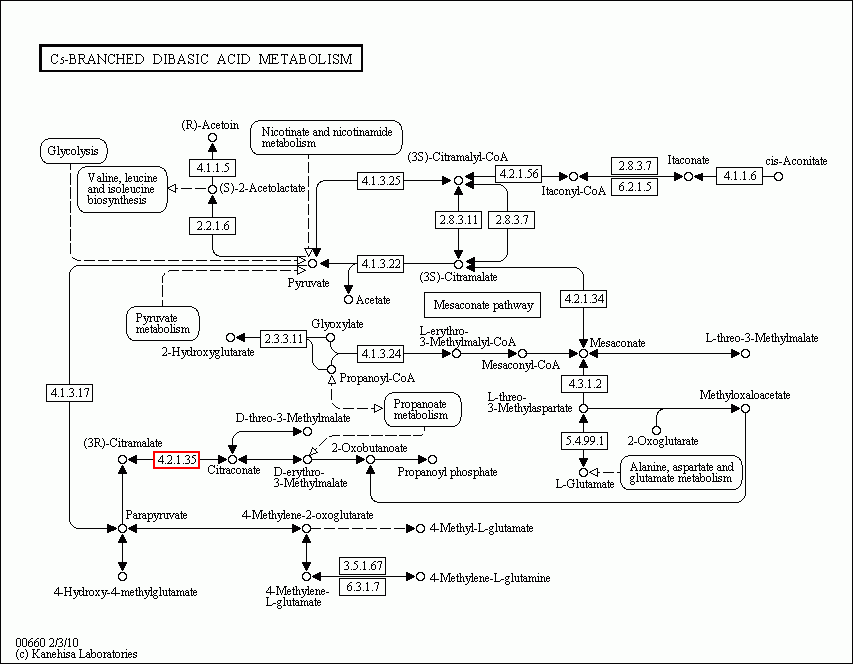

Supplement: Additional file 17: — KEGG analysis of differentially expressed genes between 72 h and 0 h (level3). [file 12864_2015_1373_MOESM17_ESM.zip › Additional file 5. KEGG analysis of differentially expressed genes between 72 h and 0 h (level3)/map00660.png]

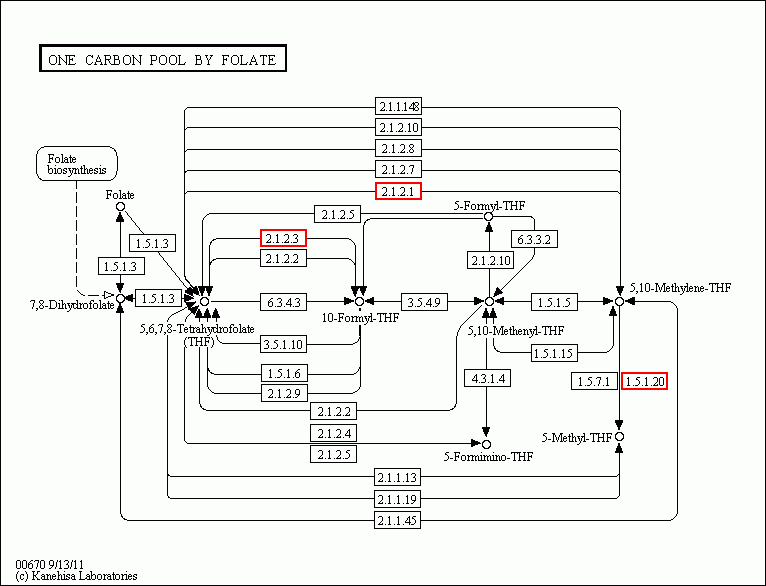

Supplement: Additional file 17: — KEGG analysis of differentially expressed genes between 72 h and 0 h (level3). [file 12864_2015_1373_MOESM17_ESM.zip › Additional file 5. KEGG analysis of differentially expressed genes between 72 h and 0 h (level3)/map00670.png]

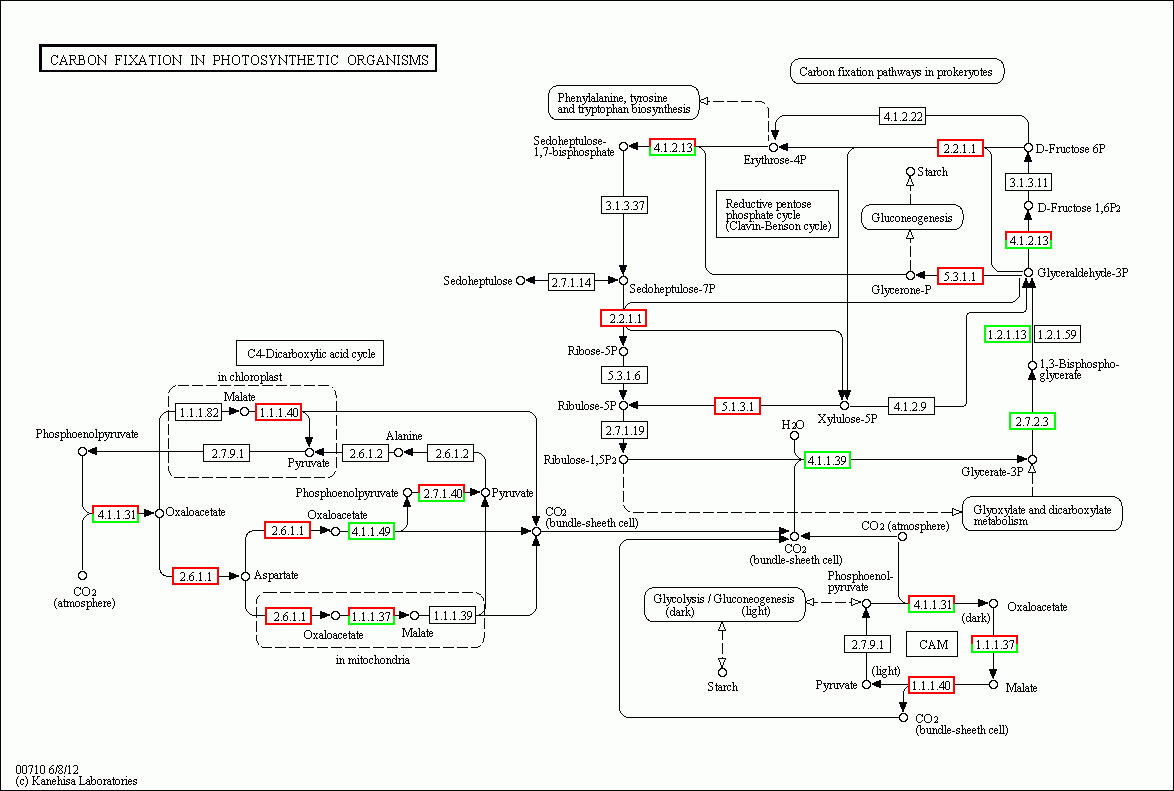

Supplement: Additional file 17: — KEGG analysis of differentially expressed genes between 72 h and 0 h (level3). [file 12864_2015_1373_MOESM17_ESM.zip › Additional file 5. KEGG analysis of differentially expressed genes between 72 h and 0 h (level3)/map00710.png]

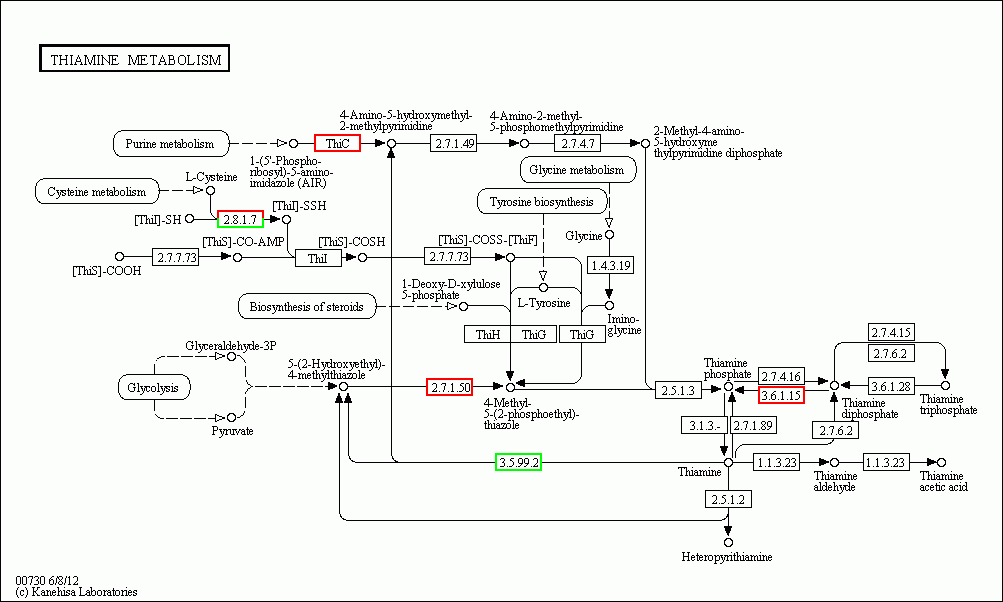

Supplement: Additional file 17: — KEGG analysis of differentially expressed genes between 72 h and 0 h (level3). [file 12864_2015_1373_MOESM17_ESM.zip › Additional file 5. KEGG analysis of differentially expressed genes between 72 h and 0 h (level3)/map00730.png]

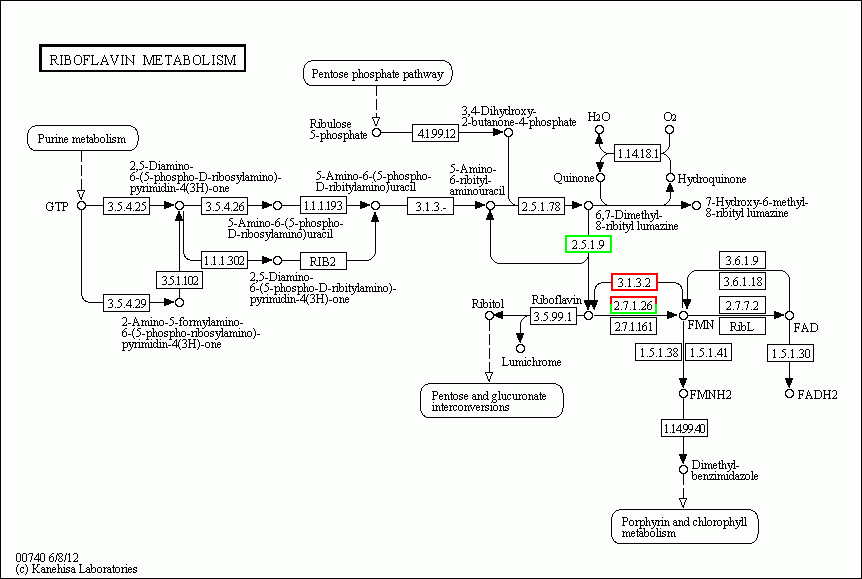

Supplement: Additional file 17: — KEGG analysis of differentially expressed genes between 72 h and 0 h (level3). [file 12864_2015_1373_MOESM17_ESM.zip › Additional file 5. KEGG analysis of differentially expressed genes between 72 h and 0 h (level3)/map00740.png]

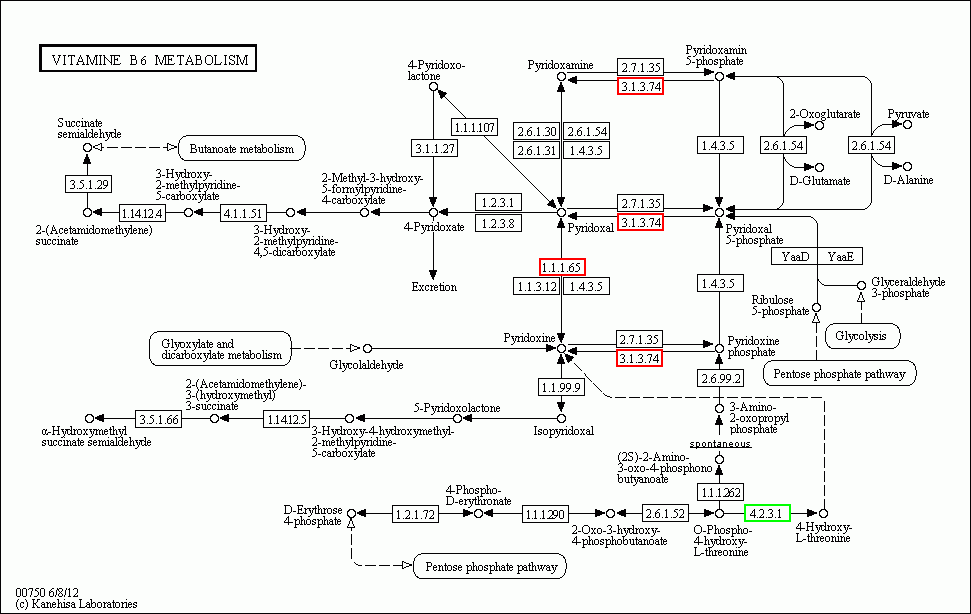

Supplement: Additional file 17: — KEGG analysis of differentially expressed genes between 72 h and 0 h (level3). [file 12864_2015_1373_MOESM17_ESM.zip › Additional file 5. KEGG analysis of differentially expressed genes between 72 h and 0 h (level3)/map00750.png]

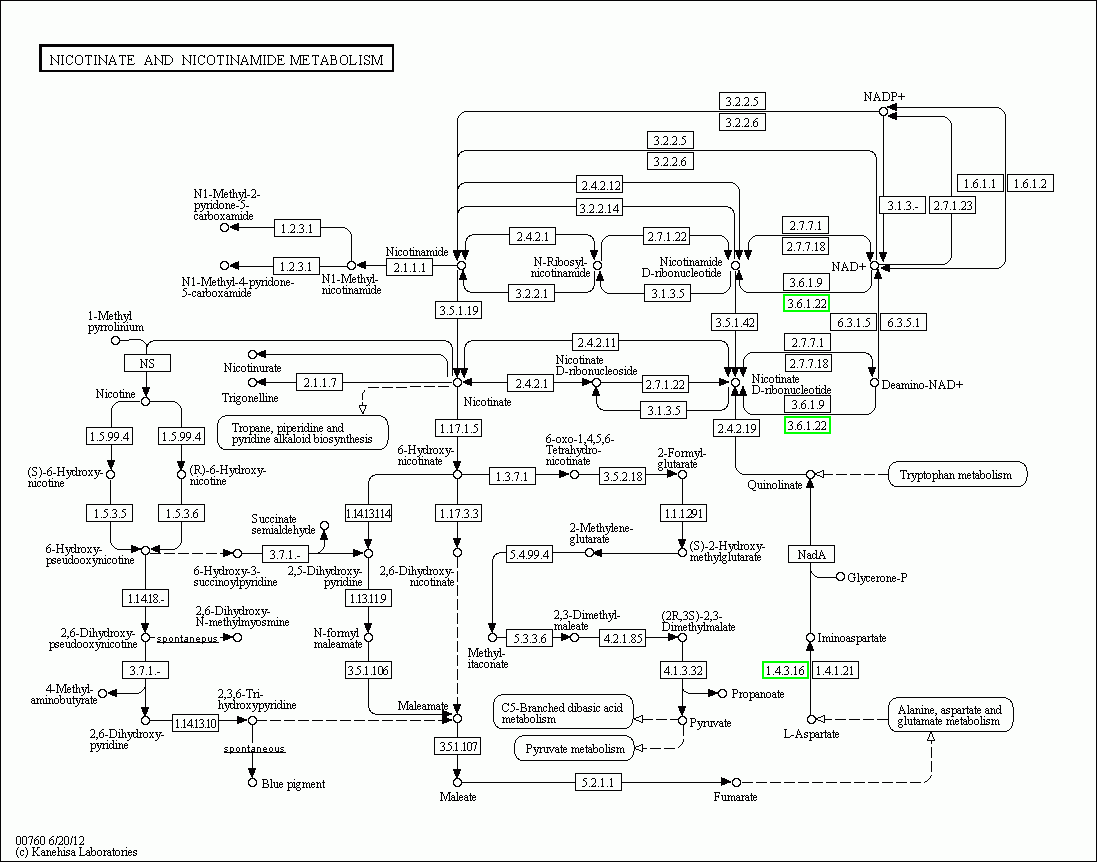

Supplement: Additional file 17: — KEGG analysis of differentially expressed genes between 72 h and 0 h (level3). [file 12864_2015_1373_MOESM17_ESM.zip › Additional file 5. KEGG analysis of differentially expressed genes between 72 h and 0 h (level3)/map00760.png]

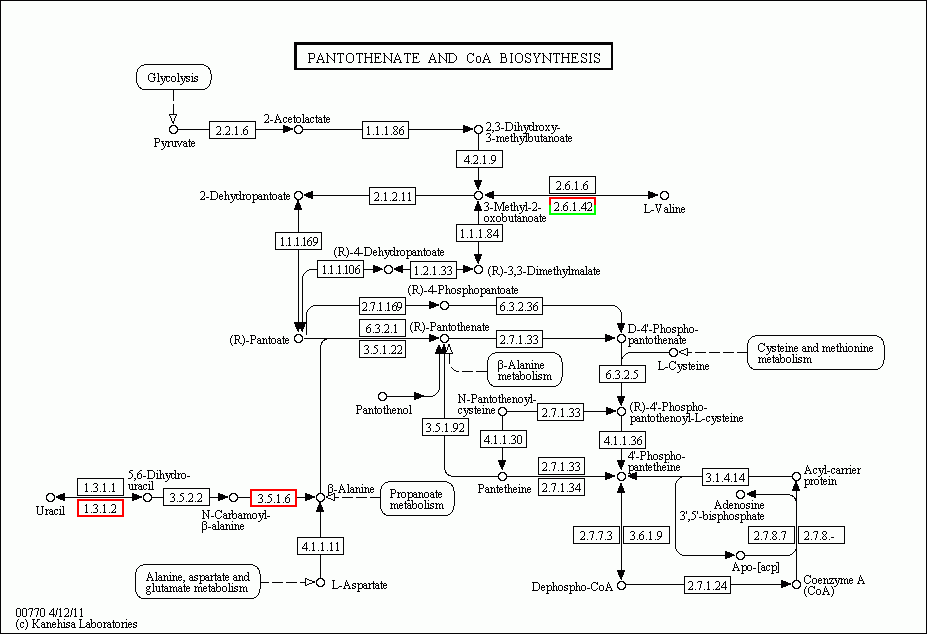

Supplement: Additional file 17: — KEGG analysis of differentially expressed genes between 72 h and 0 h (level3). [file 12864_2015_1373_MOESM17_ESM.zip › Additional file 5. KEGG analysis of differentially expressed genes between 72 h and 0 h (level3)/map00770.png]

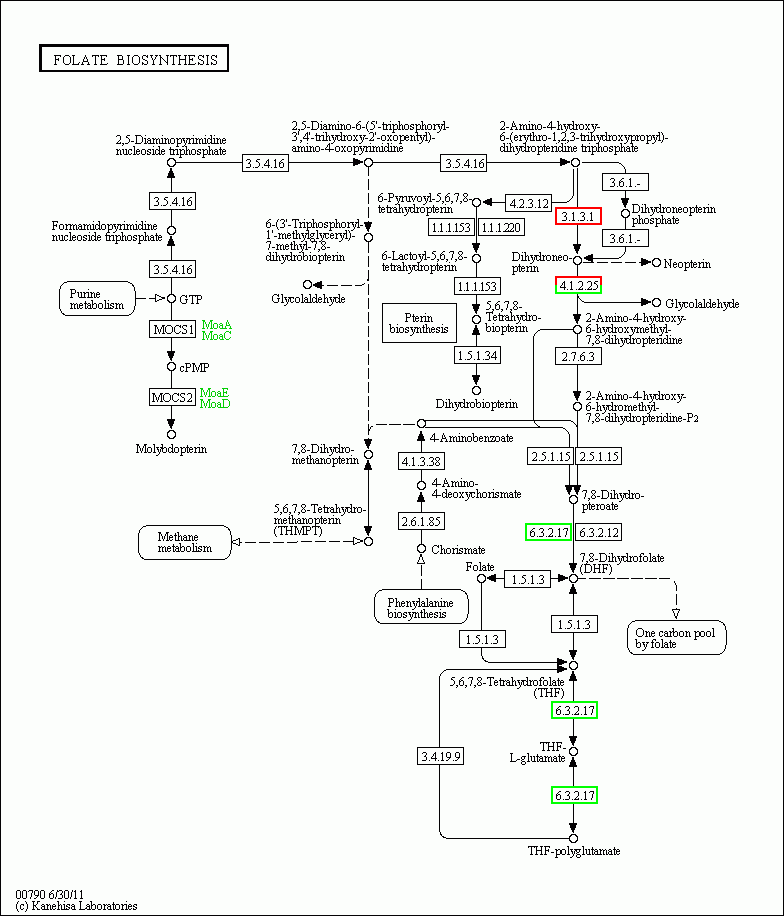

Supplement: Additional file 17: — KEGG analysis of differentially expressed genes between 72 h and 0 h (level3). [file 12864_2015_1373_MOESM17_ESM.zip › Additional file 5. KEGG analysis of differentially expressed genes between 72 h and 0 h (level3)/map00790.png]

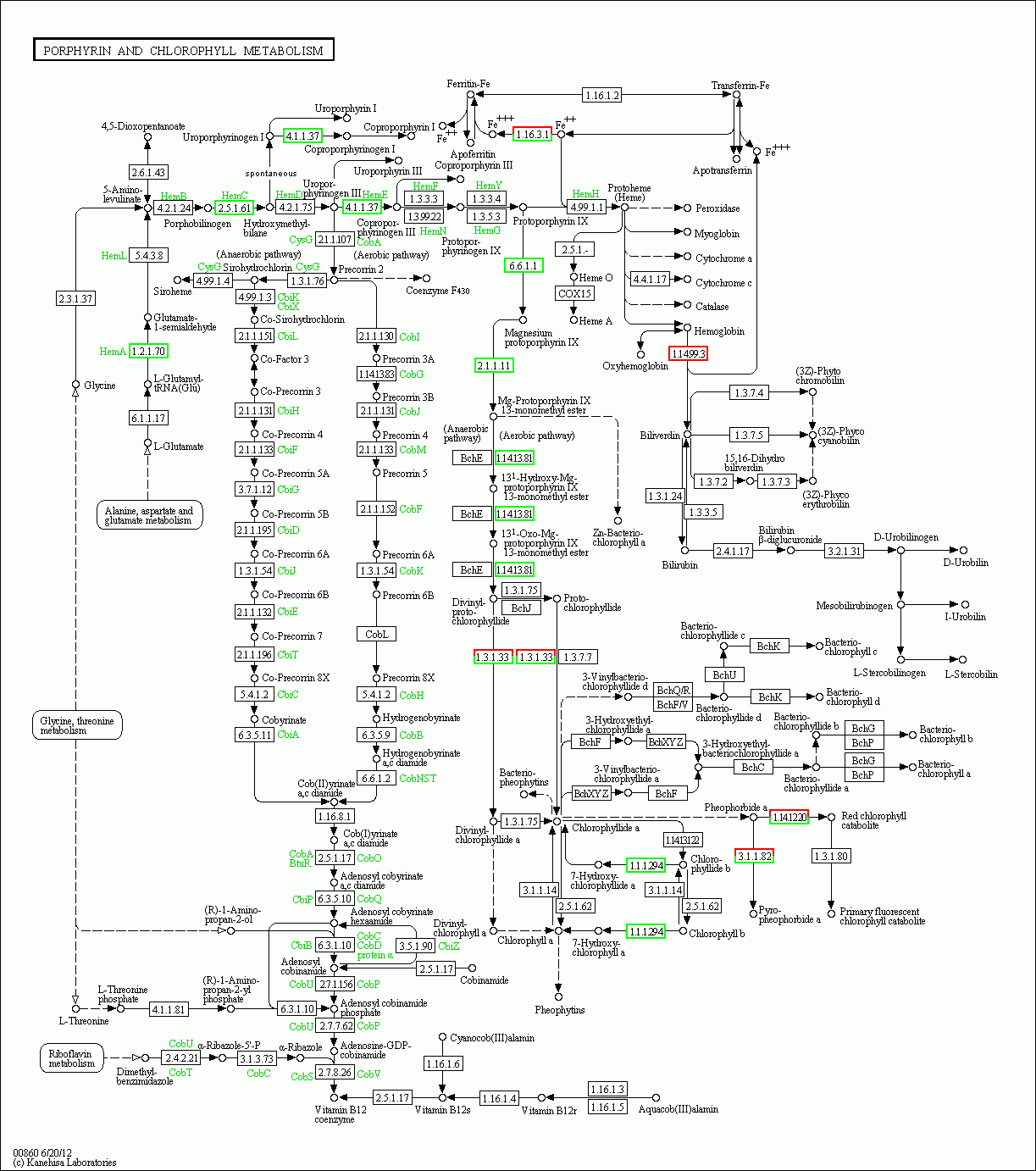

Supplement: Additional file 17: — KEGG analysis of differentially expressed genes between 72 h and 0 h (level3). [file 12864_2015_1373_MOESM17_ESM.zip › Additional file 5. KEGG analysis of differentially expressed genes between 72 h and 0 h (level3)/map00860.png]

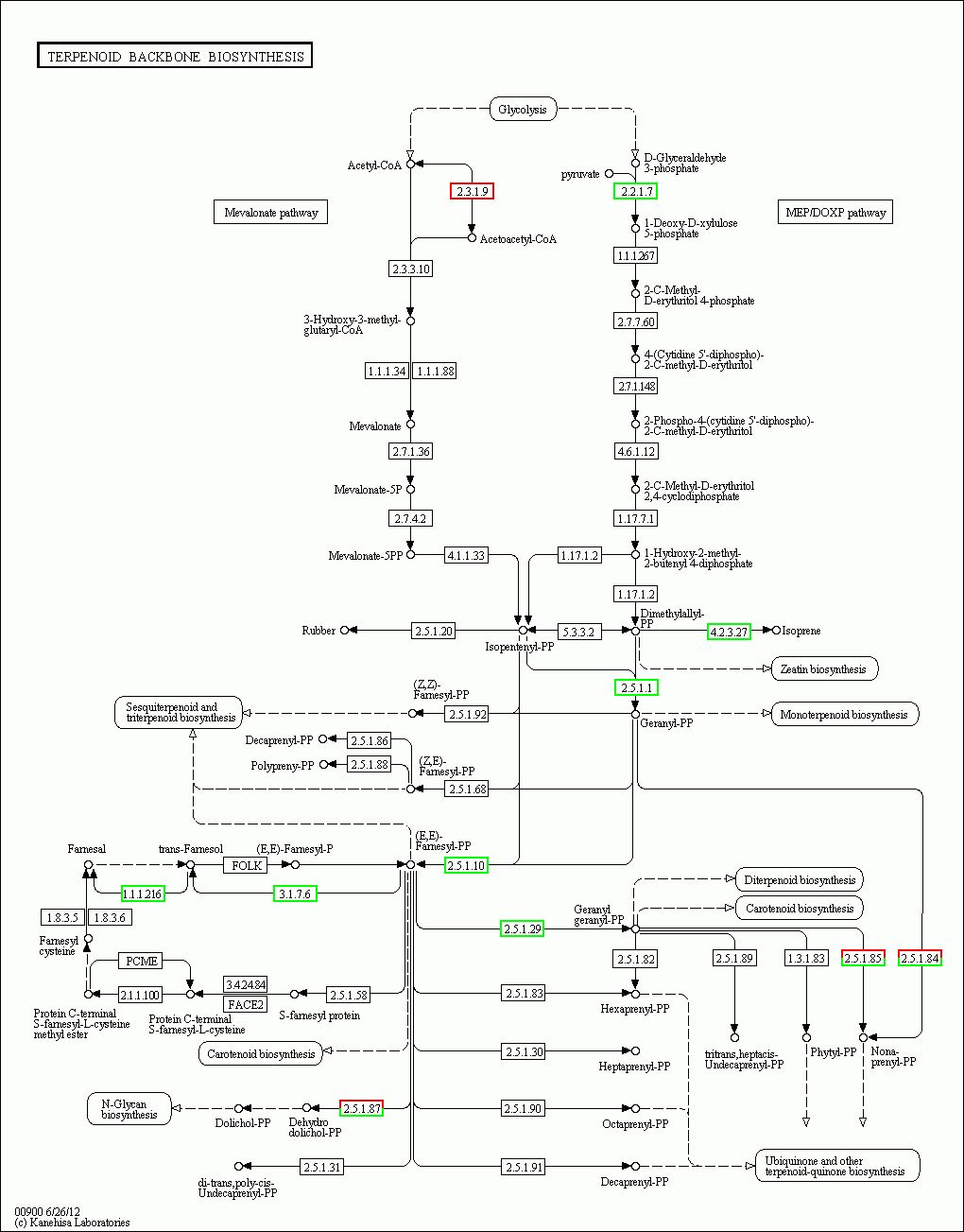

Supplement: Additional file 17: — KEGG analysis of differentially expressed genes between 72 h and 0 h (level3). [file 12864_2015_1373_MOESM17_ESM.zip › Additional file 5. KEGG analysis of differentially expressed genes between 72 h and 0 h (level3)/map00900.png]

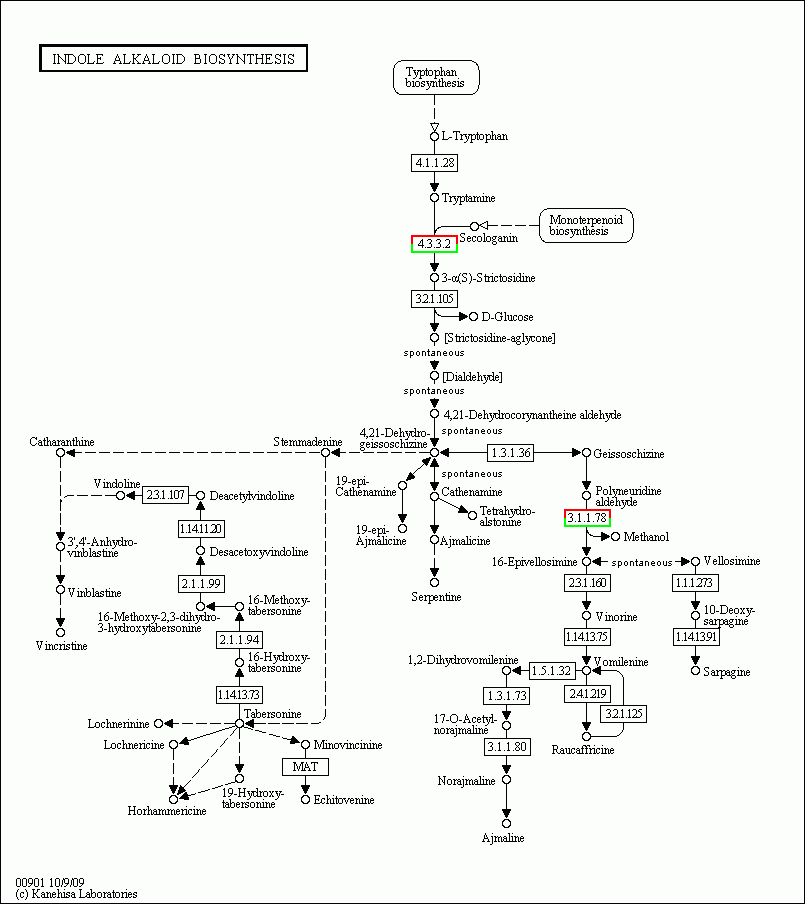

Supplement: Additional file 17: — KEGG analysis of differentially expressed genes between 72 h and 0 h (level3). [file 12864_2015_1373_MOESM17_ESM.zip › Additional file 5. KEGG analysis of differentially expressed genes between 72 h and 0 h (level3)/map00901.png]

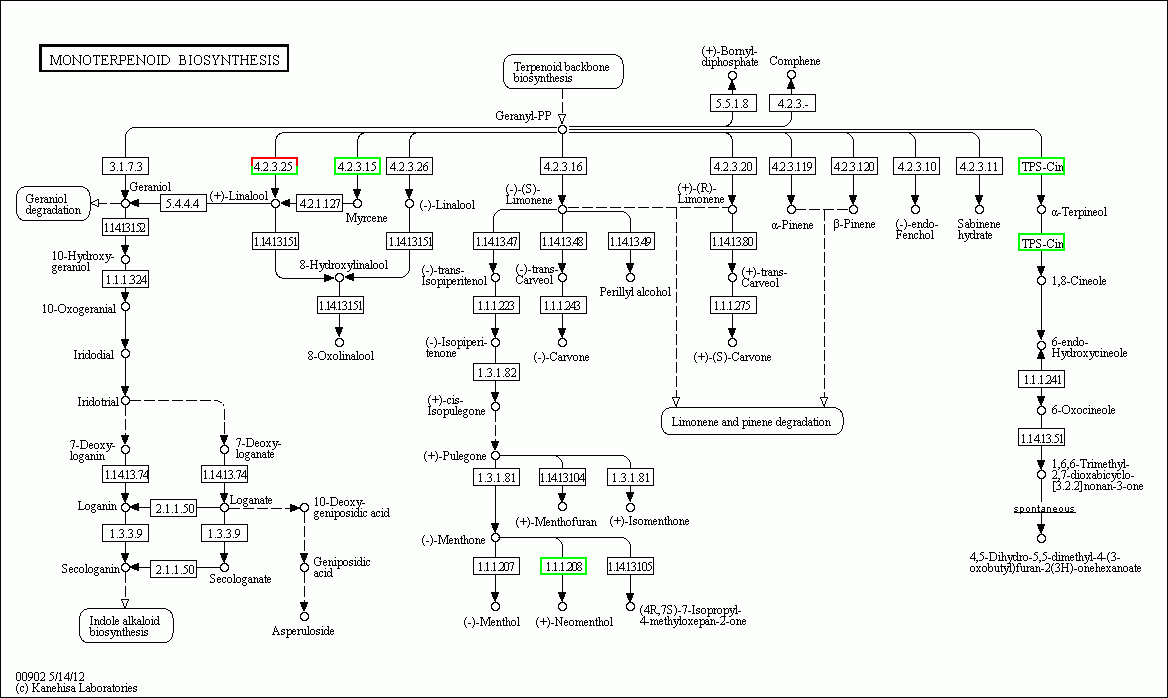

Supplement: Additional file 17: — KEGG analysis of differentially expressed genes between 72 h and 0 h (level3). [file 12864_2015_1373_MOESM17_ESM.zip › Additional file 5. KEGG analysis of differentially expressed genes between 72 h and 0 h (level3)/map00902.png]

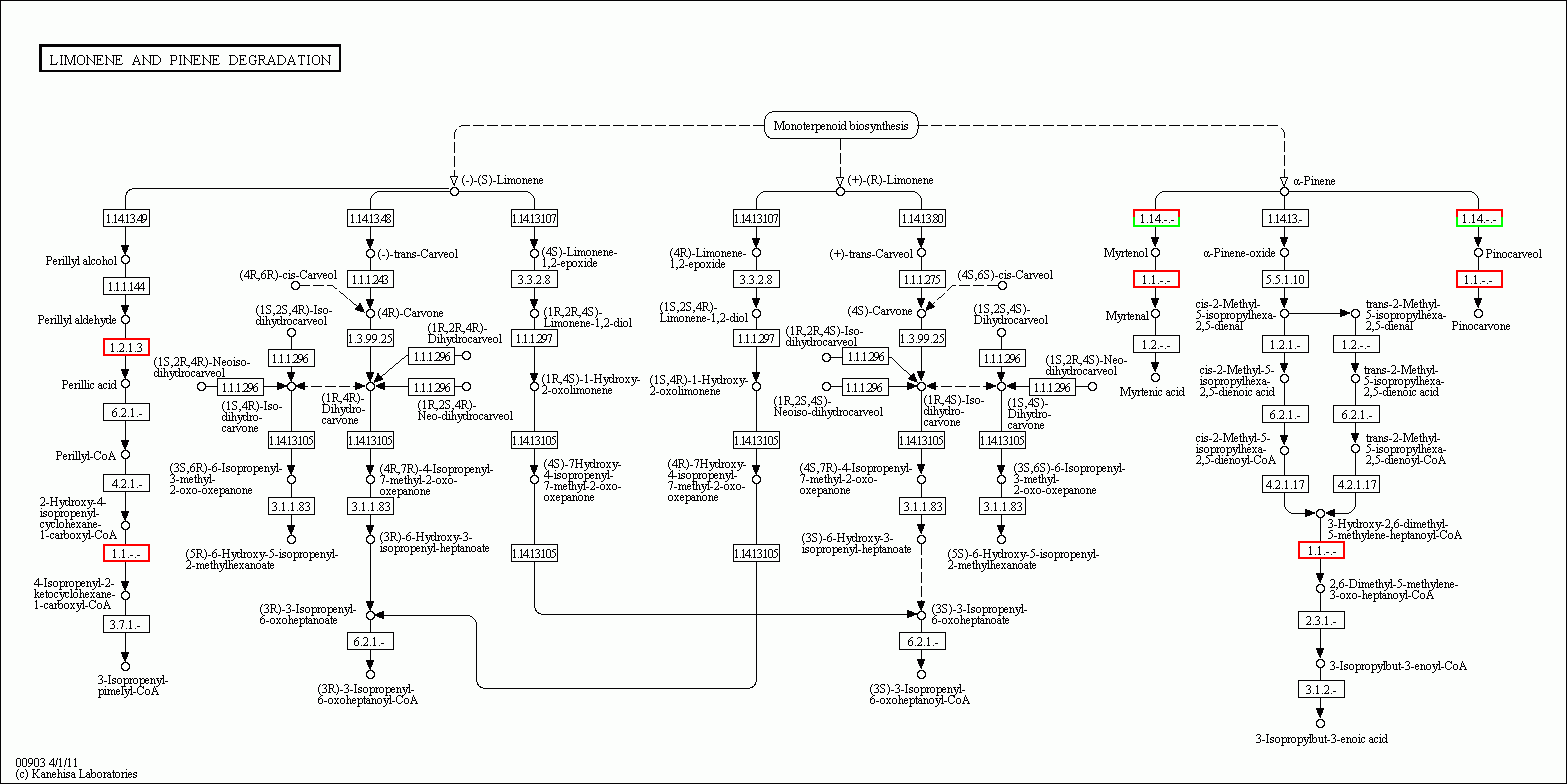

Supplement: Additional file 17: — KEGG analysis of differentially expressed genes between 72 h and 0 h (level3). [file 12864_2015_1373_MOESM17_ESM.zip › Additional file 5. KEGG analysis of differentially expressed genes between 72 h and 0 h (level3)/map00903.png]

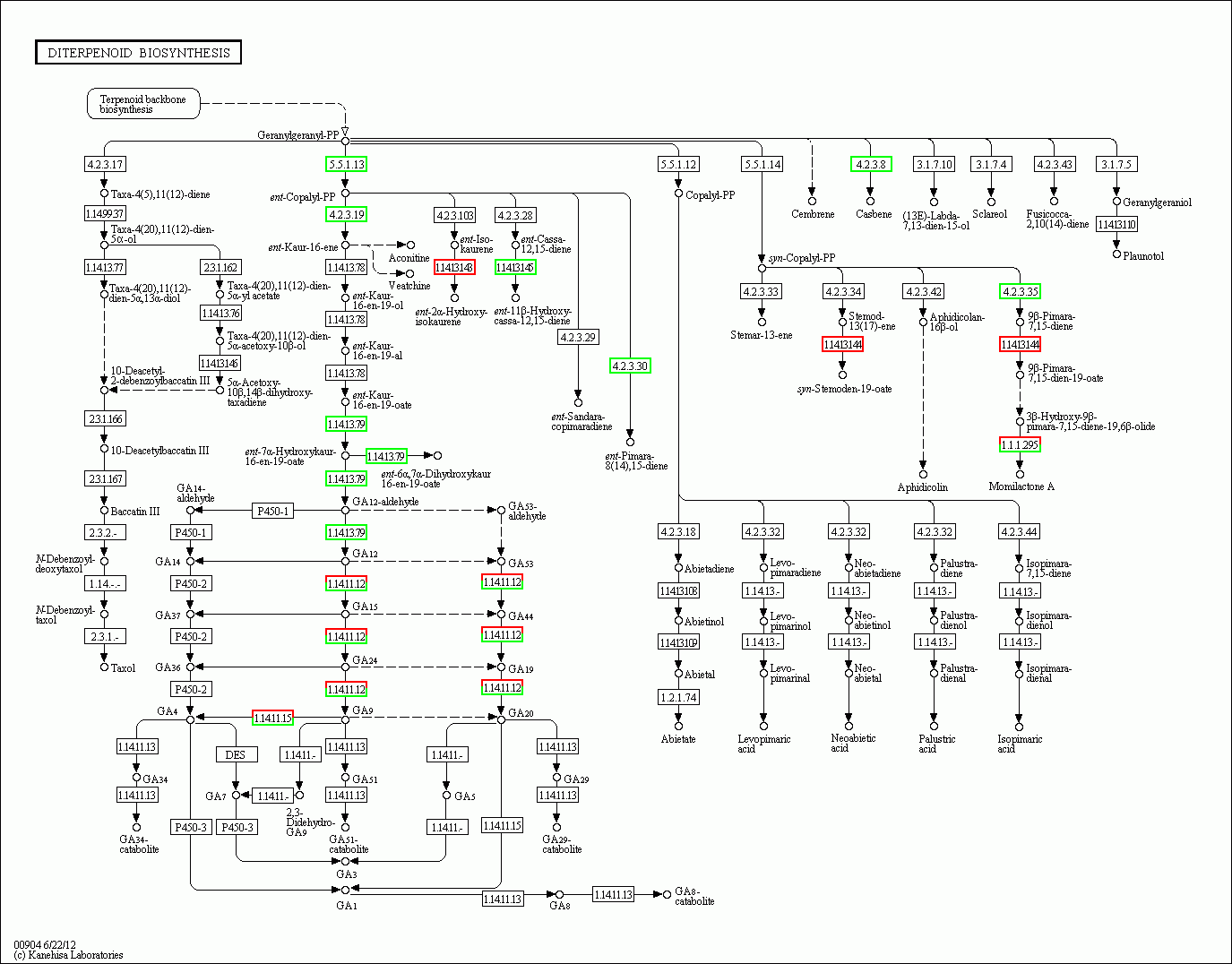

Supplement: Additional file 17: — KEGG analysis of differentially expressed genes between 72 h and 0 h (level3). [file 12864_2015_1373_MOESM17_ESM.zip › Additional file 5. KEGG analysis of differentially expressed genes between 72 h and 0 h (level3)/map00904.png]

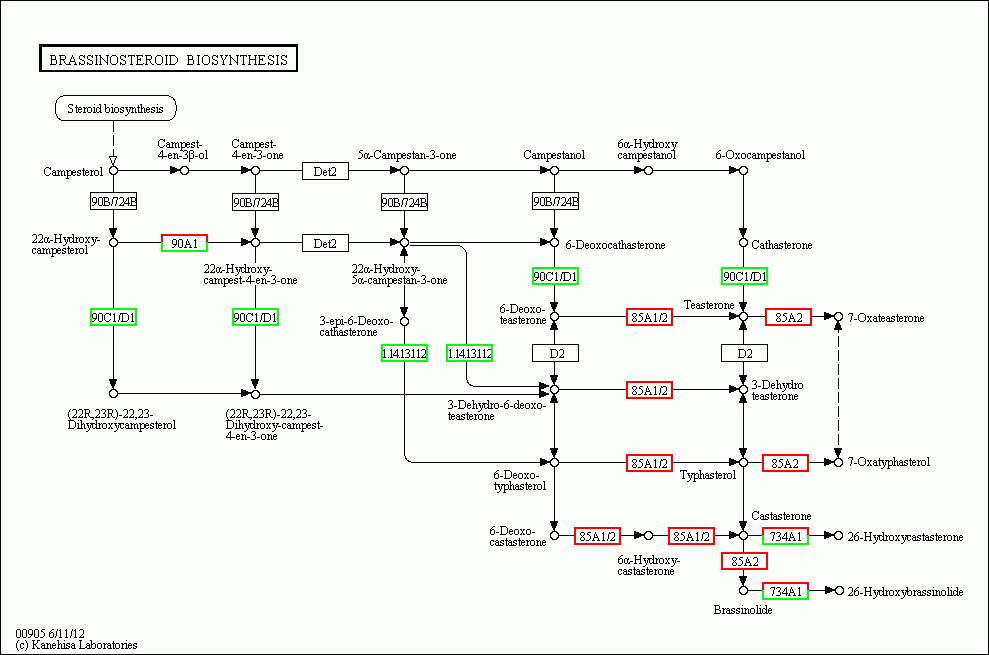

Supplement: Additional file 17: — KEGG analysis of differentially expressed genes between 72 h and 0 h (level3). [file 12864_2015_1373_MOESM17_ESM.zip › Additional file 5. KEGG analysis of differentially expressed genes between 72 h and 0 h (level3)/map00905.png]

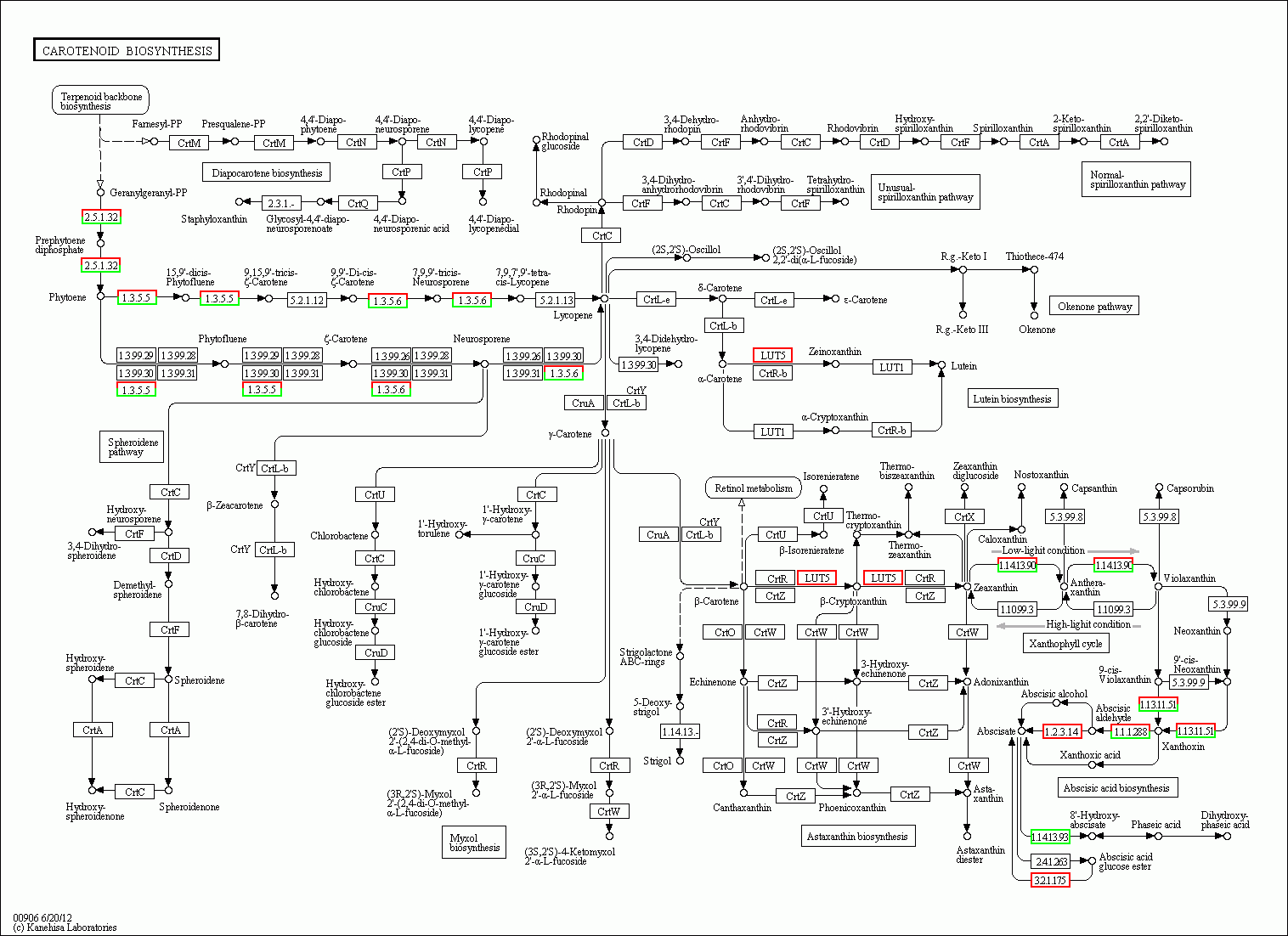

Supplement: Additional file 17: — KEGG analysis of differentially expressed genes between 72 h and 0 h (level3). [file 12864_2015_1373_MOESM17_ESM.zip › Additional file 5. KEGG analysis of differentially expressed genes between 72 h and 0 h (level3)/map00906.png]

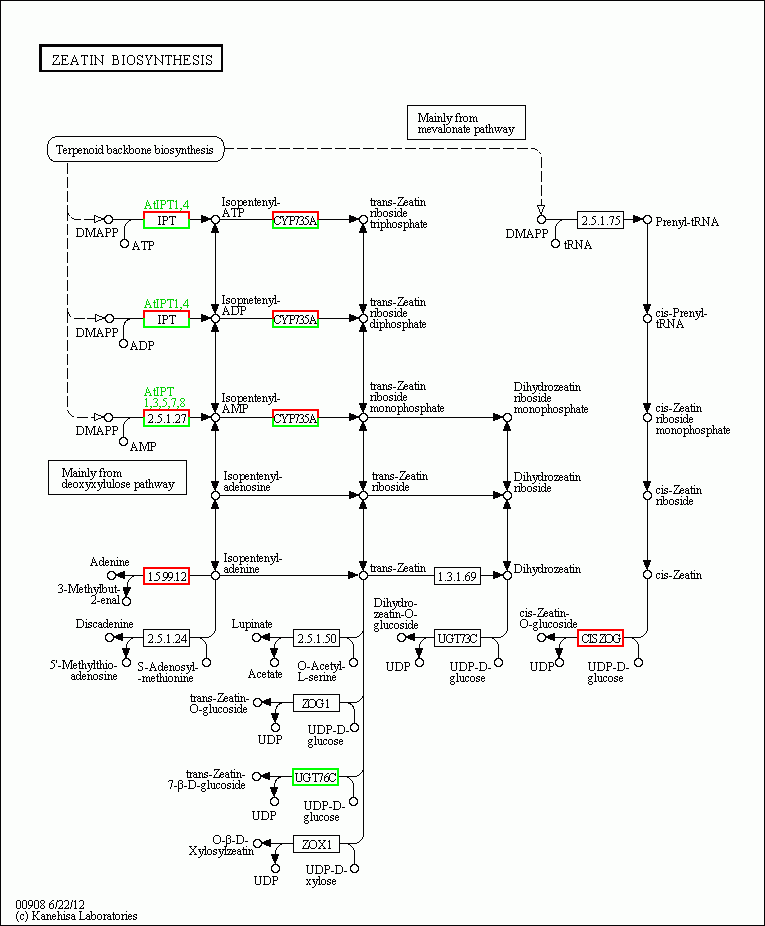

Supplement: Additional file 17: — KEGG analysis of differentially expressed genes between 72 h and 0 h (level3). [file 12864_2015_1373_MOESM17_ESM.zip › Additional file 5. KEGG analysis of differentially expressed genes between 72 h and 0 h (level3)/map00908.png]

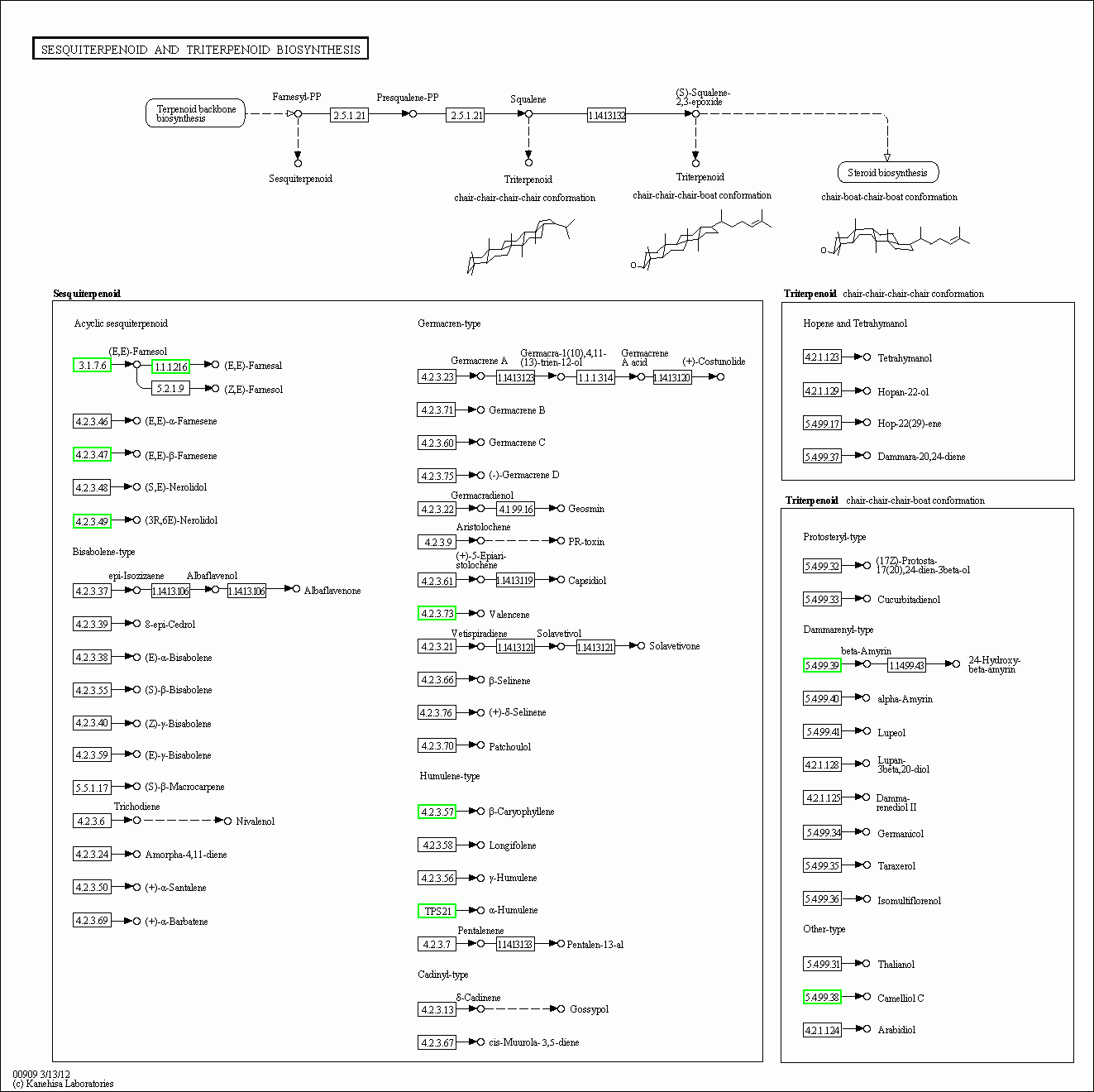

Supplement: Additional file 17: — KEGG analysis of differentially expressed genes between 72 h and 0 h (level3). [file 12864_2015_1373_MOESM17_ESM.zip › Additional file 5. KEGG analysis of differentially expressed genes between 72 h and 0 h (level3)/map00909.png]

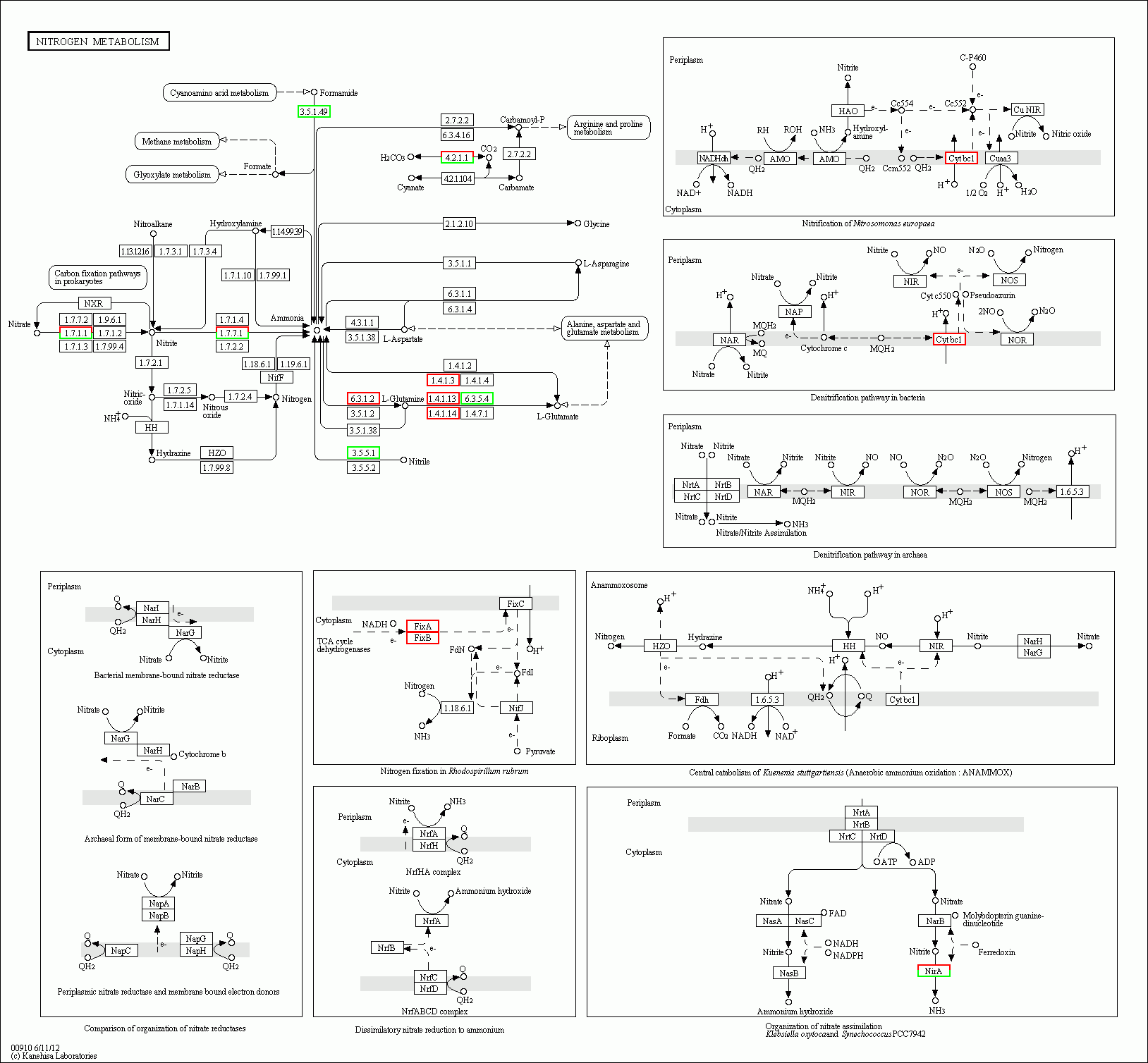

Supplement: Additional file 17: — KEGG analysis of differentially expressed genes between 72 h and 0 h (level3). [file 12864_2015_1373_MOESM17_ESM.zip › Additional file 5. KEGG analysis of differentially expressed genes between 72 h and 0 h (level3)/map00910.png]

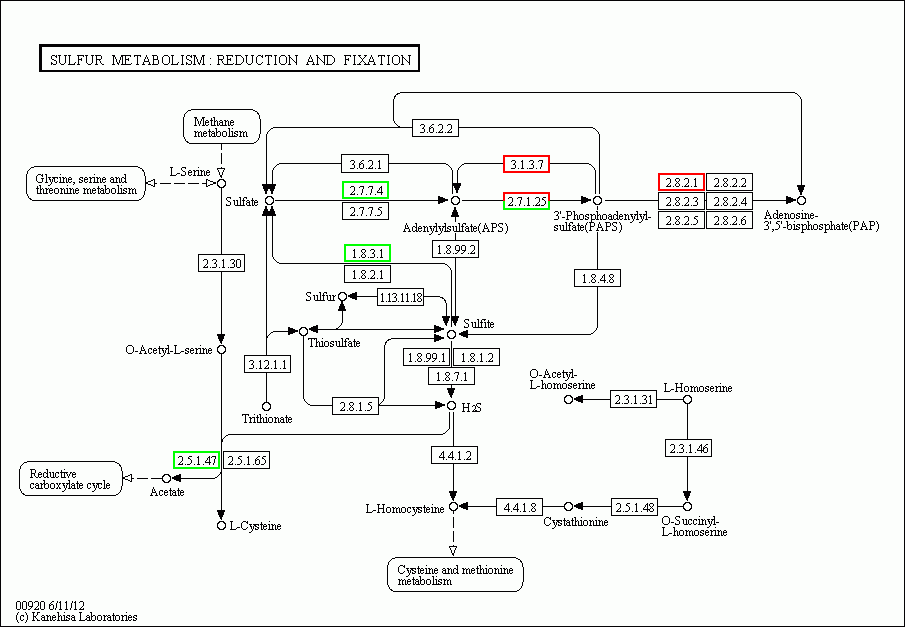

Supplement: Additional file 17: — KEGG analysis of differentially expressed genes between 72 h and 0 h (level3). [file 12864_2015_1373_MOESM17_ESM.zip › Additional file 5. KEGG analysis of differentially expressed genes between 72 h and 0 h (level3)/map00920.png]

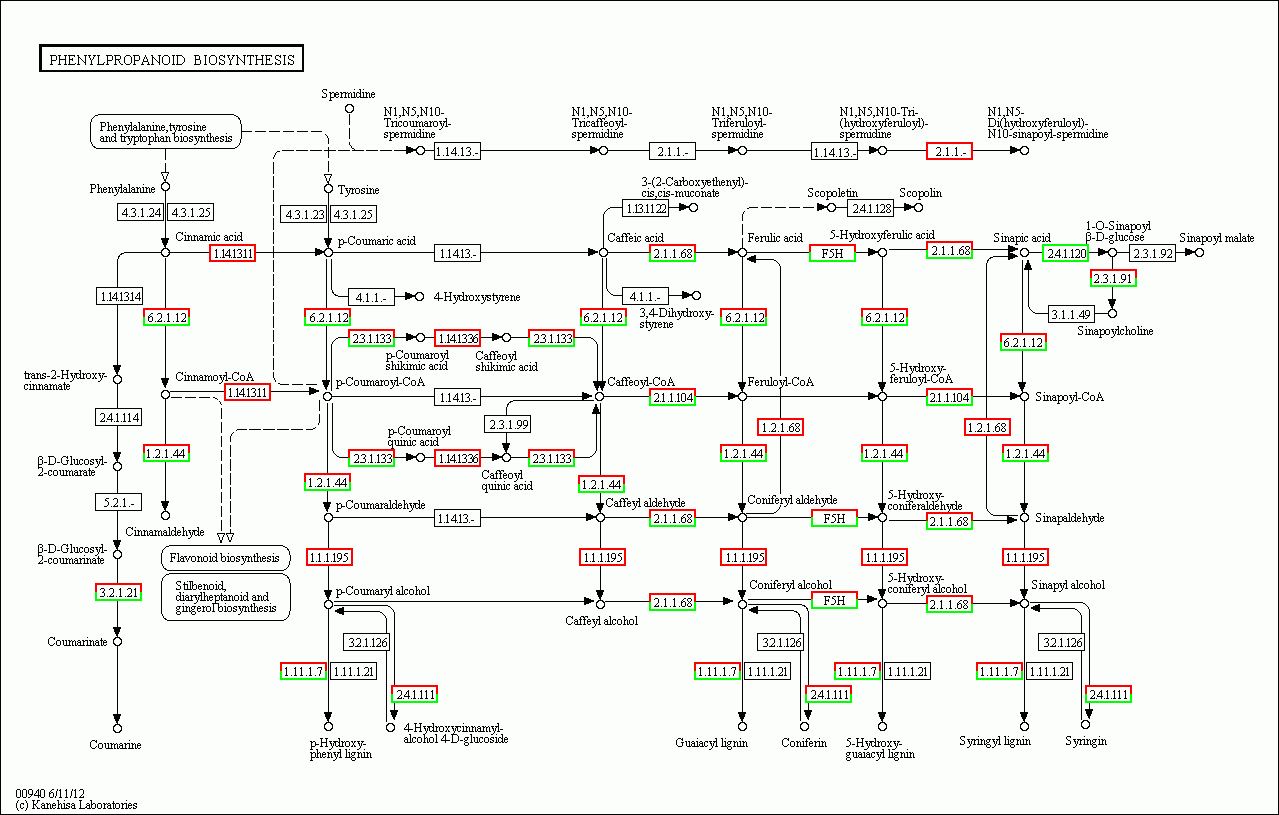

Supplement: Additional file 17: — KEGG analysis of differentially expressed genes between 72 h and 0 h (level3). [file 12864_2015_1373_MOESM17_ESM.zip › Additional file 5. KEGG analysis of differentially expressed genes between 72 h and 0 h (level3)/map00940.png]

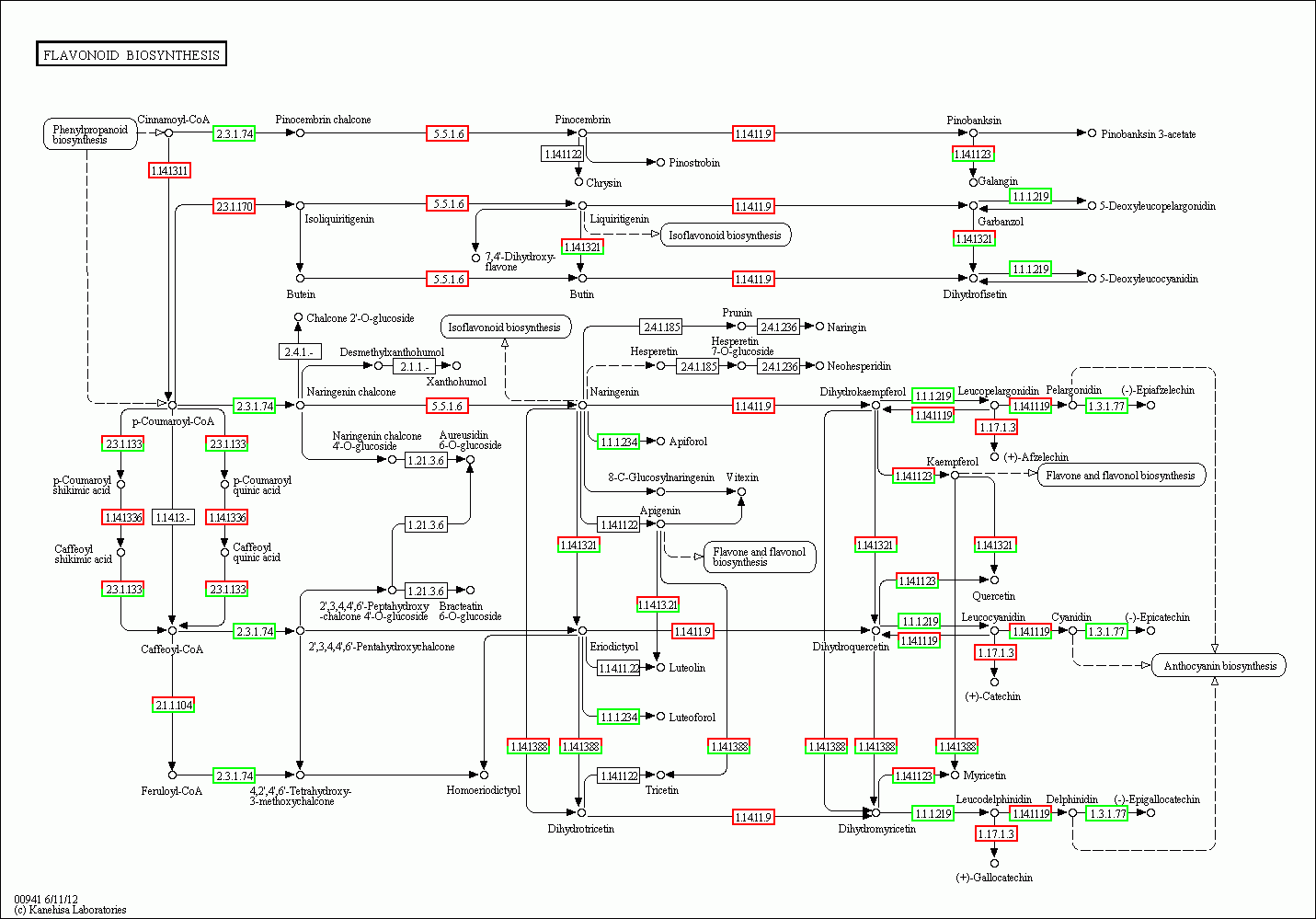

Supplement: Additional file 17: — KEGG analysis of differentially expressed genes between 72 h and 0 h (level3). [file 12864_2015_1373_MOESM17_ESM.zip › Additional file 5. KEGG analysis of differentially expressed genes between 72 h and 0 h (level3)/map00941.png]

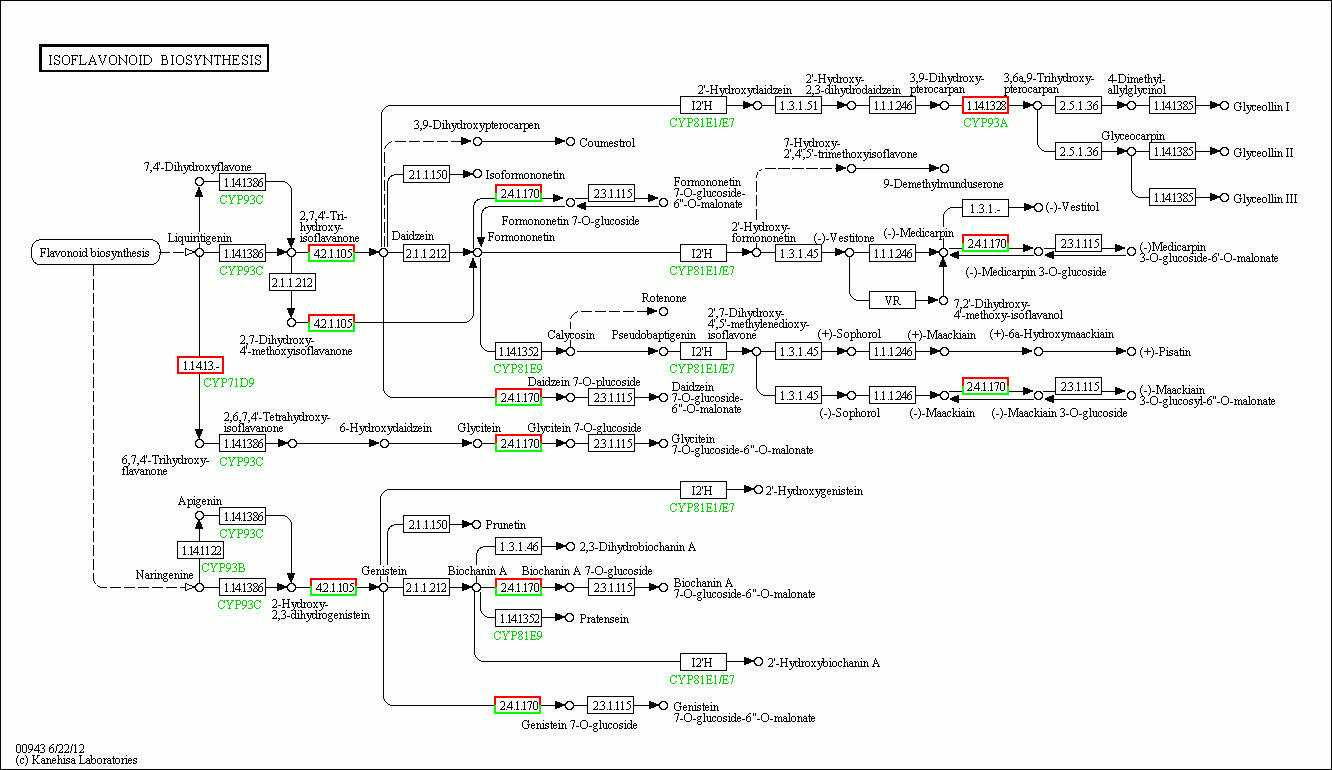

Supplement: Additional file 17: — KEGG analysis of differentially expressed genes between 72 h and 0 h (level3). [file 12864_2015_1373_MOESM17_ESM.zip › Additional file 5. KEGG analysis of differentially expressed genes between 72 h and 0 h (level3)/map00943.png]

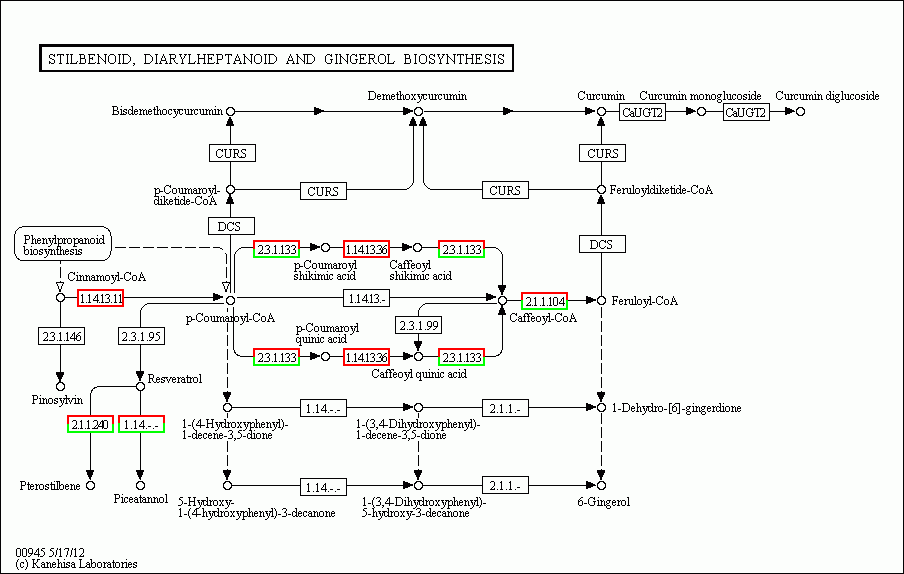

Supplement: Additional file 17: — KEGG analysis of differentially expressed genes between 72 h and 0 h (level3). [file 12864_2015_1373_MOESM17_ESM.zip › Additional file 5. KEGG analysis of differentially expressed genes between 72 h and 0 h (level3)/map00945.png]

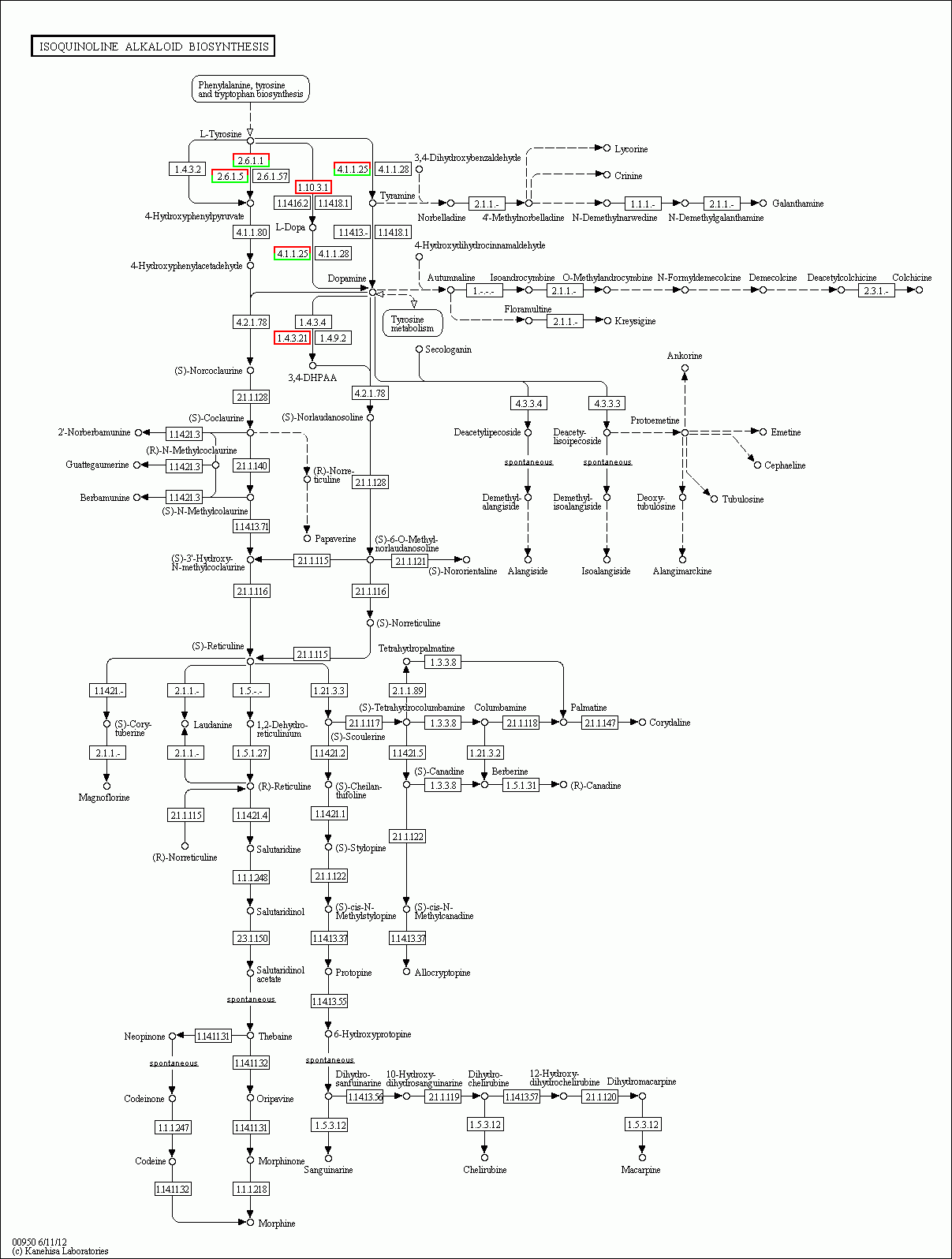

Supplement: Additional file 17: — KEGG analysis of differentially expressed genes between 72 h and 0 h (level3). [file 12864_2015_1373_MOESM17_ESM.zip › Additional file 5. KEGG analysis of differentially expressed genes between 72 h and 0 h (level3)/map00950.png]

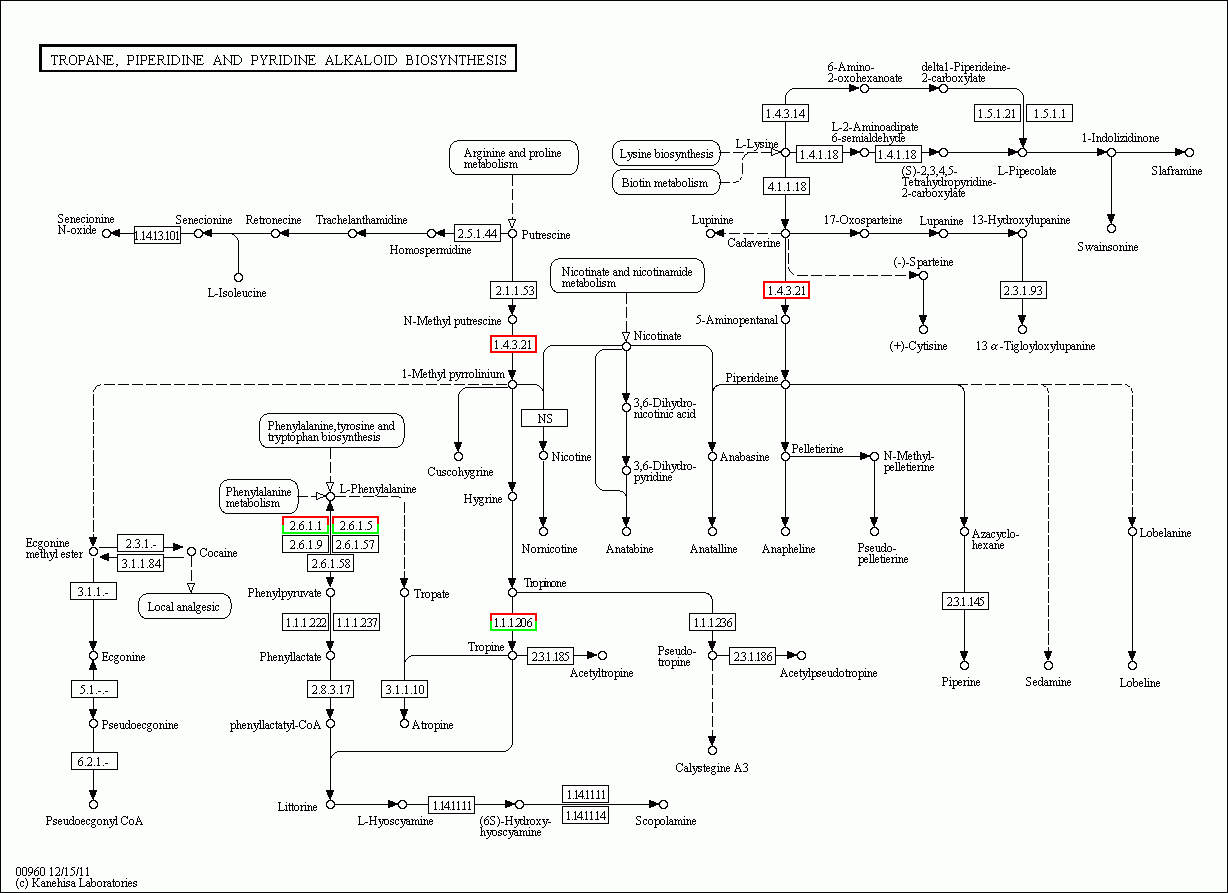

Supplement: Additional file 17: — KEGG analysis of differentially expressed genes between 72 h and 0 h (level3). [file 12864_2015_1373_MOESM17_ESM.zip › Additional file 5. KEGG analysis of differentially expressed genes between 72 h and 0 h (level3)/map00960.png]

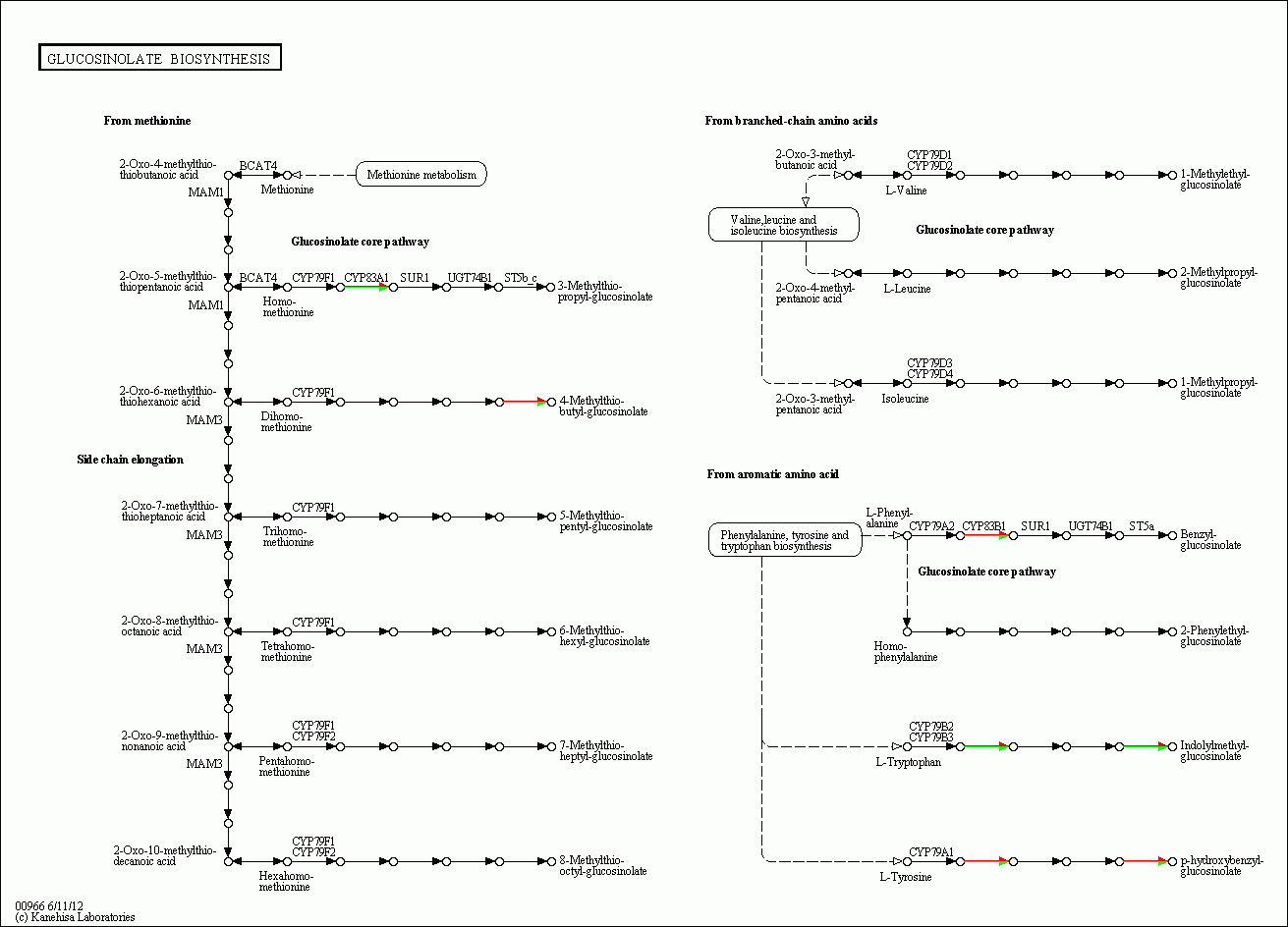

Supplement: Additional file 17: — KEGG analysis of differentially expressed genes between 72 h and 0 h (level3). [file 12864_2015_1373_MOESM17_ESM.zip › Additional file 5. KEGG analysis of differentially expressed genes between 72 h and 0 h (level3)/map00966.png]

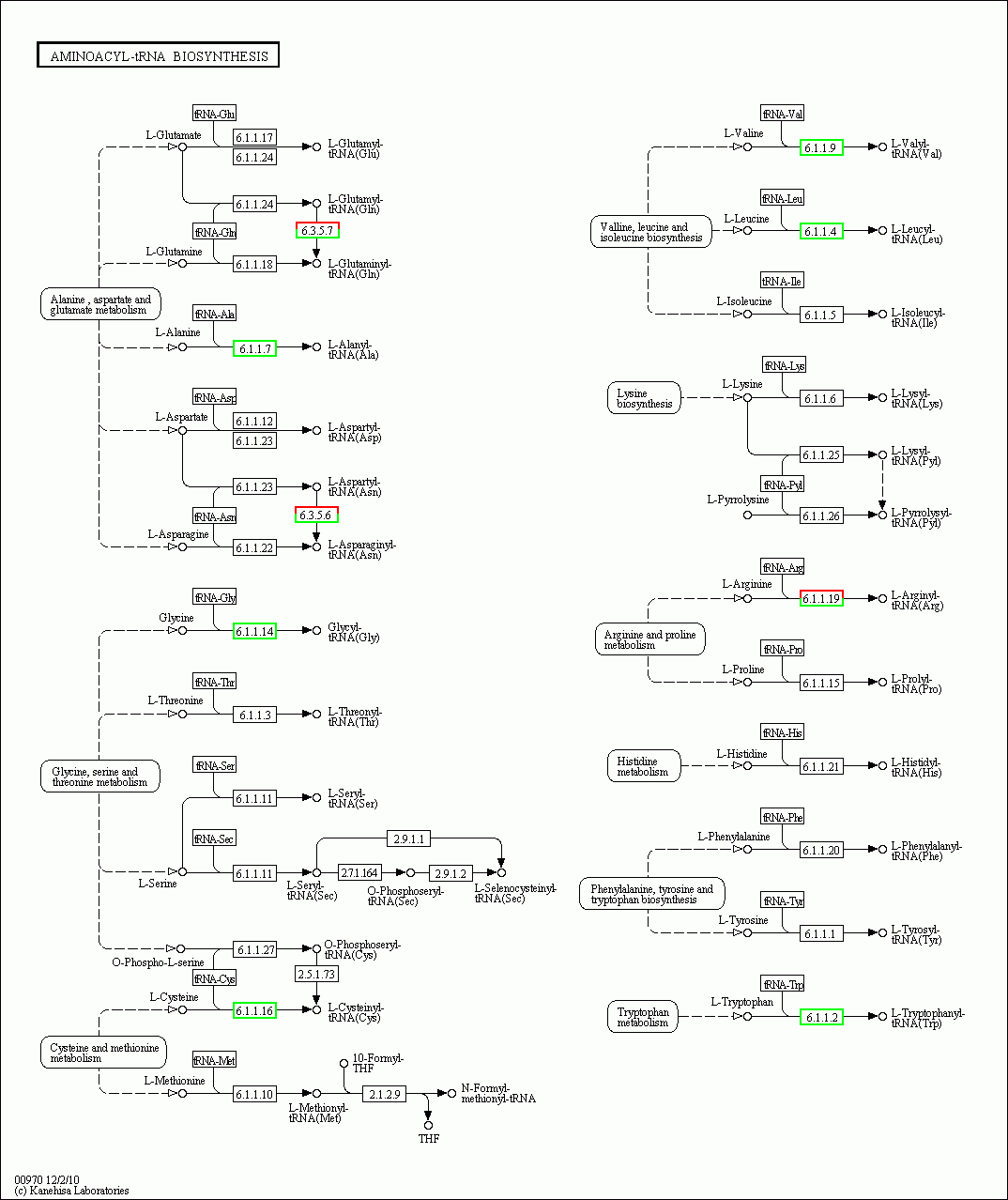

Supplement: Additional file 17: — KEGG analysis of differentially expressed genes between 72 h and 0 h (level3). [file 12864_2015_1373_MOESM17_ESM.zip › Additional file 5. KEGG analysis of differentially expressed genes between 72 h and 0 h (level3)/map00970.png]

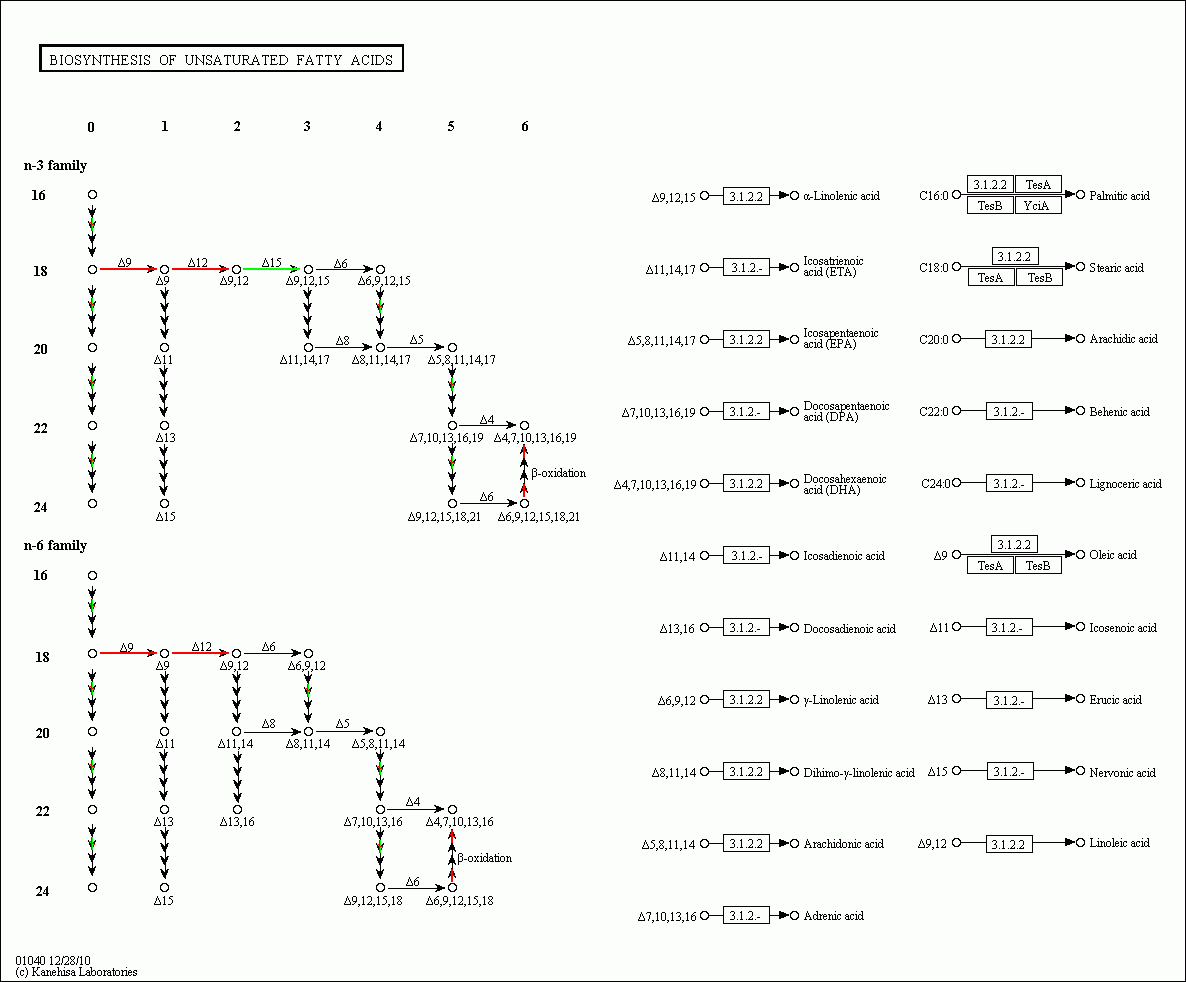

Supplement: Additional file 17: — KEGG analysis of differentially expressed genes between 72 h and 0 h (level3). [file 12864_2015_1373_MOESM17_ESM.zip › Additional file 5. KEGG analysis of differentially expressed genes between 72 h and 0 h (level3)/map01040.png]

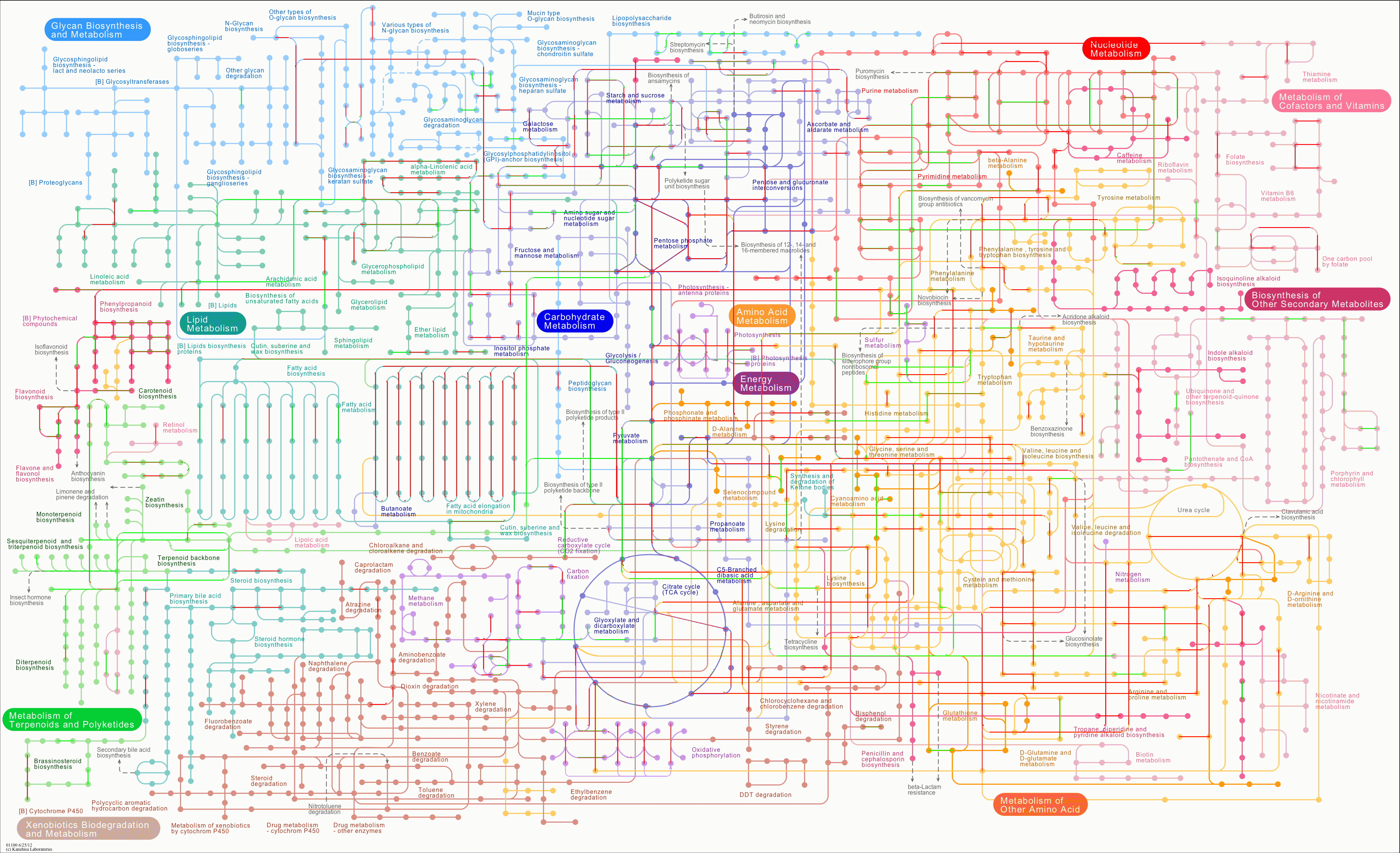

Supplement: Additional file 17: — KEGG analysis of differentially expressed genes between 72 h and 0 h (level3). [file 12864_2015_1373_MOESM17_ESM.zip › Additional file 5. KEGG analysis of differentially expressed genes between 72 h and 0 h (level3)/map01100.png]

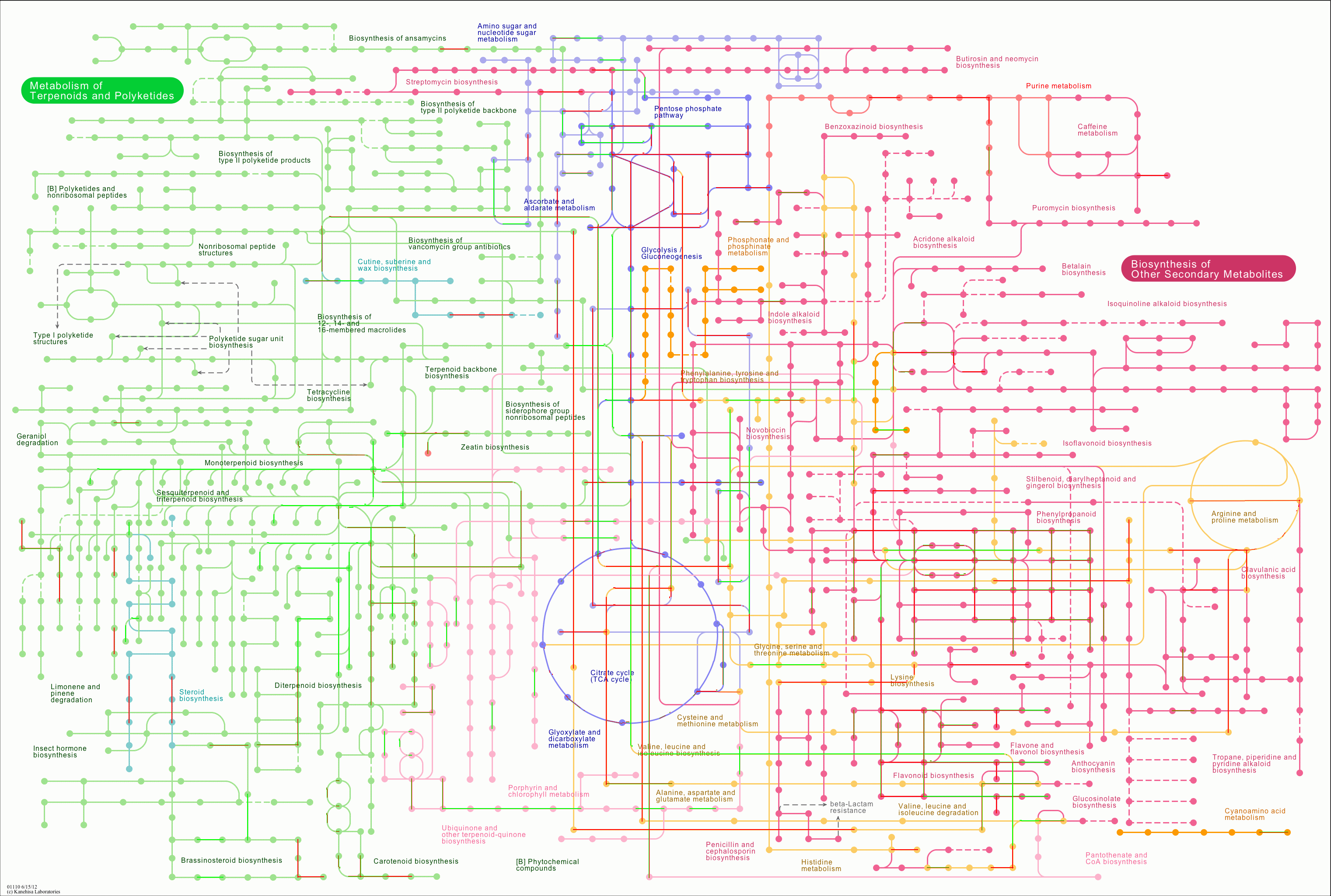

Supplement: Additional file 17: — KEGG analysis of differentially expressed genes between 72 h and 0 h (level3). [file 12864_2015_1373_MOESM17_ESM.zip › Additional file 5. KEGG analysis of differentially expressed genes between 72 h and 0 h (level3)/map01110.png]

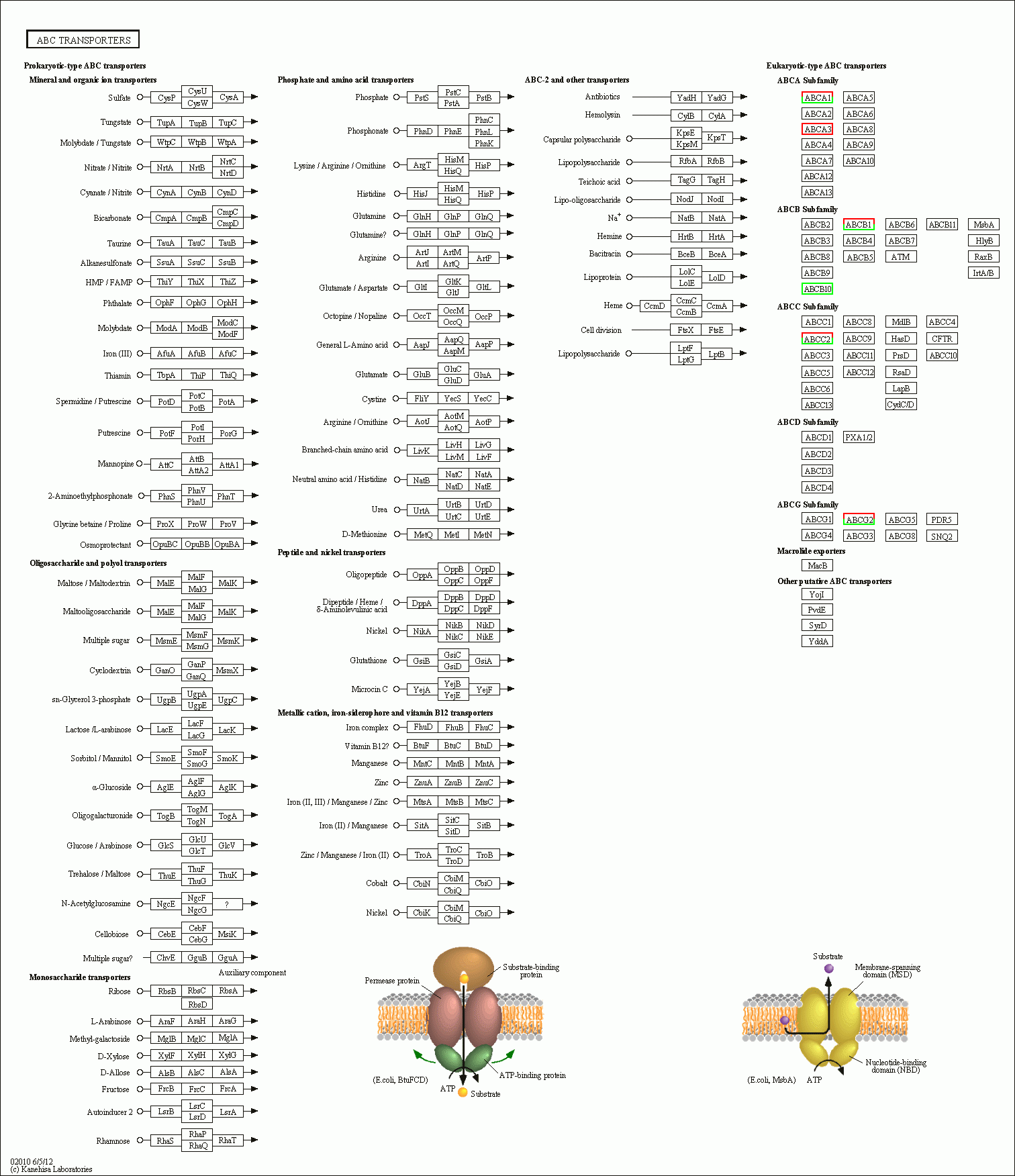

Supplement: Additional file 17: — KEGG analysis of differentially expressed genes between 72 h and 0 h (level3). [file 12864_2015_1373_MOESM17_ESM.zip › Additional file 5. KEGG analysis of differentially expressed genes between 72 h and 0 h (level3)/map02010.png]

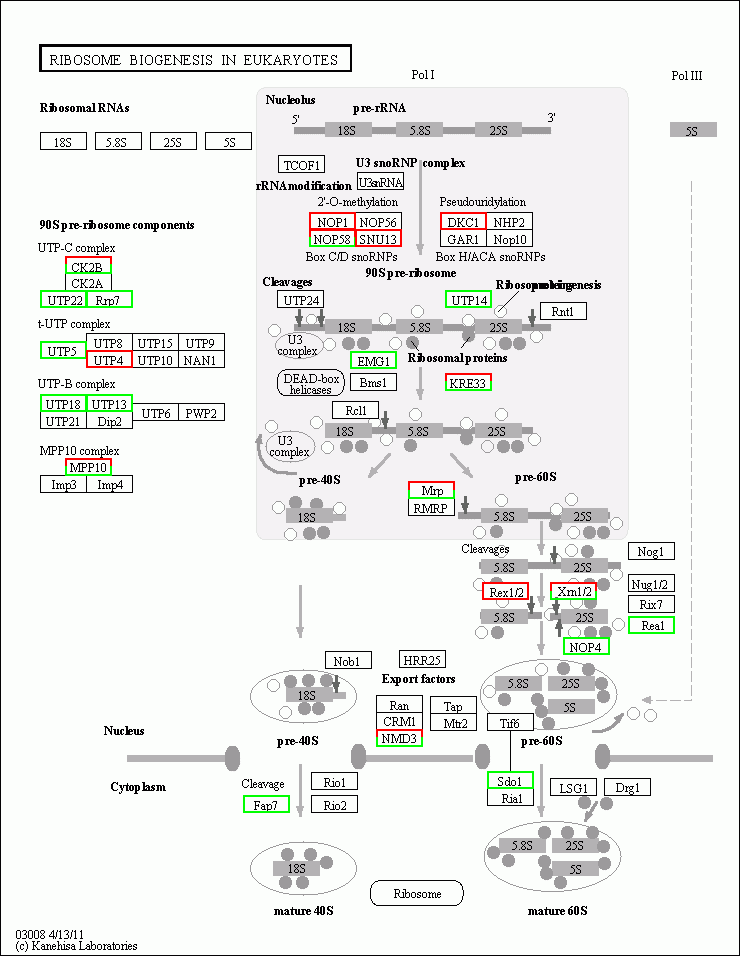

Supplement: Additional file 17: — KEGG analysis of differentially expressed genes between 72 h and 0 h (level3). [file 12864_2015_1373_MOESM17_ESM.zip › Additional file 5. KEGG analysis of differentially expressed genes between 72 h and 0 h (level3)/map03008.png]

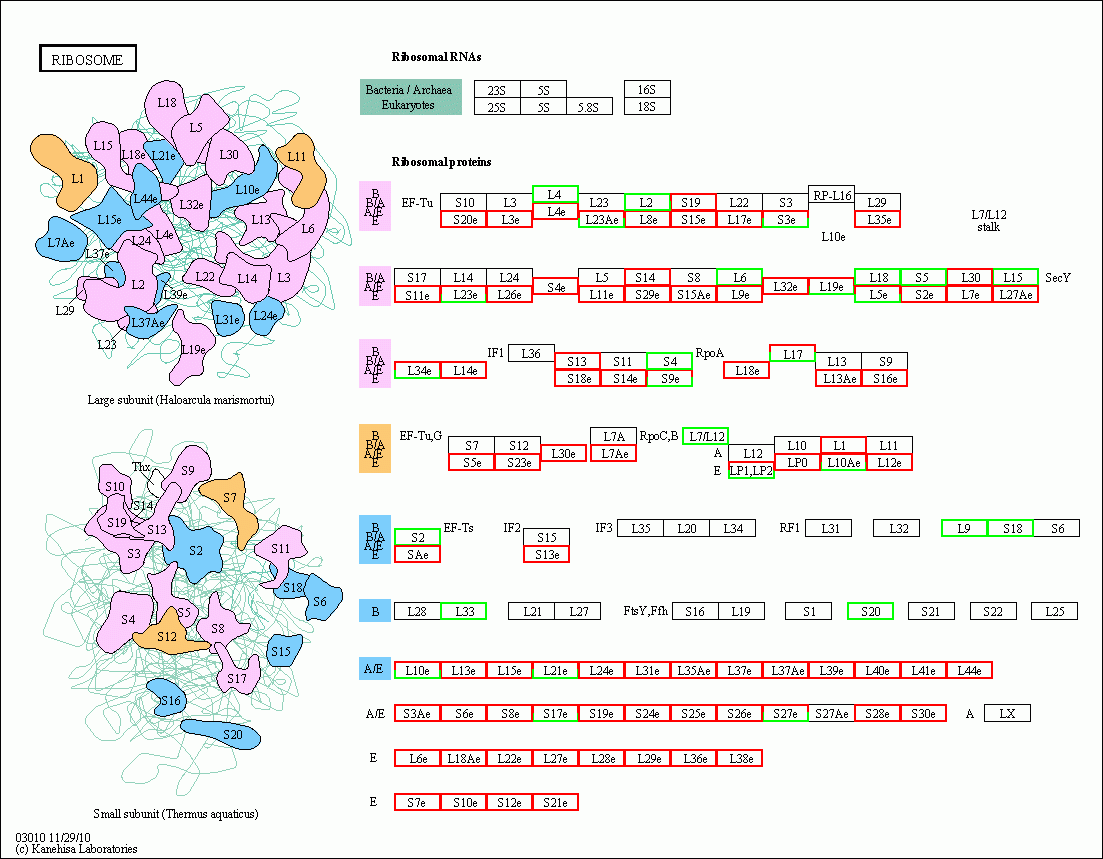

Supplement: Additional file 17: — KEGG analysis of differentially expressed genes between 72 h and 0 h (level3). [file 12864_2015_1373_MOESM17_ESM.zip › Additional file 5. KEGG analysis of differentially expressed genes between 72 h and 0 h (level3)/map03010.png]

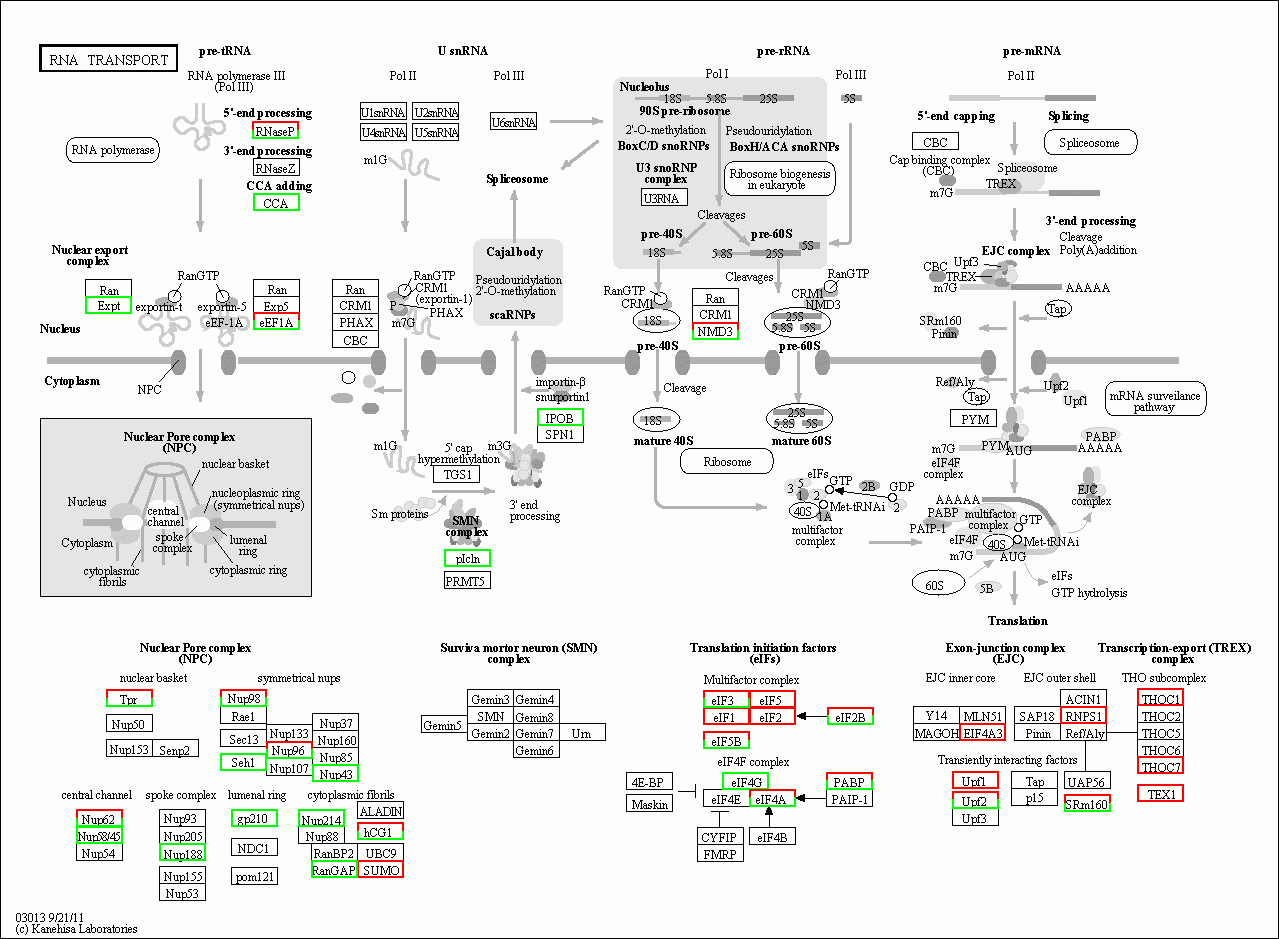

Supplement: Additional file 17: — KEGG analysis of differentially expressed genes between 72 h and 0 h (level3). [file 12864_2015_1373_MOESM17_ESM.zip › Additional file 5. KEGG analysis of differentially expressed genes between 72 h and 0 h (level3)/map03013.png]

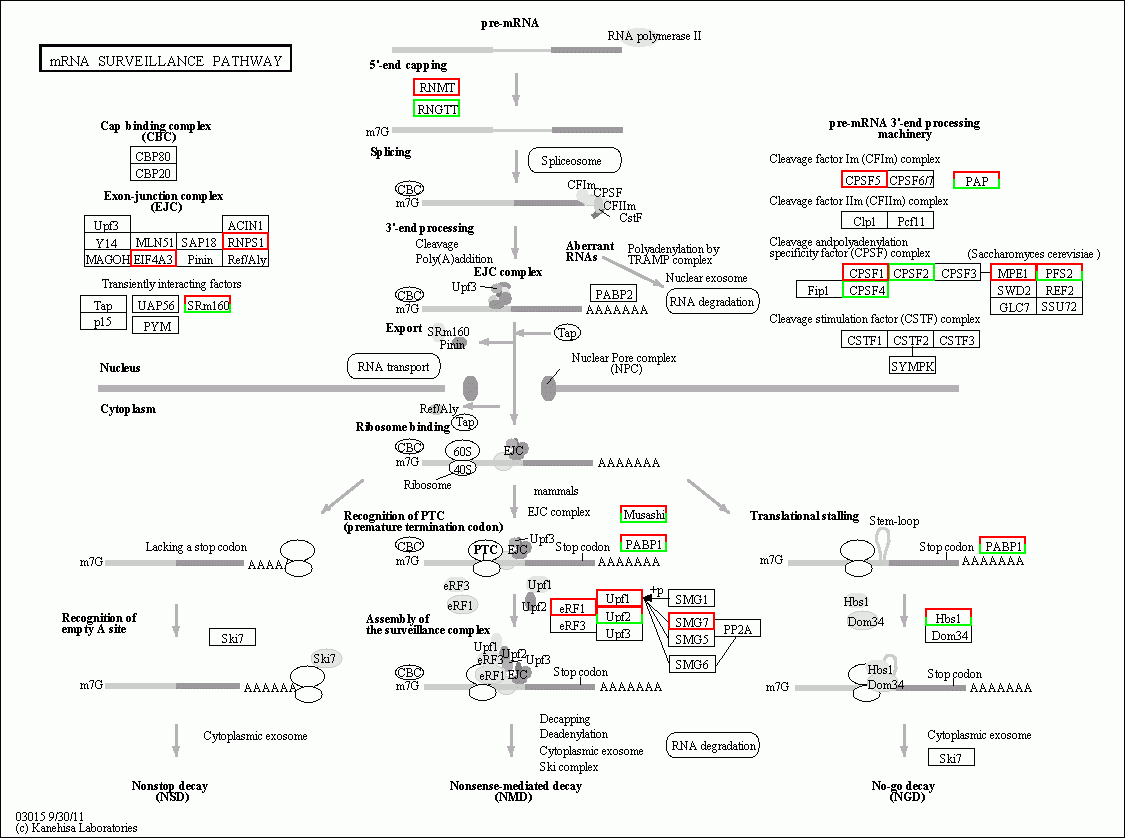

Supplement: Additional file 17: — KEGG analysis of differentially expressed genes between 72 h and 0 h (level3). [file 12864_2015_1373_MOESM17_ESM.zip › Additional file 5. KEGG analysis of differentially expressed genes between 72 h and 0 h (level3)/map03015.png]

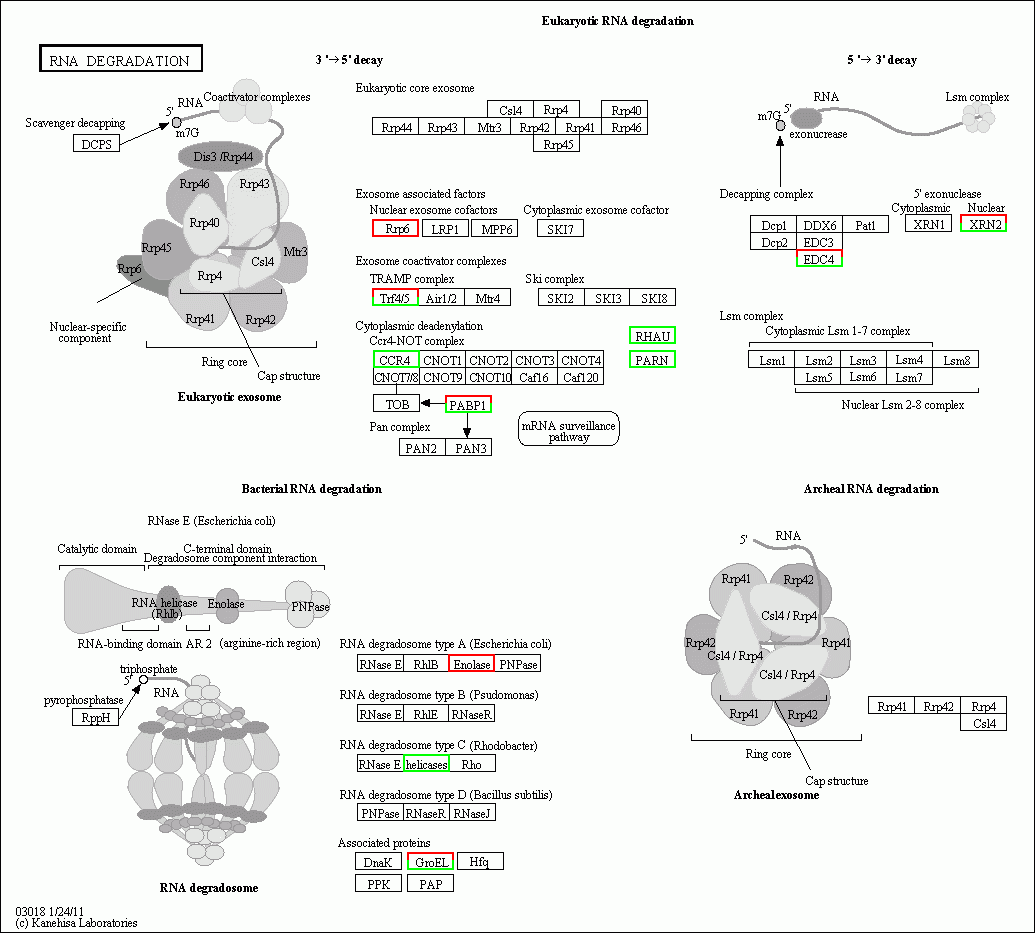

Supplement: Additional file 17: — KEGG analysis of differentially expressed genes between 72 h and 0 h (level3). [file 12864_2015_1373_MOESM17_ESM.zip › Additional file 5. KEGG analysis of differentially expressed genes between 72 h and 0 h (level3)/map03018.png]
