# Supplementary material for: Transcriptomic profiling of the salt-stress response in the halophyte Halogeton glomeratus
Source: BMC Genomics. 2015 Mar 11;16(1):169. doi: 10.1186/s12864-015-1373-z (PMC4363069; doi:10.1186/s12864-015-1373-z)
Supplement: Additional file 18: Table S1. — RT-PCR primers. [file 12864_2015_1373_MOESM18_ESM.doc]

Additional table 1. qRT-PCR primers.

| Gene ID | Forward primer（5'---3'） | Reverse primer(5'---3') | Product size (bp) |
| --- | --- | --- | --- |
| Actin | CGTGCTCAGTGGTGGTACAA | GTGCCACGACCTTGATCTTC | 104 |
| CL1857.Contig2_All | ACCACCTACCCCCACCGCAT | AACAAAAGAGGCGGCGGAGG | 108 |
| Unigene22070_All | CATGTCCAGGATGTTCAACA | GTCCACCAGAAAGTGCTCAC | 150 |
| Unigene34212_All | GTGCCTGCGAGAGAACGTCC | TGCTGGTAACGCTCACGCTG | 102 |
| Unigene34026_All | ACGCCAAGCACTCCGAGATG | GCTGGTGGCTTCGCTGGTAC | 150 |
| CL3173.Contig2_All | CACCAGGACCTCAACGAGCA | CAAAGTTCAGCGCTCCCCTC | 112 |
| CL6194.Contig1_All | GTCTCCATCGTCTTCCGCTG | CCGACGATCCTGAAGACGTT | 101 |
| Unigene2702_All | CCTACACCGCCGTGGTGAAC | TTGCGGTCGATGATGTCCTG | 112 |
| CL278.Contig2_All | AAGACGCCAGGTTTGCTGCG | GCTGGAGCGCCACTGATCCT | 104 |
| Unigene24542_All | GACCGACGTGACCTACGACG | GTGCCTGATCTTGAGCCCCT | 118 |
| Unigene19538_All | GCTCAGTGCGATCCGTGAAA | CTTGAAGGTGTTGCCGGTCA | 108 |
| Unigene24964_All | TCCACGTCCGCAGGAAGTAG | GCGAAGTGCTGCACAGGTTT | 134 |
| CL5601.Contig1_All | GCCGTTATTGTTGCGTTGCT | ACAGCCTTGCTTGCCATCAC | 106 |
| CL1103.Contig2_All | CCAGCGAAATTCTGTGCGAC | AGCCTGAACAACCCGTCCTC | 126 |
| CL532.Contig1_All | ATCATGTTGTGGACGCAGCC | CGAGCCCCACATCTACTGCA | 102 |
| Unigene34281_All | CCCGTCCGACAGACCAAGTC | CGGTAGACCTTCTCGACGCC | 160 |
| Unigene5419_All | ATGCGATGCCTGAAAACTCG | TACACATTTGGAGCTGCCCC | 104 |
| Unigene33828_All | TCCTCCTTGTGCCTGTCTGA | GGTAAGCGTTCGTCCTCGTT | 118 |
| CL39.Contig1_All | AATGCAGCGCAGTACCCTTG | AAGGCTTTTCCTGCAGCACC | 101 |
