# Supplementary material for: Transcriptomic profiling of the salt-stress response in the halophyte Halogeton glomeratus
Source: BMC Genomics. 2015 Mar 11;16(1):169. doi: 10.1186/s12864-015-1373-z (PMC4363069; doi:10.1186/s12864-015-1373-z)
Supplement: Additional file 13: — KEGG analysis of all unigenes (level 3). [file 12864_2015_1373_MOESM13_ESM.zip › Additional file 1. KEGG analysis of all unigenes (level 3)/map04145.html]

map04145
